# Telomere-to-telomere co-mapping of transposable elements and human genes identifies a cluster of young L1 elements in the interferon-alpha domain with potential evolutionary arms-race

**DOI:** 10.1093/g3journal/jkag250

**Published:** 2026-09-23

**Authors:** Daniil Nikitin

**Affiliations:** Institute of Molecular Biology, National Academy of Science of the Republic of Armenia, Yerevan 0014, Republic of Armenia

**Keywords:** transposable elements, T2T-CHM13, transcription start site, gene ontology, interferon alpha, LINE-1, human genome

## Abstract

Transposable elements (TEs) have played a central role in major evolutionary transitions across the human lineage, from eukaryogenesis to the emergence of the eutherian placenta, and are currently reactivated in cancer and autoimmune diseases. The complete telomere-to-telomere (T2T) assembly enables comprehensive investigation of TE contributions to gene regulation. Using a 10 kb window around transcription start sites, we mapped 3,709,429 human TEs to 28,738 genes against a 500-permutation background and assessed the enrichment and functional associations of 6 TE classes and 44 families. We identified a 220 kb interferon-alpha domain enriched with young L1 elements: 77 of its 175 TEs are evolutionary young L1 copies from 36 subfamilies, at mean divergence 135.7 against 188.2 genome-wide. This is consistent with recent L1 activity and could indicate a recent evolutionary arms-race affecting innate immune gene regulation. Distinct TE classes exhibited specific functional associations: SVA elements were enriched near genes involved in transcription termination; Alu elements were linked to RNA processing and splicing; MIR elements were associated with zinc homeostasis and voltage-gated potassium channel genes; LINE elements were enriched near genes related to lipid metabolism and olfactory perception; and LTR elements were associated with ion transport. This correlative map provides a positional baseline against which epigenomic and functional TE studies can be normalized, and a set of candidate loci for further investigation of evolutionary arms race.

## Introduction

The release of the first complete, gapless human genome assembly (T2T-CHM13) by the telomere-to-telomere consortium has fundamentally shifted the paradigms of evolutionary genomics (Nurk et al. 2022). For over 2 decades, genomic analysis relied on references such as GRCh38, which contained hundreds of megabases of unresolved sequences, primarily concentrated in highly repetitive regions like centromeres, subtelomeres, and acrocentric short arms. These “dark regions” of the genome are disproportionately enriched with transposable elements (TEs), the selfish genetic entities that comprise more than half of the human DNA (Hoyt et al. 2022). The transition from GRCh38 to T2T-CHM13 involved the resolution of ∼238 million base pairs of formerly unmasked DNA, which revealed a significantly higher repeat content than previously estimated (Nurk et al. 2022). This resolved sequence is dominated by tandemly arrayed repeats, such as alpha satellites in centromeric regions, but also contains a vast number of TEs nested within complex genomic structures (Hoyt et al. 2022).

TEs are increasingly recognized as drivers of regulatory innovation during the major evolutionary transitions from eukaryogenesis to eutherian placenta and human neocortex (Nikitin 2026b). Moreover, TEs proliferate in a wave pattern within the human genome, and they have been described to be in a continuous evolutionary arms race with host defense systems (Betancourt et al. 2024). During this process TEs insert their transcription factor binding sites in the vicinity of host genes and alter their expression (Nikitin et al. 2018, 2019b).

There are dozens of studies of this epigenetic impact which rely on different methodology but are close in the main approach, namely proximity mapping: TEs mapped in the vicinity of human genes are likely to impact their expression (Bourque et al. 2008; Chuong et al. 2016; Ito et al. 2017; Correa et al. 2021; Kosuge et al. 2024). Despite the same principle, different methodologies are difficult to be compared: genomic proximity windows vary from 4 to 20 kb around a gene, the statistical frameworks and epigenomic modalities differ significantly. Moreover, all these studies are based either on hg19 or hg38 human genome assemblies. A unified proximity map built on the most up-to-date T2T human genome assembly could provide the positional baseline these studies can be compared with: a common measure of how close each TE group locates to each functional gene group, against which epigenomic and expression signals can be normalized.

Here we have taken the most widely used proximity window of 10 kb around human transcription start sites (TSSs) of 28,738 unique human T2T-annotated genes and we have built a high-resolution functional map of TE enrichment at the level of TE classes and families, taking 3,709,429 individual elements into analysis. We show that SVA elements are enriched near genes responsible for transcription termination, Alu elements are co-associated with RNA processing and splicing genes, MIR repeats relate to genes responsible for zinc, copper and cadmium detoxification, LINE elements are enriched near genes of lipid metabolism and sensory perception of smell, and LTR elements near genes of ion transport. We show that the 220 kb interferon alpha domain is enriched with young, low-divergence L1 elements, identifying a candidate locus of recent L1 activity for functional follow-up studies. Furthermore, embryogenesis and nervous system genes were relatively depleted with TE insertions constituting evolutionary conservative processes. Finally, synaptic transmission and nervous system development were enriched with evolutionary ancient TEs.

This comprehensive proximity analysis serves as a critical baseline for understanding the functional impact of TEs, as most TE-host interactions, including enhancer exaptation and promoter birth, occur within these 10 kb proximal windows. Albeit the current analysis is purely correlative and cannot detect the ongoing evolutionary arms race, it points at genome loci and molecular processes that could be impacted by it for future investigation.

## Materials and methods

### Human TEs

Coordinates and class/family annotations for human TEs were obtained from the RepeatMasker track (Tarailo-Graovac and Chen 2009) based on the T2T human genome assembly, using the Table Browser tool (Group: *Variation and Repeats*, Track: *RepeatMasker*, Table: *T2T RepeatMasker*). The T2T RepeatMasker annotation itself was derived from (Hoyt et al. 2022), and average divergence scores (number of substitutions per 1,000 base pairs) were extracted from the same UCSC RepeatMasker track.

Each TE was categorized by RepeatMasker into hierarchical levels of classification (Tarailo-Graovac and Chen 2009):

- Class, representing the highest level, defined by the mechanism of transposition.
- Family, representing an intermediate level, grouping elements of shared evolutionary origin that typically exhibit similar structural features and transposition mechanisms (eg Alu and L1 elements).

In this study, we adopted the RepeatMasker classification as provided in the T2T genome assembly without modification, ensuring consistency with the widely accepted TE annotation framework.

The processed annotation used throughout is provided as the tab-separated file T2T_repeat_masker_processed_sorted.bed (chromosome, start, end, divergence score, subfamily, family, class), and the TSS neighborhoods as T2T_genes.bed. These 2 files are the sole inputs to every intersection reported here, including the permutation background and the window sensitivity analysis. The files are deposited in the GitHub repository accessible via the link https://github.com/Nikit357/T2T_transposons_genes.

The dataset comprised elements assigned to the following classes: LINE, SINE, LTR, SVA (annotated as “Retroposon” in the source table), Helitron (denoted as “RC” for rolling-circle elements), and DNA transposons. In total, the dataset included 3,709,429 entries: 1,706,485 SINEs, 1,005,214 LINEs, 531,410 LTRs, 458,177 DNA elements, 6,274 SVAs, and 1,869 Helitrons.

Although SVA elements are evolutionarily and functionally related to Alu elements (Jacobs et al. 2014), SINEs and SVAs were treated as distinct classes in accordance with RepeatMasker nomenclature. Likewise, Helitrons were considered as a separate TE class, despite their mechanistic classification as a subset of DNA transposons due to their rolling-circle replication mechanism (Barro-Trastoy and Köhler 2024).

### Human genes and TSS

Human genes coordinates were downloaded from UCSC Genome Browser (Perez et al. 2025) via the *Table Browser* tool, using the January 2022 assembly (T2T CHM13v2.0/hs1). Data were extracted from the group *Genes and Gene Predictions*, track *NCBI RefSeq*, and table *RefSeq All*. Only curated gene entries were retained by filtering for accessions beginning with prefixes NM, NR, NP, or YP. TSS coordinates were defined as the leftmost exon start (5′ UTR exon) for genes on the positive strand and the rightmost exon end for genes on the negative strand. The 10 kb promoter regions (TSS neighborhoods) were then constructed by extending 5 kb upstream and downstream of each TSS.

HUGO gene symbols (column geneName2) were used as primary identifiers, yielding 28,738 unique genes. Transcript isoforms corresponding to the same gene but differing in TSS position were treated as distinct entities during epigenomic profiling and RE mapping. The complete set of TSS 10 kb neighborhoods used in the mapping is provided in Supplementary File 1, sheet TSS_TE_intersections (38,704 entries including isoforms).

### Mapping of TEs on gene TSS neighborhoods

The mapping of TE coordinates with their divergence onto gene TSS 10 kb neighborhoods was done using BEDTools (BEDTools map utility, version 2.31.1) (Quinlan and Hall 2010), any TEs that have been intersecting with the 10 kb interval for a given gene were taken into the analysis. This could lead to artificial underestimation of enrichment and regulatory impact of longer TE classes (LINEs and LTR elements), so the appropriate random permutation controls were applied as described later.

The 10 kb window (5 kb upstream and 5 kb downstream of each TSS) was chosen because it is the most widely used proximity window in the functional TE literature and therefore makes this map comparable to it. A study of interferon-inducible enhancers spread by LTR elements used a 10 kb window (Chuong et al. 2016); an analysis of TE content around duplicated and singleton genes used 4 and 20 kb windows (Correa et al. 2021); one of the first surveys of TE-linked transcription factor binding used 5 kb either side of coding exons (Bourque et al. 2008); a proximity study of LTR function used a shifted 6 kb window (5 kb upstream, 1 kb downstream) (Ito et al. 2017); and our own transcription-factor and histone-modification studies of retroelements used the same 10 kb window applied here (Nikitin et al. 2019a).

Because the 10 kb window choice is conventional, the full enrichment analysis was repeated for TSS neighborhoods of 5 kb (2.5 kb either side) and 20 kb (10 kb either side), built from the same TSS definition.

### Random control of TEs enrichment

To control TE enrichment near genes against a random background and remove a potential length bias, we performed 500 random permutations of TE coordinates with the BEDTools shuffle command, using random seeds 1 to 500. An empirical *P*-value was calculated as fraction of random odds ratios (ORs) that were above or below the observed one, depending on whether the observed OR was below or above the median across the 500 random OR values. Then the empirical *P*-value was lower clipped by 2/(*N* + 1), where *N* was the permutations number (500), which places the lower bound at 0.004. An enrichment score was calculated as a fold change of observed vs random ORs. Since reporting the observed OR alone would systematically under-call short elements, every enrichment statement in this work is consequently made on this enrichment score.

Five hundred permutations were considered sufficient as recomputing the random OR from truncated parts of the permutation set shows that by *N* = 140 the running mean differs from the *N* = 500 value by <1% (Supplementary Fig. 1a), and standard deviation at *N* = 250 differs by <6% of its *N* = 500 value (Supplementary Fig. 1b). At the level of families, after *N* = 315 standard deviation of the least stable family was below 10% of the final *N* = 500 one (Supplementary Fig. 1c). The checkpoint values for the random permutation curves are provided in the repository.

The 220 kb domain on chromosome 9 was separately tested against 10,000 random windows of the same length drawn from a pool of 120,000 autosomal windows (random seed 42). Three nested null models were used, each progressively better matched to the interferon-alpha domain: windows containing at least one L1 element; windows containing at least 40 L1 elements, which controls for local L1 density; and windows containing at least 10 annotated genes and at least one L1, which controls for gene density. In each case the statistic was the mean divergence of the L1 elements in the window and the 2-sided empirical *P*-value was the fraction of null windows at least as extreme as the observation. A fourth test compared the young-primate-specific (L1HS and L1P*) against the older mammalian (L1M*) composition of the domain with the rest of the genome by Fisher exact test. Leave-one-out means were computed to confirm that no single element drives the result.

### Statistical tests

Group comparisons were done by Mann–Whitney *U* test (Mann and Whitney 1947) implemented in scipy python library (Virtanen et al. 2020), version 1.17.1. Multiple hypotheses were corrected by the false discovery rate (FDR) approach (Benjamini and Hochberg 1995) implemented in the python statsmodels library (Seabold and Perktold 2010), version 0.14.6. OR and *P*-values for TE classes and families' enrichment in the TSS 10 kb of genes, as well as enrichments of Gene Ontology (GO) terms in TE groups by functional group were calculated using Fisher exact test (Mays and Stark 2026) in scipy. Linear correlation coefficients and their *P*-values were calculated by Pearson (Waldmann 2019). Distributions were compared by Kolmogorov–Smirnov test (Cardoso and Galeno 2023) implemented in scipy. FDR-corrected *P*-value 0.05 was selected as a threshold value for Mann–Whitney, Fisher exact and Kolmogorov–Smirnov tests for multiple hypotheses.

Agreement between window sizes (5, 10, and 20 kb) and gene percentiles (top 5% and 10%) was measured by Spearman and Pearson correlation (Waldmann 2019) of the observed to random OR, by Bland–Altman comparison (Giavarina 2015), by the overlap coefficient and a hypergeometric test for the gene sets (Falcon and Gentleman 2008), by Kendall rank correlation (Cartwright 1957) with a bootstrap confidence interval for the gene rankings, and by a label-shuffling permutation test of the observed correlation.

Unless stated otherwise, all reported *P*-values are Benjamini–Hochberg FDR-adjusted, and *P*-values that are raw are labeled as such in the figure captions and tables. The Kolmogorov–Smirnov comparisons of divergence and length distributions, the Pearson correlations of GO term counts against enrichment statistics, and the interferon-alpha domain permutation tests are single tests per panel and are reported raw. Both the raw and the adjusted value are provided for every test in the supplementary tables: enrichment_families_with_random.csv and enrichment_subfamilies_with_random.csv in the GitHub repository (https://github.com/Nikit357/T2T_transposons_genes) carry Enrichment_p_value and Enrichment_p_value_adjusted alongside p_raw_empirical and p_adjusted_empirical_bh, and the GO tables carry both *P*-value and FDR.

### GO analysis

TSS for isoforms of the same gene were considered as distinct unique entities, with enrichment, divergence and epigenomic analyses performed independently for them. For the GO analysis gene names were deduplicated among the TSS that were extracted as enriched by a certain criterium. GO analysis was performed using goatools python library (Klopfenstein et al. 2018), version 1.6.4 on a local database downloaded on 2025 December 31. FDR-corrected Fisher exact test *P*-value 0.05 was selected as a threshold value for GO terms, the GO terms at the borderline of FDR *P*-value between 0.05 and 0.1 were extracted separately.

The GO analysis was repeated with all the 3 TSS window sizes (the main 10 kb and the comparator sizes 5 and 20 kb) for the top and bottom 10% of genes (2,872 genes at maximum) alongside the 5% sets (1,436 genes at maximum) used in the main analysis, at the same FDR threshold.

### Visualization

Plots for this article were drawn using matplotlib version 3.10.8 (Hunter 2007), plotly version 6.7.0 (Plotly Inc.), statannotations version 0.7.2 (Florian 2020) and seaborn version 0.13.2 (Waskom 2021) libraries in Python 3.11. Supervenn plots were built using the supervenn python library (gecko984 2022), version 0.5.0. Network visualizations were built using networkx version 3.6.1 (Hagberg et al. 2008) and pyvis version 0.3.2 (Perrone Gary et al. 2020) libraries in python.

UCSC Genome Browser tracks were visualized using the Genome Browser web portal (Casper et al. 2026).

### Data analysis

Data analysis was performed in a Jupyter Notebook environment using pandas version 3.0.2 (The pandas development team) and numpy version 2.4.4 (Harris et al. 2020) for tabular data handling and analysis.

### AI usage

Gemini 2.5 PRO (Comanici et al. 2026) and Claude Code Opus 5 (Anthropic 2026) were used for code refining, assistance with literature search and pre-classification of GO terms into large biological groups. ChatGPT was used for grammar corrections of the manuscript (Biswas 2023).

All code writing was done in a plan-then-act mode: the AI agent first wrote a research and implementation plan, the author then reviewed and commented on it, and after 2 to 5 review cycles the agent implemented the requested feature. AI specifications can be found in the project repository (https://github.com/Nikit357/T2T_transposons_genes) as CLAUDE.md files in all the subfolders. The intermediate research outputs and implementation plans, as well as additional documentation are stored in other markdown files.

Reference list and in-text citations were prepared with Mendeley reference manager (Mendeley 2026), any AI tools were strictly prohibited to edit or add citations as can be validated in the Claude specification files in the repository. The final text, figures and tables were reviewed and edited by the author.

### Data and code availability

All code, intermediate tables and the permutation background are available as described in the Data availability statement.

## Results

### Mapping and enrichment of TEs on gene TSS 10 kb neighborhoods at the level of major classes

The TE coordinates (3,709,429 total entries) were mapped on gene TSS 10 kb neighborhoods (5 kb upstream and 5 kb downstream of each TSS) for 28,738 unique human genes, with 38,704 total unique TSS due to the possibility of multiple TSS per gene giving rise to isoform transcripts. TSS for isoforms were considered as distinct unique entities. The mapping showed that only 0.89% of unique TSS (343) do not intersect with TEs at the proximity level of 10 kb and can be considered as free from TE-mediated regulatory impact at this level (Fig. 1a). On average, each gene harbored 15.05 TEs in its TSS neighborhood (4.39 LINEs, 1.32 LTRs, 7.82 SINEs, 1.49 DNA elements, 0.03 SVA elements, and 0.004 Helitrons, Fig. 1b). The gene with maximum number of TEs was *CIB3* (calcium and integrin binding protein 3), having 42 TEs of various classes (17 LINEs, 23 SINEs, 1 LTR, and 1 DNA element, Fig. 1c). Lists of TEs mapped on each gene TSS, their divergence and classification, can be found in Supplementary File 1, sheet TSS_TE_intersections. Most genes had in their TSS neighborhoods at least one TE for the 4 major classes: 96.9% of all TSS had SINEs, 95.1% of TSS harbored LINEs, 68.5% had DNA elements, and 58.7% had LTRs (Fig. 1b).

**Fig. 1.**
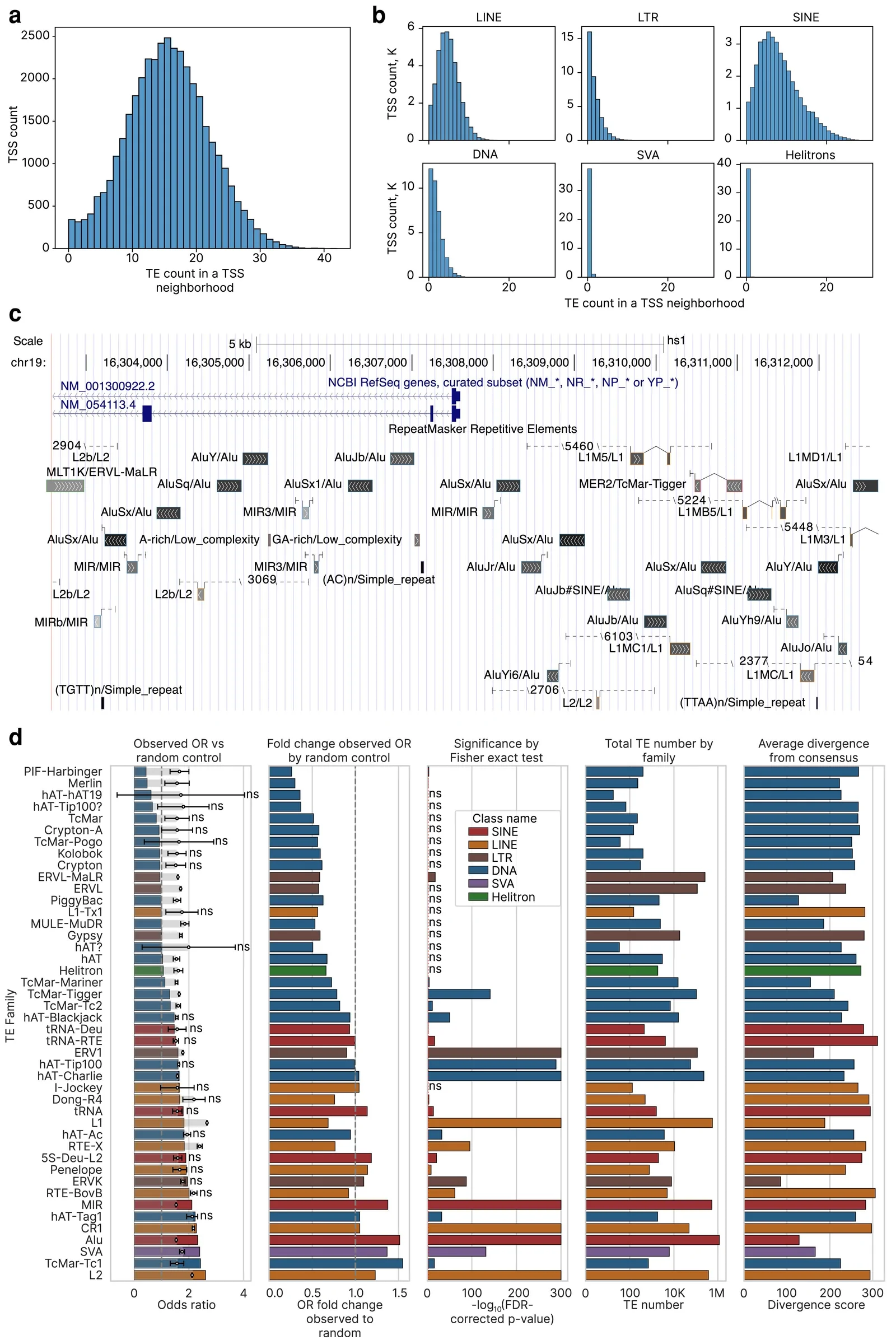
Enrichment of TEs in the 10 kb vicinity of human TSS at the level of classes and families. a) Distribution of TSS by TE count per TSS. b) Distributions of TSS by TE count of individual classes. c) UCSC Genome Browser visualization of *CIB3*, a gene having the highest TE count in its 10 kb neighborhood. d) TE families sorted by their degree of enrichment near genes TSS. The leftmost (first) vertical bar plot with bars colored by TE families shows Fisher exact test OR, faint gray bars depict the mean OR of 500 random shuffling iteration, error bars denoting their standard deviations. “ns” marks indicate those families whose empirical *P*-value (quantile of observed OR in the distribution of 500 random OR values) was higher than 0.05 in either direction after FDR correction for multiple hypothesis testing. The second vertical bar plot shows fold change of the observed OR to mean of the random ORs distribution. The third vertical bar plot visualizes the negative decimal logarithm of the FDR-corrected Fisher exact test *P*-value. The fourth and the fifth vertical bar plots show total TE number and average divergence (substitutions per 1,000 base pairs) by family, respectively.

In order to test the significance of genes TSS neighborhoods enrichment by TEs of certain classes and families, we generated 500 random permutations of all human TEs from T2T RepeatMasker (1,706,485 SINEs, 1,005,214 LINEs, 531,410 LTRs, 458,177 DNA elements, 6,274 SVAs, and 1,869 Helitrons) and intersected them with the same set of gene TSS, counting number of TEs of a given group. Significance of enrichment or deficiency of any TE group (classes and families) was assessed at the 2 levels: firstly, by OR according to Fisher exact test, and then the empirical *P*-value derived from the 500 random permutations to account for artificially higher probability of intersection for longer elements.

Tables 1 and 2 show the assessment of TE enrichment in gene TSS 10 kb neighborhoods by class: Table 1 reports the counts and the ORs, Table 2 the statistical support for them. SINEs and SVA elements were enriched in the TSS proximity by a factor of 1.468 and 1.368, respectively, whereas LINEs, LTRs, and DNA elements were depleted in the TSS neighborhoods by a factor of 0.877, 0.667, and 0.938, respectively. In general, the observed OR for all TEs was 1.95 (1.79 the random one), which was showing an enrichment by a factor of 1.086 (empirical *P* = 0.004).

**Table 1.** Enrichment of TE classes in gene TSS neighborhoods: counts and ORs.

Class name	TE count in TSS	TE count total	Odds ratio	Mean of random odds ratios	SD of random odds ratios	Observed to random odds ratio fold change
LINE	169,930	1,005,214	2.13	2.43	0.009	0.877
LTR	51,103	531,410	1.11	1.67	0.010	0.667
SINE	302,480	1,706,485	2.25	1.53	0.005	1.468
DNA	57,684	458,177	1.51	1.61	0.010	0.938
SVA	1170	6274	2.40	1.75	0.094	1.368
Helitrons	173	1869	1.07	1.61	0.163	0.661

**Table 2.** Enrichment of TE classes in gene TSS neighborhoods: supporting statistics.

Class name	Fisher *P*-value	Adjusted Fisher *P*-value	Empirical *P*-value	Adjusted empirical *P*-value
LINE	<10^−200^	<10^−200^	0.004	0.004
LTR	6.3 × 10^−112^	7.6 × 10^−112^	0.004	0.004
SINE	<10^−200^	<10^−200^	0.004	0.004
DNA	<10^−200^	<10^−200^	0.004	0.004
SVA	9.3 × 10^−133^	1.4 × 10^−132^	0.004	0.004
Helitrons	0.41	0.41	0.004	0.004

### Mapping and enrichment of TEs on gene TSS 10 kb neighborhoods at the level of families

At the level of families, a more complicated picture was observed: DNA families were mainly depleted in the vicinity of genes TSS (compared with the random OR), whereas LINEs and LTRs were less depleted or even enriched near TSS (Fig. 1d, Supplementary File 1, sheet enrichment_families). Only 7 out of 44 TE families were significantly enriched according to both tests (the Fisher exact and the permutation-based one): hAT-Charlie (DNA, 1.041 enrichment measured fold change of the observed to random OR), MIR (SINE, 1.377), CR1 (LINE, 1.051), Alu (SINE, 1.513), SVA elements (1.368), TcMar-Tc1 (DNA, 1.548), and L2 (LINE, 1.230). In contrast, 9 families were significantly depleted in the vicinity of TSS: PIF-Harbinger (DNA, 0.262 fold change of the observed to random OR), Merlin (DNA, 0.301), ERVL-MaLR (LTR, 0.587), TcMar-Mariner (DNA, 0.725), TcMar-Tigger (DNA, 0.786), TcMar-Tc2 (DNA, 0.819), ERV1 (LTR, 0.902), L1 (LINE, 0.684), RTE-X (LINE, 0.765).

Families with lower number of members were tending to have lower fold change of the observed to random OR (Fig. 2a), whereas there was no apparent relationship between the fold change (level of enrichment) and average divergence within a family (Fig. 2b). Average divergence in all TEs by families was strongly correlated with average divergence in those TEs that are intersecting with TSS neighborhoods (Fig. 2c), with a single exception of a DNA family hAT-hAT19 which had 18 members in total, one of them appearing in the vicinity of gene TSS, and this single element had divergence 1.39-fold higher than the family average.

**Fig. 2.**
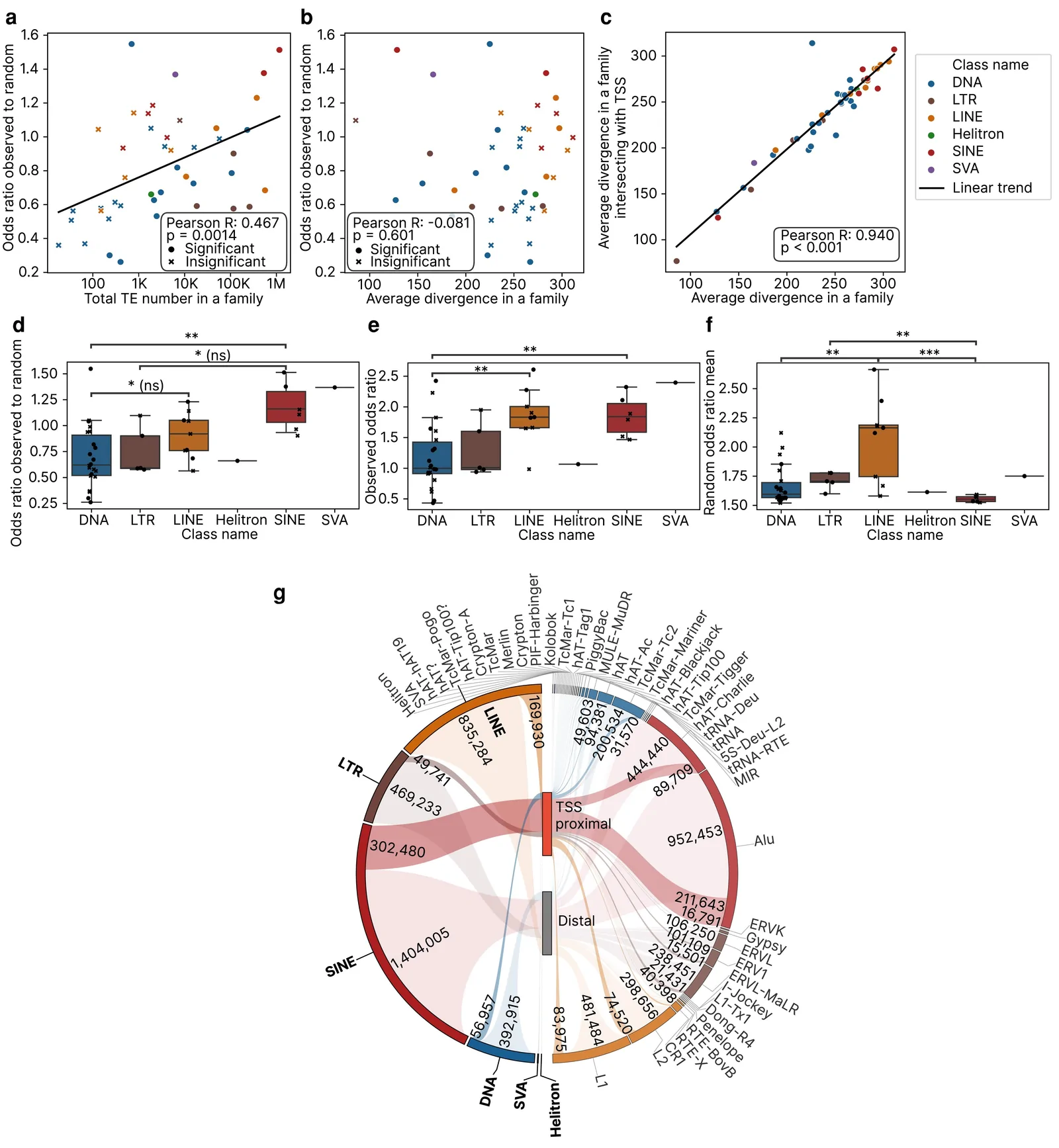
Further investigation of TE enrichment in the vicinity of TSS at the level of families. a) Scatter plot of observed to random OR by total number of TEs in a family. b) Scatter plot of observed to random OR by average divergence in a TE family. c) Scatter plot of average divergence of TSS neighborhood intersecting TEs by average divergence of all TEs in a family. d) Box plot of observed to random OR by TE families between TE classes. e) Box plot of observed OR by TE families between TE classes. f) Box plot of random OR by TE families between TE classes. g) Circular plot showing numbers of TE classes and families mapped in TSS proximal and distal regions. For all group comparisons in boxplots, significance is assessed by the Mann–Whitney test and FDR-corrected: ns, *P* > 0.05; *, 0.01 < *P* < 0.05; **, 0.001 < *P* < 0.01; ***, 0.0001 < *P* < 0.001; ****, *P* < 0.0001. Pearson correlation *P*-values on scatterplots are not corrected for multiple hypotheses. d to f) compare the 44 TE families grouped by class. The Helitron and SVA classes contain a single family each and are therefore drawn as individual points on the box plots. Mann–Whitney *U P*-values are Benjamini–Hochberg corrected across the testable class pairs; a bracket marked “* (ns)” denotes a pair significant on the raw *P*-value that does not survive the correction.

Additionally, DNA elements were significantly depleted within the TSS neighborhoods compared with SINEs (Fig. 2d, all the rest pairwise comparisons were nonsignificant after the FDR correction). The observed OR itself (without correction for random permutations) was significantly lower for DNA elements compared with both LINEs and SINEs (Fig. 2e), whereas the highest number of significant differences was found by the random background OR: between DNA elements and LINEs, between LINEs and SINEs and between LTRs and SINEs (Fig. 2f). These differences reflect the variable length of TEs by classes and highlight the importance of permutation-based random correction instead of the conventional Fisher exact test-based approaches. An integrative map of TEs that have been mapped to the TSS proximal regions and the distal ones at the level of classes and families is shown in Fig. 2g.

### Evolutionary age and length of TSS-proximal and distal TEs

To further understand evolutionary dynamics of TEs insertions near host genes, we compared distributions of divergence between all TEs and those that mapped on the TSS neighborhoods (Fig. 3a). The 2 distributions for all TEs were visually indistinguishable with characteristic bimodal shape observed earlier, although statistically significant differences were highlighted by Kolmogorov–Smirnov test. The same pattern was observed for individual TE classes (Fig. 3b), with 2 peaks found in SINEs and SVA elements; the difference is significant for 5 of the 6 classes, Helitrons being the exception (K–S *P* = 0.222, raw). Conversely, divergence of TEs that were intersecting with gene TSS neighborhoods, averaged over all TEs at the level of individual genes, followed a unimodal pattern both for all classes and for individual classes (Fig. 3c and d, respectively). For all classes, the peak divergence averaged among genes was 200 to 210 (Fig. 3c), whereas individual TEs without averaging over genes showed 2 peaks 110 to 120 and 270 to 280, regardless of intersecting with gene TSS (Fig. 3a). The same averaging effect was observed for SINEs: 2 peaks of 90 to 100 and 290 to 300 (Fig. 3b) converged to the single peak of 150 (Fig. 3d), indicating that there was no preference of integrating near any group of genes for SINE elements of different evolutionary age. For SVA elements the bimodal pattern was observed in the case of TSS averaging (Fig. 3d) due to the low number of TSS with SVA elements in their vicinity (1,140 TSS, 2.9%).

**Fig. 3.**
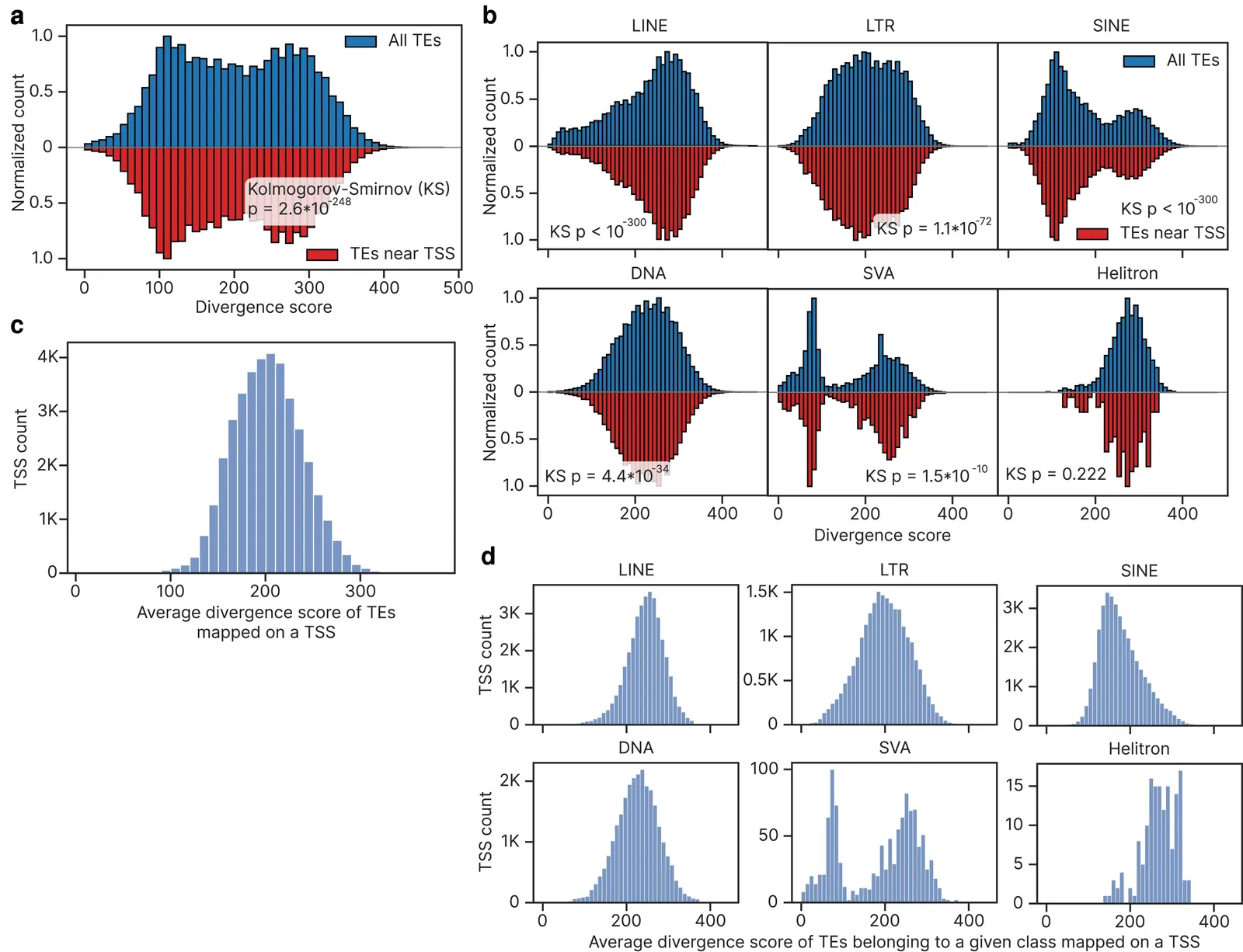
Evolutionary age comparison of all and TSS-proximal TEs in general and by classes. a) Ridge plot of all and TSS-proximal TEs by divergence score. b) Ridge plots of all and TSS-proximal TEs by divergence score at the level of individual classes. c) Average divergence score distribution of TSS-proximal TEs, averaged by TSS. d) Average divergence score distribution of TSS-proximal TEs at the level of individual classes. Distributions in (a) and (b) were compared by 2-sample Kolmogorov–Smirnov test; the *P*-values shown are raw, as each panel and subpanel is a single test.

Comparison of TE length distributions between all TEs and those that mapped on TSS neighborhoods resulted in the similar absence of difference between the 2 TE groups, either for all TEs or for their individual classes (Supplementary Fig. 1d and e, respectively).

We also compared divergence distributions between all and TSS-proximal TEs by individual families (Supplementary Fig. 2). Applying Mann–Whitney test with FDR correction resulted in 21 out of 44 families showing significant difference, albeit only 4 families showed magnitude of absolute difference above 10%: TcMar-Tc1, ERVK and tRNA SINEs demonstrated more than 10% higher average divergence for all insertions compared with the TSS-proximal ones (10.3%, 10.1%, 10.1%, respectively), and SVA elements had 10.6% higher divergence in insertions that overlapped with 10 kb TSS neighborhoods.

The analogous comparison by length resulted in 13 significantly different cases out of 44 TE families (Supplementary Fig. 3), with 4 families showing higher than 10% increase of average length near genes (hAT-Charlie with 10.7%, ERV1 with 17.6%, ERVL with 19.8%, TcMar with 53.0%), and a single family, hAT, showing 11.9% decrease of average length for TSS-proximal insertions. The TcMar family had 223 total and 16 TSS-proximal members in the T2T genome, reflecting likely random nature of such a high difference.

### Functional characterization of genes enriched by TE classes by count

To study whether TEs of different classes are nonrandomly accumulating near genes of certain functions, for each TE class we selected 5% of human genes whose TSS 10 kb neighborhoods contained highest number of TE elements of a given class. There were 1,436 genes for each of the major classes (LTR, LINE, SINE, DNA elements, Supplementary Fig. 4a, Supplementary File 2, sheet by_TE_group), genes having at least 5, 9, 18, and 5 elements of these classes have been taken, respectively (Supplementary Fig. 5a). Also, we extracted 962 genes with SVA elements and 130 genes with Helitrons (all genes with SVA elements and Helitrons were taken). Finally, we added 1,436 genes with highest (starting with 26 till 42 elements per TSS) and the lowest (starting with 4 till 0 elements per TSS) TEs count of all classes per TSS (Supplementary Fig. 5a). Because 28,738 unique genes had 38,704 TSS coordinates, and TE counts were measured on TSS whereas GO analysis is possible with unique gene names only, genes with maximum TE count could have TSS with lower TE count than the minimal thresholds, as indicated in Supplementary Fig. 5a. Since SINEs are the most numerous and enriched near genes TE class, they had the highest number of top genes which are shared with the top genes by all TEs (Supplementary Fig. 4a), and lowest number of top genes that are unique to SINEs (612 compared with 1,074 for DNA elements, 1,133 for LTRs and 1,031 for LINEs).

Each gene set was tested against the canonical GO Biological Process, Molecular Function and Cellular Component databases and significant terms were extracted (FDR threshold of 0.05 was applied, Supplementary File 3, sheet GO_TE_groups). No significant enrichments were found for DNA elements and Helitrons. The integrative network visualization of up to 10 of the most significant terms per remaining group (Fig. 4a; the complete network is Supplementary Fig. 6) showed that embryogenesis processes were the major ones among the 5% genes depleted with TEs of any class (with transcription being the second major category), whereas RNA splicing, DNA repair, telomere, apoptosis, IL-34 and ERBB signaling were among top 5% genes with highest TE count. Moreover, the 3 TE-depleted terms with the lowest FDR-corrected *P*-values in the entire set (chromatin, 2.3 × 10^−91^; DNA-binding transcription factor activity, RNA polymerase II-specific, 3.3 × 10^−83^; and sequence-specific double-stranded DNA binding, 2.7 × 10^−81^) all relate to transcriptional regulators. SINE-specific processes were the closest to the top TE count processes by the overlapping genes, sharing splicing and DNA repair as SINEs constitute the majority of TEs mapping in the vicinity of TSS (Fig. 2g). The rest of the classes with significant GO terms based on genes with the highest TE count led to olfactory receptor activity and negative regulation of fatty acid metabolism (LINEs), ion transport, ion transmembrane transport and synapse (LTRs), and termination of RNA polymerase II transcription (SVA elements). At the 0.05 FDR threshold the LINE class had 1 insignificant term, flavone metabolic process (FDR = 0.088), and the LTR class had 3 such terms: nucleotide binding (FDR = 0.076), glutamatergic synapse and positive regulation of lipopolysaccharide-mediated signaling (FDR = 0.086 for both). All 4 would be significant under a more relaxed FDR threshold of 0.1 and are rejected here; every term in this borderline, for every group, is listed in Supplementary File 3, sheet GO_borderline.

**Fig. 4.**
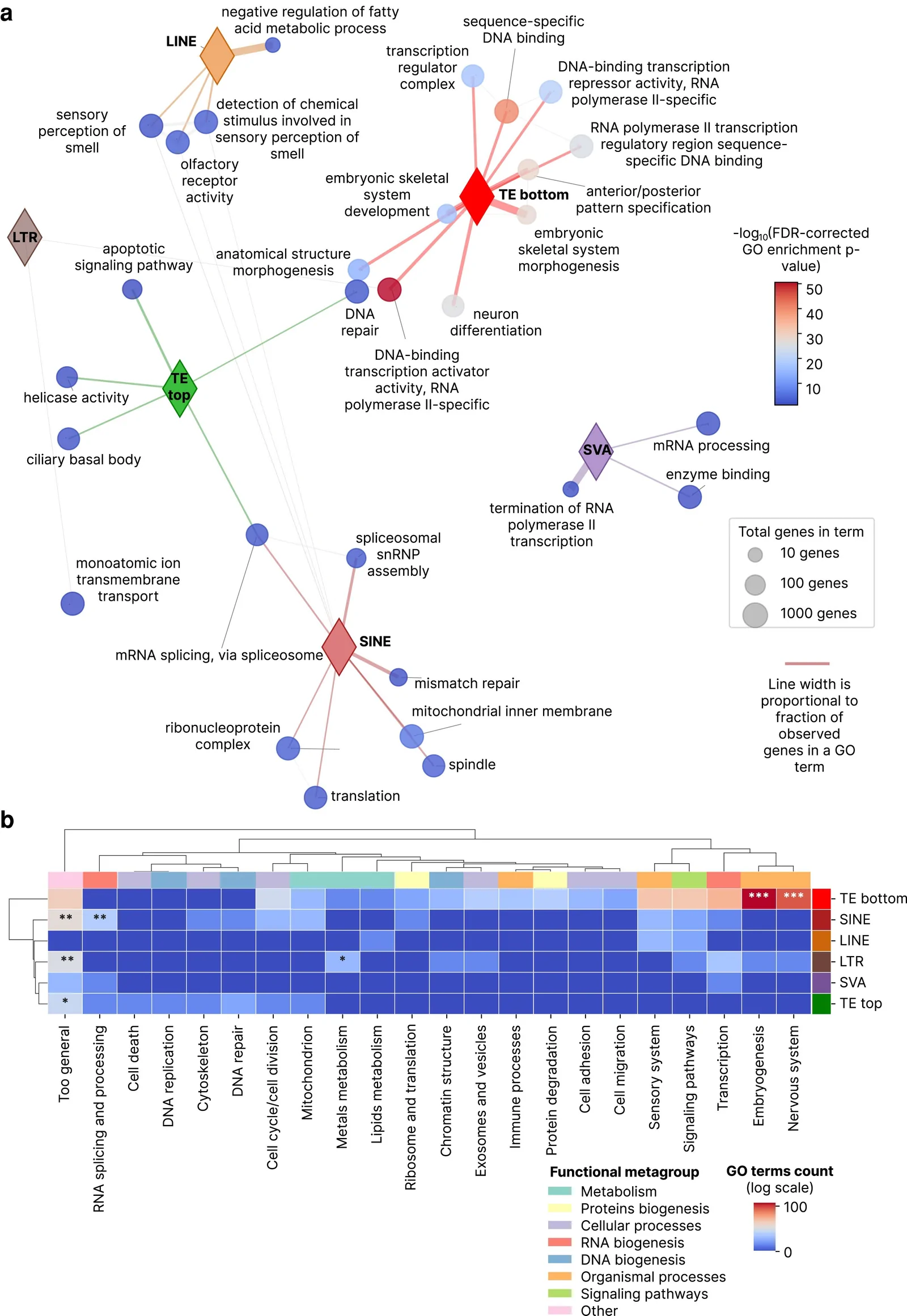
Functional analysis of genes whose TSS are enriched or depleted with TEs of different classes. a) Connection map of GO terms extracted for top 5% of TSS enriched with LINEs, SINEs, LTRs in their proximity and all TSS with SVA elements, as well as top 5% of TSS enriched or depleted with TEs of any class. Connection line width is proportional to fraction of shared genes, color of each node denotes a GO term enrichment *P*-value (FDR-corrected), node size shows number of genes in a GO term. Up to 10 terms by enrichment *P*-value were selected for each group, all with an FDR-corrected *P*-value below 0.05; the full network is Supplementary Fig. 6. GO terms with more than 500 genes were excluded to avoid overly general terms, and edges require a Jaccard index of at least 0.2 and at least 5 shared genes. b) Heatmap showing GO terms number by functional group and gene enrichment group (TE top, TE bottom, and TE classes). Stars indicate the FDR-corrected Fisher exact enrichment *P*-value of a given functional group in each gene enrichment group compared with the other enrichment groups.

The termination of RNA polymerase II transcription genes that had SVA elements in their vicinity were *POLR2A* (core catalytic subunit of RNA polymerase II) and genes *SSU72L1*, *SSU72L2*, *SSU72L3*, *SSU72L4*, and *SSU72L5*—protein phosphatases acting on RNA polymerase II C-terminal domain, whose 10 kb TSS neighborhoods are located in a 116 kb region of chromosome 11 (4,293,265–4,409,432) and have 3 copies of SVA B subfamily.

The GO terms were manually (with Gemini pro assistance) classified into 25 major groups, 22 of which are populated at this threshold, according to the previous studies (Igolkina et al. 2019; Nikitin et al. 2019a, b) and compared using Fisher exact test (Fig. 4b), testing enrichment of a given molecular process in a given TE group (classes, TE top and TE bottom) vs the same molecular process in the rest TE groups. While there was no systematic co-clustering of molecular process metagroups, RNA splicing and processing was specifically enriched in SINE-adjacent genes (FDR = 0.001) and metals metabolism was associated with LTRs (FDR = 0.010). Embryogenesis and nervous system processes genes were preferentially depleted with TE inserts of any class in their vicinity.

### Functional characterization of genes enriched by TE classes by TE evolutionary age

The next part of the analysis was dedicated to study associations of evolutionary young and the most ancient TEs by classes with the host genes. For each of the major TE families (LTR, LINE, SINE, DNA) and all TEs we calculated average divergence score of all TEs mapped in the 10 kb vicinity of each TSS (Fig. 3c and d). Then we selected top and bottom 5% of TSS with highest and lowest divergence, considering all TSS with TEs of a given group mapped. We extracted 1,425 genes of top and bottom divergence for all TEs, 1,396 genes for SINEs, 1,372 for LINEs, 1,015 for DNA elements, and 905 for LTR elements (Supplementary Fig. 4b, Supplementary File 2, sheet by_divergence). The upper limits for lowest divergence were 9.8% for LTR, 11.1% for SINE, 14.3% for DNA, and 16.8% for LINE, and the lower limits for highest divergence were 27.9% for SINE, 29.6% for LTR, 31.1% for DNA, and 31.4% for LINE (Supplementary Fig. 5b).

We visualized up to 10 GO terms for each TE-divergence group (Supplementary File 3, sheet GO_by_divergence), filtered by no more than 500 genes per term to avoid overly general classification (Fig. 5a; the complete network is Supplementary Fig. 7). The group of all TEs with low divergence was isolated from all other groups, returning the ribonucleoprotein complex, rRNA binding, the subcortical maternal complex and establishment of the Sertoli cell barrier. LTR elements of lowest divergence demonstrated a single oxidoreductase activity term acting on paired donors with reduced flavin as 1 donor (GO:0016712, FDR = 0.039). Groups of high divergence (all TEs, LINEs, SINEs, and LTRs) were co-clustered, and LTRs of high divergence shared olfactory receptor terms with LINEs of lowest divergence. LINEs of highest divergence were inserted near genes of potassium ion transport and the voltage-gated potassium channel complex, of protein kinase binding, of ventricular cardiac muscle cell differentiation and of the extracellular matrix structural constituent conferring tensile strength. SINEs and all TEs of highest divergence shared 98 GO terms connected with nervous system, especially ion channels and synapses, with smaller number of embryogenesis and transcription GO terms, specific for all TEs of highest divergence. The 3 most significant of these were postsynaptic membrane, chemical synaptic transmission and axon guidance (FDR corrected *P*-value 10^−16^ to 10^−10^), all 3 connected with all TEs of highest divergence and SINEs of highest divergence. LTR elements of highest divergence had also a specific term of calcium-dependent cell adhesion. LINE elements of lowest divergence demonstrated T, B, and NK cell activation and type I interferon receptor binding, as well as terpenoid metabolism. These immune system GO terms all shared a single core interferon gene set, which is characterized in detail in a separate Results subsection.

**Fig. 5.**
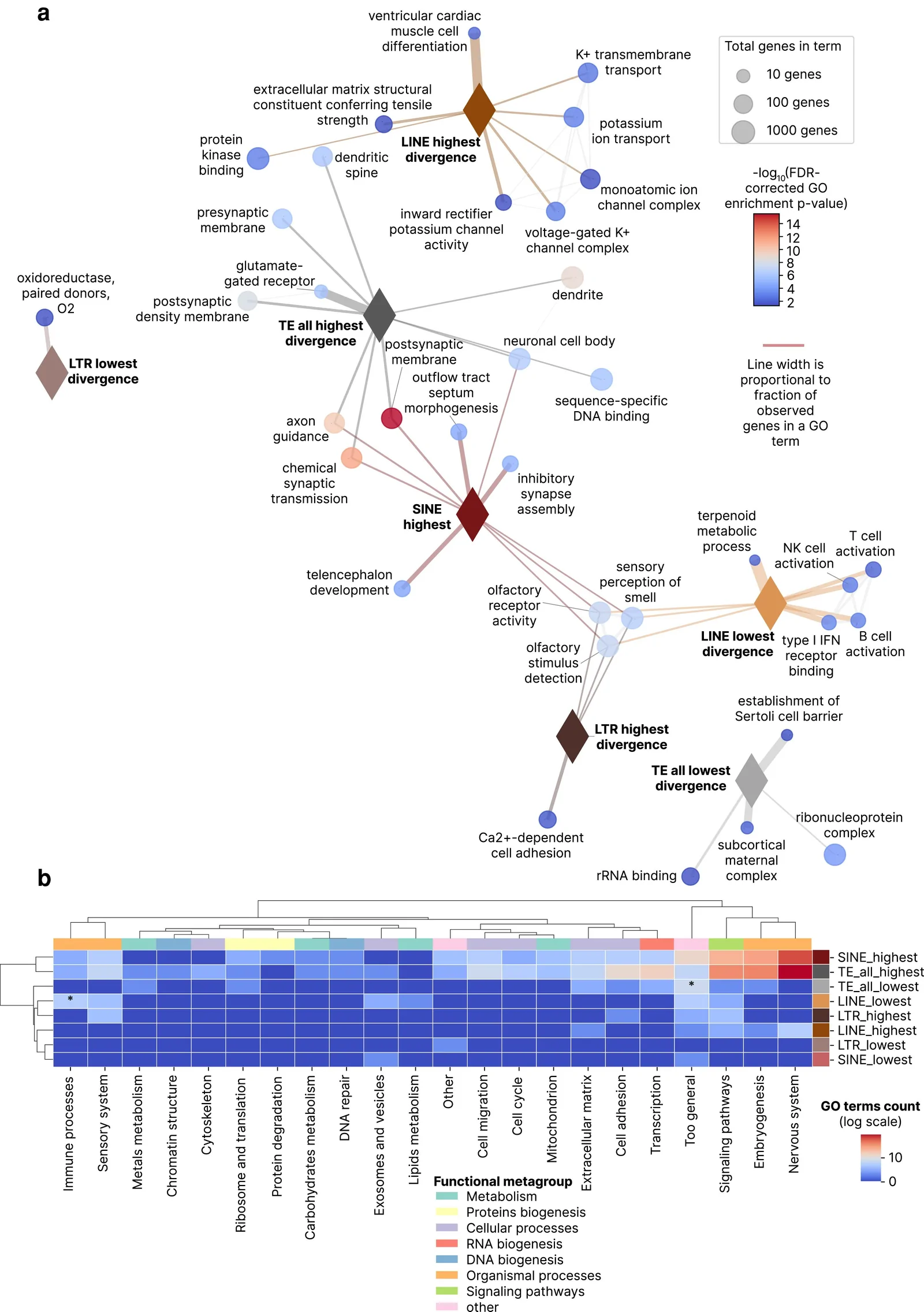
Functional analysis of genes whose TSS are enriched with TEs of different classes with highest and lowest divergence as an estimator of evolutionary age. a) Connection map of GO terms extracted for 5% of TSS with highest and lowest average divergence of LINEs, SINEs, LTRs, DNA elements, and all TEs. Connection line width is proportional to fraction of shared genes, color of each node denotes a GO term enrichment *P*-value (FDR-corrected), node size shows number of genes in a GO term. Up to 10 terms by enrichment *P*-value were selected for each group, all with an FDR-corrected *P*-value below 0.05; the full network is Supplementary Fig. 7. GO terms with more than 500 genes were excluded to avoid overly general terms, and edges require a Jaccard index of at least 0.2 and at least 5 shared genes. b) Heatmap showing GO terms number by functional group and gene enrichment group: highest and lowest divergence of LINEs, SINEs, LTRs, DNA elements, and all TEs. Stars indicate the FDR-corrected Fisher exact enrichment *P*-value of a given functional group in each gene enrichment group compared with the other enrichment groups.

Finally, DNA elements of highest and lowest divergence revealed no significant GO enrichments, and SINEs of lowest divergence only protein binding and extracellular exosome, 2 terms too general to be informative, reflecting potential absence of any functional preference of insertions, preferential mutational decay or negative possible selection, which is expected for DNA elements but surprising for young SINEs.

We then compared enrichment of different functional groups of GO terms in low or high divergence TEs of different classes (Fig. 5b). While SINEs and all TE elements showed high similarity between their processes (because SINEs are quantitatively predominant and enriched near genes), only immune processes were enriched for LINEs of lowest divergence groups, indicating possible molecular or evolutionary mechanism behind this pattern. No biologically reasonable co-clustering of TE groups or functional metagroups was found.

### Functional characterization of genes enriched by TE families by count

To analyze an enrichment of individual TE families near functional gene, we ordered TE families by number of TSS having at least one TE of a given family and then visualized the TE count distributions in the TSS vicinity (Supplementary Fig. 8). Thirty-four thousand nine hundred and seventy-seven (90.4%) of all TSS had at least one Alu copy in their 10 kb neighborhood (up to 29 copies per TSS), 30,695 (79.3%, up to 19 copies) had MIR elements, 30,376 (78.5%, up to 12 copies) had L2 elements, 30,184 (78.0%, again up to 19 copies) had L1 elements, and 18,731 (48.4%, up to 10 copies) had hAT-Charlie elements in their vicinity, whereas all the rest families were occupying less proximal regions.

For TE families that had more than 5% of all unique genes in their vicinity (1,436 and more) we selected 1,436 genes having highest count of elements of a given family in their vicinity, whereas for the less numerous and/or enriched families we took all the potentially affected genes (Supplementary File 2, sheet by_family). Network of these gene sets, and their overlapping's is shown in Supplementary Fig. 9a (no filtering of the graph edges by Jaccard index). Overlapping's were very weak as it can be expected for families with random and independent insertions, with highest Jaccard index of genes with 2 families being 8% (Supplementary Fig. 9a).

GO analysis for each family resulted in only 13 families having statistically significant (FDR-adjusted *P* < 0.05) terms (out of 44 total families): 3 LINE families (L1, L2, CR1), 5 LTR ones (ERVL, ERVK, ERV1, ERVL-MaLR, and Gypsy), 2 SINE families (Alu and MIR elements), SVA elements, and 2 DNA families (hAT-Charlie and hAT-Tip100) (Fig. 6a, Supplementary File 3, sheet GO_by_family). Two of the families with significant GO terms (hAT-Tip100 and L2) have only 1 term each, reflecting the likely random nature of these enrichments.

**Fig. 6.**
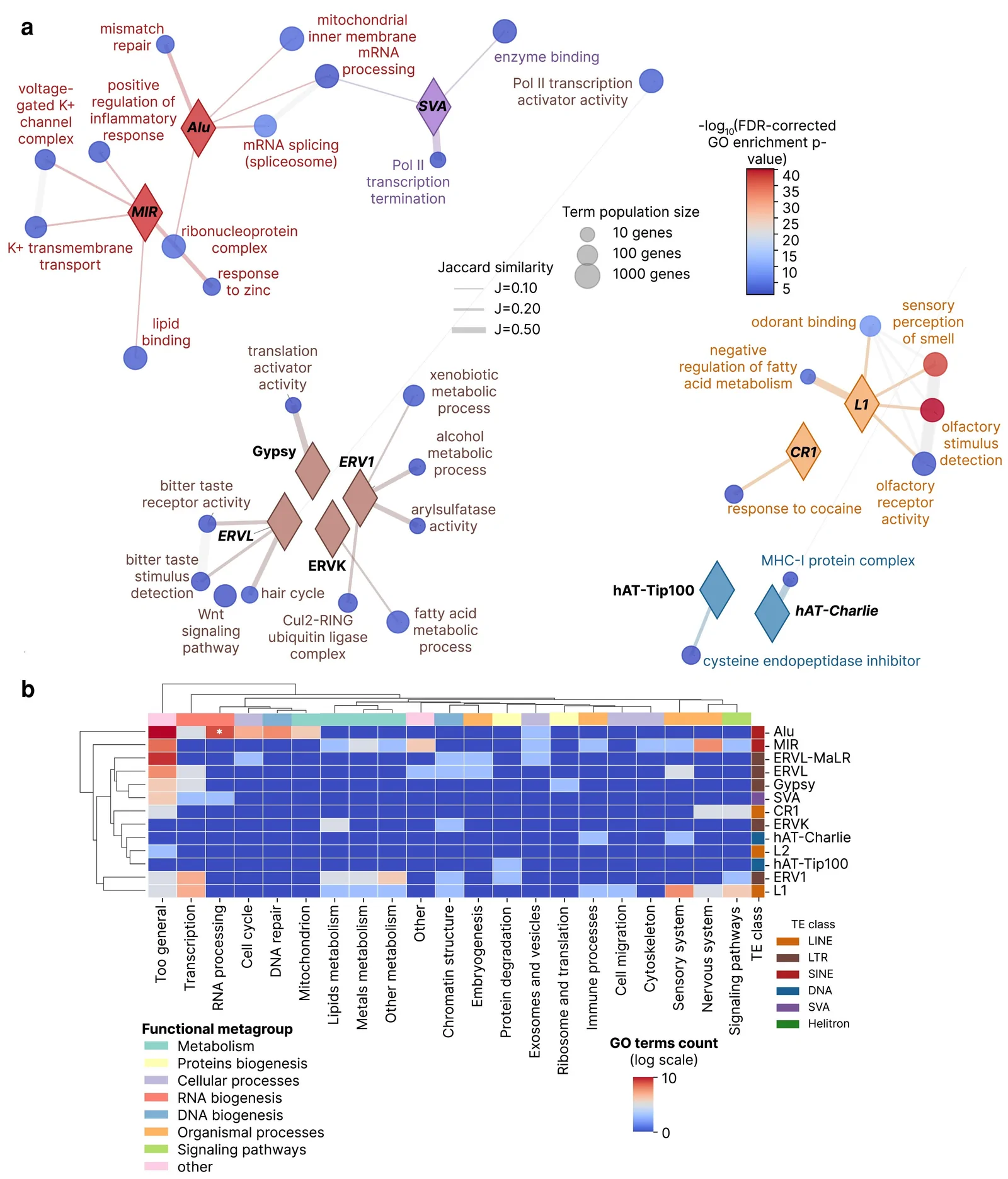
Functional analysis of genes with TSS enriched by TE inserts by family. a) Connection map of GO terms extracted for 5% of TSS with number of TE insertions by family. Connection line width is proportional to fraction of shared genes, color of each node denotes a GO term enrichment *P*-value (FDR-corrected), node size shows number of genes in a GO term. Up to 5 terms by enrichment *P*-value were selected for each family, all with an FDR-corrected *P*-value below 0.05; the full network is Supplementary Fig. 10. GO terms with more than 500 genes were excluded to avoid overly general terms, and edges require a Jaccard index of at least 0.2 and at least 5 shared genes. b) Heatmap showing GO terms number by functional group and gene enrichment group (TE family). Stars indicate the FDR-corrected Fisher exact enrichment *P*-value of a given functional group in each gene enrichment group compared with the other enrichment groups.

Visualization of up to 5 GO terms per family in Fig. 6a showed a high degree of functional distinction between processes by families (the full network is shown in Supplementary Fig. 10). L1 elements, as was previously shown for LINEs, were connected with olfactory receptors (FDR = 6.4 × 10^−41^), negative regulation of fatty acid metabolism (FDR = 4.0 × 10^−4^) and flavone metabolism (FDR = 0.031); L2 had noninformative protein binding; and CR1 elements had surprising connections with neurotransmitter-related processes, with synapse (FDR = 6.9 × 10^−4^) and response to cocaine (FDR = 0.027), while regulation of postsynaptic neurotransmitter receptor internalization, ventricular septum development and serine-type endopeptidase inhibitor activity were rejected (all FDR = 0.086). Flavone metabolic process (GO:0051552) is significant at the L1 family level but not at the LINE class level, where its FDR is 0.088.

LTR families demonstrated 33 unique GO terms compared with 22 in LTRs as a class (Supplementary File 3). ERVL elements were connected with bitter taste receptor activity (FDR = 0.023) and with hair cycle (FDR = 0.005), the keratin and intermediate filament terms falling outside the 0.05 threshold (FDR = 0.061 and 0.054), ERVK were associated with fatty acids metabolic process, ERV1 elements showed the most diverse set of GO terms (16 terms) and were inserted adjacent to genes of zinc and metal ion binding, transcriptional regulation, lipid and alcohol metabolism, xenobiotic metabolism, arylsulfatase activity, N-acylphosphatidylethanolamine metabolism, Wnt signaling and a Cul2-RING ubiquitin ligase complex. Gypsy elements were associated with translation activators, and ERVL-MaLR had weak connection with extracellular exosomes.

Alu and MIR insertions-adjacent GO terms differed (although both families enriched near genes, but Alu ones were significantly younger than the MIR ones), with Alu-specific ones connected with RNA splicing, DNA repair, meiosis, cell cycle and transcription initiation, which is very similar to the general SINEs patterns. Contrastingly, MIR elements were inserted in the vicinity of genes of voltage-gated potassium ion channels and potassium transport, protein-arginine deiminases, cellular response to zinc ion and intracellular zinc ion homeostasis, extracellular exosomes, positive regulation of the inflammatory response, cytoskeleton and sensory perception of sound. Ten GO terms of MIR elements fell between FDR 0.05 and 0.1 and were rejected as insignificant, among them cellular response to cadmium ion, detoxification of copper ion, the complement component C1q complex and neuromuscular junction (FDR = 0.078 for all 4), macrophage activation (FDR = 0.053) and actin binding, phosphatidylinositol-4,5-bisphosphate binding and negative regulation of response to cytokine stimulus (FDR = 0.062 for all 3); the complete set is listed in Supplementary File 3, sheet GO_borderline.

The only 2 DNA elements that showed nonrandom enrichment of adjacent genes GO terms were hAT-Tip100 and hAT-Charlie. hAT-Tip100 was associated with a single significant GO term of cysteine-type endopeptidase inhibitor activity (FDR = 0.040), hAT-Charlie with olfactory receptor activity (FDR = 0.011) and with MHC class I through a single term, MHC class I protein complex (FDR = 0.025); beta-2-microglobulin binding and antigen processing and presentation of peptide antigen via MHC class I both have FDR = 0.051 and were rejected by FDR.

Finally, SVA elements, as previously for the class-level analysis (Fig. 4a), related to termination of RNA polymerase II transcription.

We classified all the family-level GO terms into the main functional groups, following the same methodology as for the class-level analysis (Fig. 6b). Among 21 functional groups (including the “Too general” and “Other” groups), cell migration, cytoskeleton and ribosome biogenesis were found only once each, and cell adhesion was not represented. Protein ubiquitination and degradation and embryogenesis were the next least present groups, with 2 terms each. In contrast, too general terms, transcription and its regulation, and the nervous system and sensory system groups were the most frequent (41, 15, and 9 instances each, respectively). Among all 21 functional groups and 13 families with GO terms, there was no co-clustering by large-scale functional metagroups (Fig. 6b) and only the RNA splicing and processing group was significantly overrepresented, in Alu elements (FDR = 0.027), according to Fisher exact test of a process enrichment in any given family vs all the rest.

We also compared family-level GO terms with the respective class-level GO terms using a heatmap (Supplementary Fig. 9b), applying again the same FDR-corrected Fisher exact test for each functional group in each TE group, where TE group could be a TE family, TE class or top and bottom groups by all TEs. There were 24 functional groups extracted in this analysis (Supplementary File 3, sheet GO_by_family). Both RNA splicing and processing (FDR = 8.7 × 10^−4^) and DNA repair (FDR = 0.002) terms were enriched for Alu elements, whereas other (specific) terms were enriched for MIR TEs (FDR = 0.013). ERV1 elements had a statistically significantly overrepresented other metabolism group (FDR = 0.011), whereas ERVL-MaLR elements had general terms enriched (FDR = 0.006) and ERVK endogenous retroviruses remain co-localized with lipid metabolism genes (FDR = 0.029).

We than sought to elucidate the main factors determining the TE families functional impact measured as number of GO terms per family. We found no significant correlation with the enrichment level of TEs in the gene TSS vicinity (Fig. 7a, Pearson *R* = 0.167, raw *P* = 0.585), and the difference in GO term count between families with and without significant enrichment against the randomized background is not significant either (Fig. 7b, Mann–Whitney *U* raw *P* = 0.113; medians 12 and 2 for 11 and 2 families, respectively). As expected, the GO term number depended strongly on the total number of elements in a family (Fig. 7c, Pearson *R* = 0.645, raw *P* = 0.017). There was no significant relationship between GO term count and average family divergence (Fig. 7d, Pearson *R* = −0.283, raw *P* = 0.348; Fig. 7e, Mann––Whitney *U* raw *P* = 0.208), suggesting that TE families of different evolutionary ages can nonrandomly associate with defined functional groups of human genes. Finally, TE families with a nonzero number of GO terms had a significantly higher enrichment level (Fig. 7f, Mann–Whitney *U*, raw *P* = 0.029; median observed/random OR 1.04 against 0.73) and a significantly higher total copy number (Fig. 7g, raw *P* = 1.2 × 10^−6^).

**Fig. 7.**
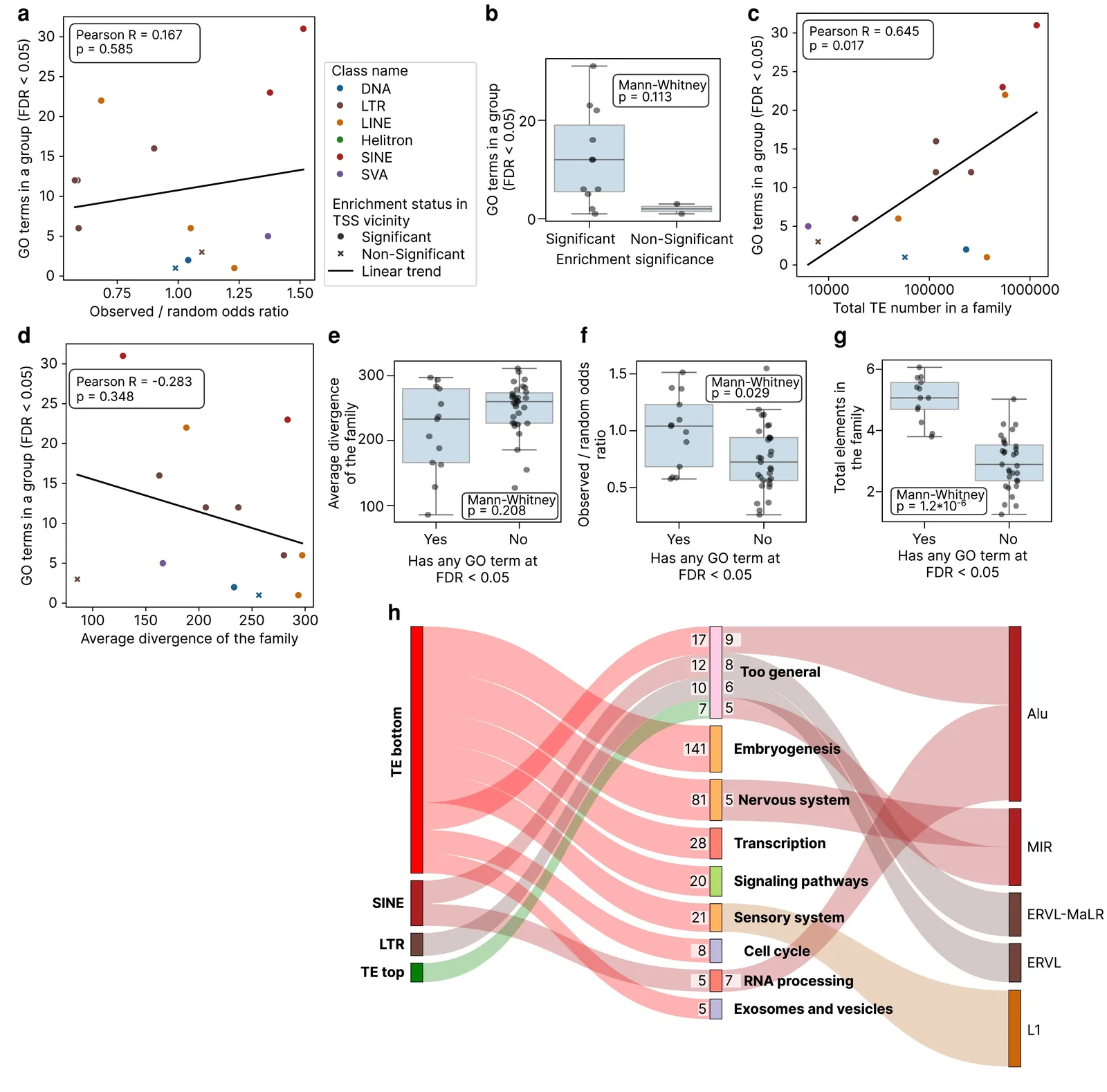
Analysis of main factors impacting TE functional associations with TSS at the level of TE families. a) Scatter plot of significant GO terms count by OR observed to random ratio. b) Box plot comparing TE families with significant and nonsignificant enrichment near TSS by significant GO terms count. c) Scatter plot of significant GO terms count by total TE number in a family. d) Scatter plot of significant GO terms count by average divergence in a family. e) Box plot comparing TE families with and without significant GO terms by average divergence. f) Box plot comparing TE families with and without significant GO terms by OR observed to random fold change. g) Box plot comparing TE families with and without significant GO terms by TE count. h) Sankey plot visualization of GO term groups found in TE classes (left) and families (right). Connecting ribbons were filtered by at least 5 GO terms. This filtering was applied to the visualization only. Pearson correlations in (a), (c), and (d) and Mann–Whitney *U* tests in (b), (e), (f), and (g) are single tests per panel and their *P*-values are raw.

The overall comparison of functional groups enrichment at the level of classes (with total TE top and bottom groups) with the enrichment at the level of families is depicted in a filtered view in Fig. 7h (connection ribbons in the Sankey plot are filtered by ≥5 GO terms per ribbon) and in an unfiltered representation in Supplementary Fig. 9c. Cell cycle and RNA processing could be affected by Alu insertions, whereas MIR elements were enriched in the vicinity of nervous system genes. The major groups such as embryogenesis, transcription, nervous system, sensory system and signaling pathways are present mainly in the TE bottom group and neither of TE classes nor families provide more enriched GO terms than the TE bottom group does (Supplementary Fig. 9b, these groups show an isolated clustering pattern). In contrast, the groups such as DNA repair, RNA processing, metals metabolism, other (specific) metabolism, cytoskeleton, cell death, DNA recombination and DNA replication do not appear in the TE bottom group and, despite the highly variable total GO terms count, can be considered as TE-enriched. All the rest functional groups showed inconsistent pattern.

### The interferon-alpha domain in depth analysis

The divergence-stratified analysis placed low-divergence LINE elements next to B, T, and NK cell activation genes (Fig. 5a). Those 3 GO terms share a single core gene set—*IFNA4*, *IFNA6*, *IFNA7*, *IFNA10*, *IFNA16*, *IFNA17*, *IFNA21*, and *IFNW1*—whose TSS neighborhoods all fall inside one 219,363 bp interval on chromosome 9 (chr9:21,150,692 to 21,370,055), the interferon-alpha domain. Because 1 locus carries 3 of the class-level associations, we analyzed it separately.

The domain contains 175 annotated TEs (Supplementary File 4, sheet IFNA_elements; Fig. 8a). L1 is its largest family with 77 elements, 44% of the whole domain TEs, followed by Alu (33), L2 (15), MIR (13), hAT-Charlie (11), ERV1 (10), and ERVL-MaLR (6); the remaining 10 elements belong to 7 further families. L1 density in the domain is 351 elements per Mb against 181.4 per Mb genome-wide, that is 565,459 L1 copies over 3,117 Mb, resulting in a 1.94-fold excess.

**Fig. 8.**
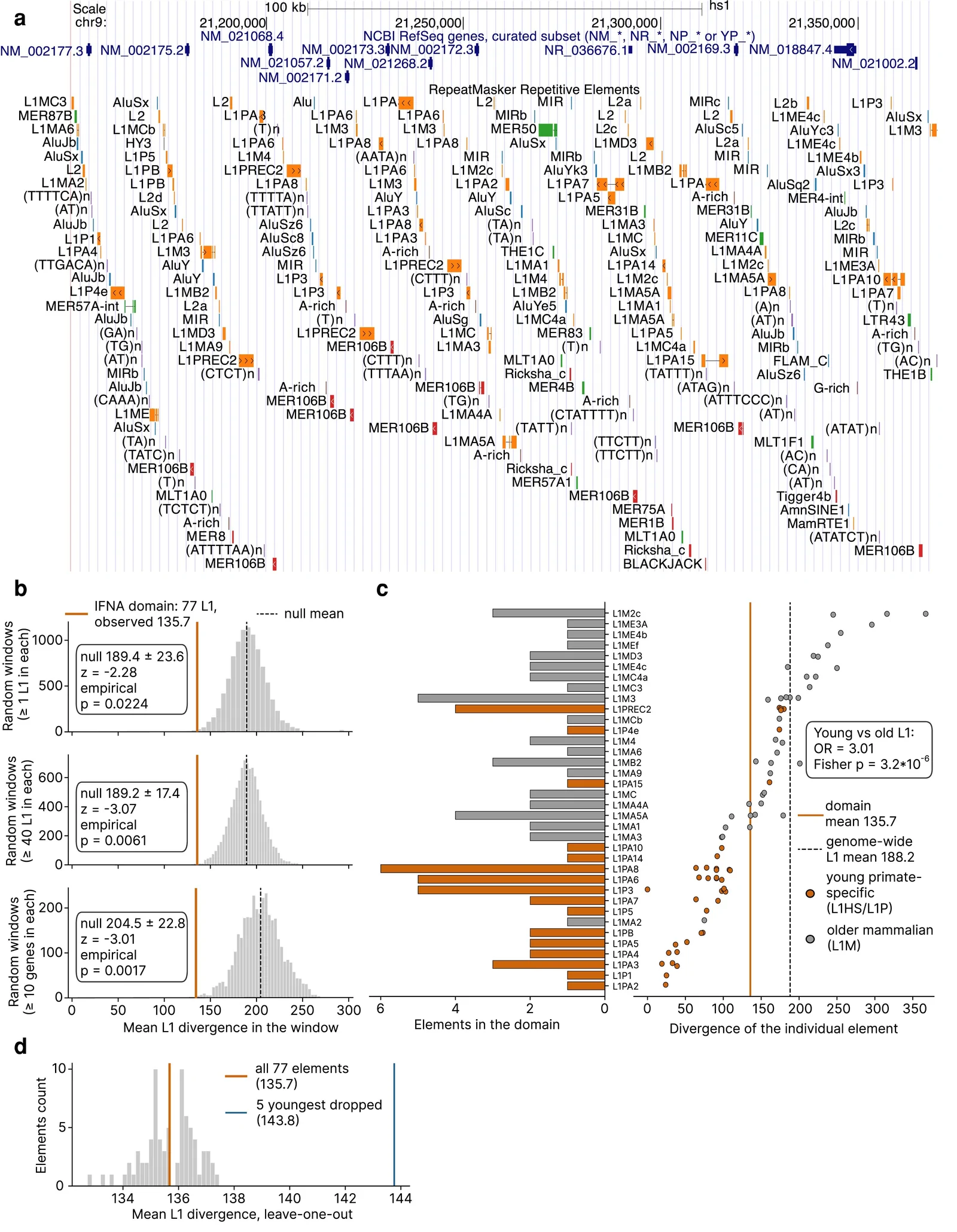
The interferon-alpha domain of chromosome 9 and the evolutionary age of its L1 elements. a) UCSC Genome Browser view of the 220 kb domain (chr9:21,150,692 to 21,370,055, T2T-CHM13v2.0/hs1) showing the curated NCBI RefSeq gene set and the RepeatMasker annotation. b) Null distributions of the mean L1 divergence in random 220 kb autosomal windows under 3 progressively better-matched null models: windows containing at least one L1 element (top, 10,000 windows), at least 40 L1 elements (middle, 10,000 windows, controlling for local L1 density) and at least 10 annotated genes and at least one L1 (bottom, 3,582 windows, controlling for gene density). The orange line marks the observed domain mean of 135.7 and the dashed line the null mean. The empirical *P*-values shown are 2-sided and raw, as each is a single test. c) L1 subfamily composition of the domain: the left bars give the number of elements in each of the 36 subfamilies and the right strip the divergence of every individual L1 element, both colored by whether the subfamily is young primate-specific (L1HS and L1P*) or older mammalian (L1M*). The dashed line marks the genome-wide L1 mean of 188.2 and the orange line the domain mean of 135.7. d) Mean L1 divergence recomputed with each of the 77 elements removed in turn, with the mean after removing the 5 youngest elements marked at 143.8.

The L1 elements of the domain are significantly younger than the genome-wide L1 population. Their mean divergence is 135.7 against 188.2 for all L1 elements, and the per-gene average divergence of the LINE elements around each of the 8 interferon TSS ranges from 95.0 for IFNA6 to 161.7 for IFNA21 (Supplementary File 1, sheet TSS_TE_intersections). The 77 elements span 36 distinct L1 subfamilies (Supplementary File 4, sheet IFNA_subfamily_composition; Fig. 8c), including the primate-specific L1PA2-L1PA8, L1PA10, L1PA14, L1PA15, L1P1, L1P3, L1P4e, L1P5, L1PB, and L1PREC2 alongside older mammalian L1M clades. Thirty-eight of the 77 elements belong to a young primate-specific clade, against 133,450 of the 545,659 genome-wide L1 copies assignable to either the young L1HS and L1P* or the older L1M* clade (Fisher exact OR 3.01, raw *P* = 3.2 × 10^−6^); HAL1 and X9_LINE elements, which belong to neither clade, were excluded from this test.

We then tested whether low mean divergence is a by-product of the high L1 density of the domain, of its gene density, or of a few extreme elements (Supplementary File 4, sheet IFNA_tests; Fig. 8b). Against 10,000 random 220 kb autosomal windows containing at least one L1, the observed mean of 135.7 lies 2.28 standard deviations below the null mean of 189.4 (2-sided empirical *P* = 0.022, raw). Restricting the null to windows with at least 40 L1 elements strengthens the result (10,000 windows, *z* = −3.07, raw *P* = 0.0061), and restricting it to gene-dense windows with at least 10 annotated genes and at least one L1 strengthens it further (3,582 windows, *z* = −3.01, raw *P* = 0.0017), having 1 of the 3,582 null windows being as extreme as the observation. Removing individual elements does not change the conclusion (Fig. 8d): excluding the single element annotated with divergence 0, implausible for its L1P3 clade and possibly a RepeatMasker artifact, raises the mean to 137.5, and removing the 5 youngest elements raises it to 143.8, both below every null mean.

We also checked whether the domain is a product of the new T2T assembly: there were no newly resolved regions relative to hg38 and the whole interval is alignable to the previous reference (Supplementary File 4, sheet assembly_bound), so this L1 cluster was visible before the T2T assembly. Taking these observations together, young L1 copies are concentrated in a locus of innate immune genes to a degree that 3 matched null models reject, which identifies the domain as a candidate for functional investigation. It should be noted that the does not establish that these elements regulate the interferon genes, which would require the expression and chromatin evidence that are out of scope of the present design.

### Comparison with the previous repeat-based gene categorization

To compare the current results with the landmark study by Lu et al. (2020), we tested the overlap between the current top 5% gene sets and the 3 repeat-enriched mouse gene categories in Lu et al. (2020). The categories by Lu et al. (2020) are defined on the mouse genome, so both sides were mapped to a common set of human genes with a mouse ortholog through MGI homology classes (Supplementary File 4, sheet prior_work_categories) (Baldarelli et al. 2024). The mapping was not uniform across their 3 categories: 2,042 of 2,439 low-complexity-repeat-enriched genes (83.7%) and 1,694 of 2,041 SINE-enriched genes (83.0%) carry an MGI ortholog, against 652 of 1,480 L1-enriched genes (44.1%).

The current Alu-enriched genes overlap the (Lu et al. 2020) SINE-enriched category 2.87-fold above chance, sharing 273 genes (OR 4.02, FDR = 4.4 × 10^−61^), which was the strongest association in the matrix despite the biological differences between human and mouse SINEs. The current SINE class behaved in the same way as Alu elements at the level of 2.71-fold (270 genes, OR 3.69, FDR = 6.1 × 10^−55^), as expected because Alu contributes most of the human SINE content. Our current L1-enriched genes overlapped the (Lu et al. 2020) L1-enriched category at the 2.22-fold level (49 genes, OR 2.44, FDR = 6.3 × 10^−7^), a weaker but consistent agreement on the second element class. The cross pairs of different classes were depleted as an expected negative control (Supplementary File 4, sheet prior_work_overlap_matrix).

It should be noted that the current MIR-enriched genes are depleted the SINE-enriched category from (Lu et al. 2020) (45 genes, 0.46-fold, FDR = 2.8 × 10^−9^), which can be explained by that dominance of the young rodent-specific B1 and B2 families among mouse SINEs. The 8 major comparisons between the current and the (Lu et al. 2020) TE enriched gene sets are collected in Table 3.

**Table 3.** Overlap between the current top-5% gene sets and the repeat-enriched mouse gene categories of (Lu et al. 2020).

Current gene set	Lu et al. category	Shared genes	Fold over expected	Adjusted Fisher *P*
Alu (family)	SINE-enriched	273	2.87	4.4 × 10^−61^
SINE (class)	SINE-enriched	270	2.71	6.1 × 10^−55^
L1 (family)	L1-enriched	49	2.22	6.3 × 10^−7^
L1 (family)	SINE-enriched	48	0.67	3.2 × 10^−3^
Alu (family)	L1-enriched	12	0.41	5.8 × 10^−4^
SINE (class)	L1-enriched	4	0.13	7.6 × 10^−9^
MIR (family)	SINE-enriched	45	0.46	2.8 × 10^−9^
MIR (family)	L1-enriched	12	0.40	5.0 × 10^−4^

Fold is the observed overlap divided by the overlap expected by chance in the shared human-mouse ortholog gene set; *P*-values are FDR adjusted across all 33 pairs of the full matrix (Supplementary File 4, sheet prior_work_overlap_matrix). The individual shared genes can be found in Supplementary File 4, sheet prior_work_shared_genes.

### Analysis sensitivity to the TSS window size and the gene-set cut-off

In order to test whether our analysis is vulnerable to the TSS window size, we repeated it at TSS neighborhoods of 5 and 20 kb, keeping the same TSS definition and a separate *N* = 500 permutation background for each window (Supplementary File 5, sheets window_classes and window_families). The observed-to-random OR of every class and family at all 3 windows is shown in Supplementary Fig. 11a: the ordering of TE groups was preserved, with Spearman *r* between window pairs from 0.828 to 1.000 and a label-shuffling permutation test rejecting chance concordance in all 6 pairs, the largest *P* being 0.0090 (Supplementary File 5, sheet window_concordance). The Bland–Altman comparison of the 44 families (Supplementary Fig. 11b) put the mean difference between windows at 0.027 to 0.084 observed-to-random OR units, so widening the window shifts the family distribution slightly upward (0.084 between 5 and 20 kb) without reordering it.

Significant enrichments changed for 11 of the 50 tested groups (Supplementary File 5, sheet window_flips): the Helitron class, and 10 families—ERVL, Gypsy, Helitron, I-Jockey, MULE-MuDR, PiggyBac, TcMar, TcMar-Mariner, hAT, and tRNA-Deu. These groups were small in total size and near the significance threshold for the 10 kb neighborhood (Fig. 1d).

We also overlapped gene sets across TE classes and windows (Supplementary Fig. 12; Supplementary File 5, sheets geneset_stability and rank_stability). The overlap coefficient of the top 5% gene sets between window pairs ranged from 0.285 to 0.931 (median 0.687) and every hypergeometric test against random expectation was significant, the weakest *P*-value was 3.5 × 10^−205^; the per-gene rankings agreed with Kendall rank correlation between 0.482 and 0.793 (median 0.695). The smallest gene-set overlap was that of the SVA class between 5 and 20 kb at 0.285, as expected for this small class with 6,274 elements. The lowest Kendall correlation was that of the DNA class over the same window pair at the level 0.482, reflecting more random distribution of DNA elements.

We also studied how widening the gene-set cut-off for the top and bottom from 5% to 10% impacted the results. Under the 10% cut-off, a median of 90.3% of the significant GO terms per group was preserved (Supplementary Fig. 11c; Supplementary File 5, sheet percentile_summary). In total, 144 terms were lost and 902 gained across all groups (Supplementary File 5, sheet percentile_terms). Of the 9 selected functional associations we reported in the main analysis, 8 remained significant at the 10% cut-off (Supplementary File 5, sheet headline_by_condition). The exception was the association of hAT-Charlie with the MHC class I protein complex, which remained significant only in the 5%, 10 kb window set (Supplementary Fig. 11d).

Repeating the GO analysis at every combination of the 3 windows and the 2 cut-offs returned a 6-cell grid of comparisons, which we built for the 3 GO analyses: classes by count, classes by divergence and families by count (Supplementary Fig. 13a; Supplementary File 5, sheet GO_grid_index). Widening the cut-off from 5% to 10% expectedly led to higher GO terms number in all TE groups and window sizes (Supplementary Fig. 13a). In contrast, widening the window did not result in a uniform trend: the number of significant terms decreased with window width for the TE class sets defined by element count, peaked at 10 kb for the divergence-stratified sets and increased for the families. Of the terms reported using the 10 kb neighborhood and 5% cut-off, the fraction still significant in another setting was 0.440 at the lowest level and 0.677 at the median level (Supplementary Fig. 13b; Supplementary File 5, sheet GO_grid_preservation), and the per-group term counts of every other setting correlated with the reported counts at a Spearman *r* of at least 0.614, label-shuffling permutation tests giving *P*-value below 0.022 (Supplementary File 5, sheet GO_grid_concordance). Three of the 9 reported conclusions were significant in all the 6 conditions (Supplementary Fig. 11d). Comparison of significant GO terms present by the 6 settings in all groups investigated in this work (Supplementary Fig. 13c) revealed that under the 20 kb TSS neighborhood the otherwise randomly inserted TE families (Dong-R4, Penelope, RTE-BovB, TcMar-Tigger) attained significant GO terms at the proximal genes.

## Discussion

In the present paper, we performed an integrated analysis of human TEs co-insertion with human genes functional groups, based on the recent T2T genome assembly at the level of TE classes and families. Currently dozens of research groups analyzed functional impact of human TEs on the host genes (Bourque et al. 2008; Sundaram et al. 2014; Ito et al. 2017; Nikitin et al. 2019c; Lu et al. 2020; Correa et al. 2021), all with different methodology and in different cellular and epigenetics contexts, setting the stage for large-scale comparing and review papers to understand the available evidence and put it in a single cohesive network. The current article is meant to establish a baseline for these studies by co-mapping proximity analysis, since most epigenetics-based studies rely on the same proximity principle. Aiming to derive such a proximity-based baseline, we excluded any epigenetics or transcriptomics analysis out of scope of this work. The availability of the complete human genome assembly (Nurk et al. 2022) allows to do such an analysis with an unprecedented depth and precision, compared with the currently available literature.

### Main findings

In this research analysis, we report the 3 major results blocks. First, TE proximity to human genes is close to universal: 99.11% of the 38,704 unique TSS have at least one annotated TE within 10 kb, and only 343 do not. Compared with earlier estimates that roughly a quarter of human promoters are TE-derived (Sundaram et al. 2014), the shift from 25% “TE-derived” to 99.11% “TE-proximal” marks the difference between a TE supplying the start site and a TE merely being available as an auxiliary regulator, insulator or chromatin modifier.

Second, TE enrichment near TSS is modest and class-specific under random permutation correction: SINEs and SVA elements are enriched (1.468- and 1.368-fold over random expectation) while LINEs, LTRs, and DNA elements are depleted (0.877, 0.667, and 0.938).

Third, the functional associations of TE-proximal genes are separated by TE group. Genes with the lowest TE counts are dominated by embryogenesis, transcription and nervous-system terms, consistent with core pathways being protected from TE-borne regulatory noise; genes with the highest TE counts are enriched for RNA splicing, DNA repair, telomere maintenance and apoptotic signaling. Individual groups confer an additional level of specificity: SVA elements are concentrated near genes for termination of RNA polymerase II transcription, Alu near RNA splicing and DNA repair, MIR near zinc homeostasis and voltage-gated potassium channels, L1 near olfactory receptors and fatty acid metabolism, LTR families near ion transport genes. The interferon-alpha domain on chromosome 9 is shown as a single locus with a strong signal: 77 of its 175 TEs are L1 copies spanning 36 subfamilies, at a mean divergence of 135.7 against 188.2 genome-wide, confirmed as significant difference against the background windows controlled for L1 count and for gene density (Fig. 8).

### Comparison with prior research

According to the available evidence, the degree of enrichment or deficiency of TE groups near human genes can be determined by multiple factors (Fig. 9): selection pressure governed by effective population size (Rishishwar et al. 2017; Marino et al. 2024), TE insertion machinery affinity (Cost et al. 2001; Lavie et al. 2004), neutral evolutionary mechanisms of complexity and interdependency growth, such as constructive neutral evolution (Pirogov et al. 2019; Muñoz-Gómez et al. 2021; Catherall-Ostler and Dixit 2025), and spatiotemporal dynamics of TE activation during the germline development (Dietmann et al. 2020; Maupetit-Mehouas and Vaury 2020).

**Fig. 9.**
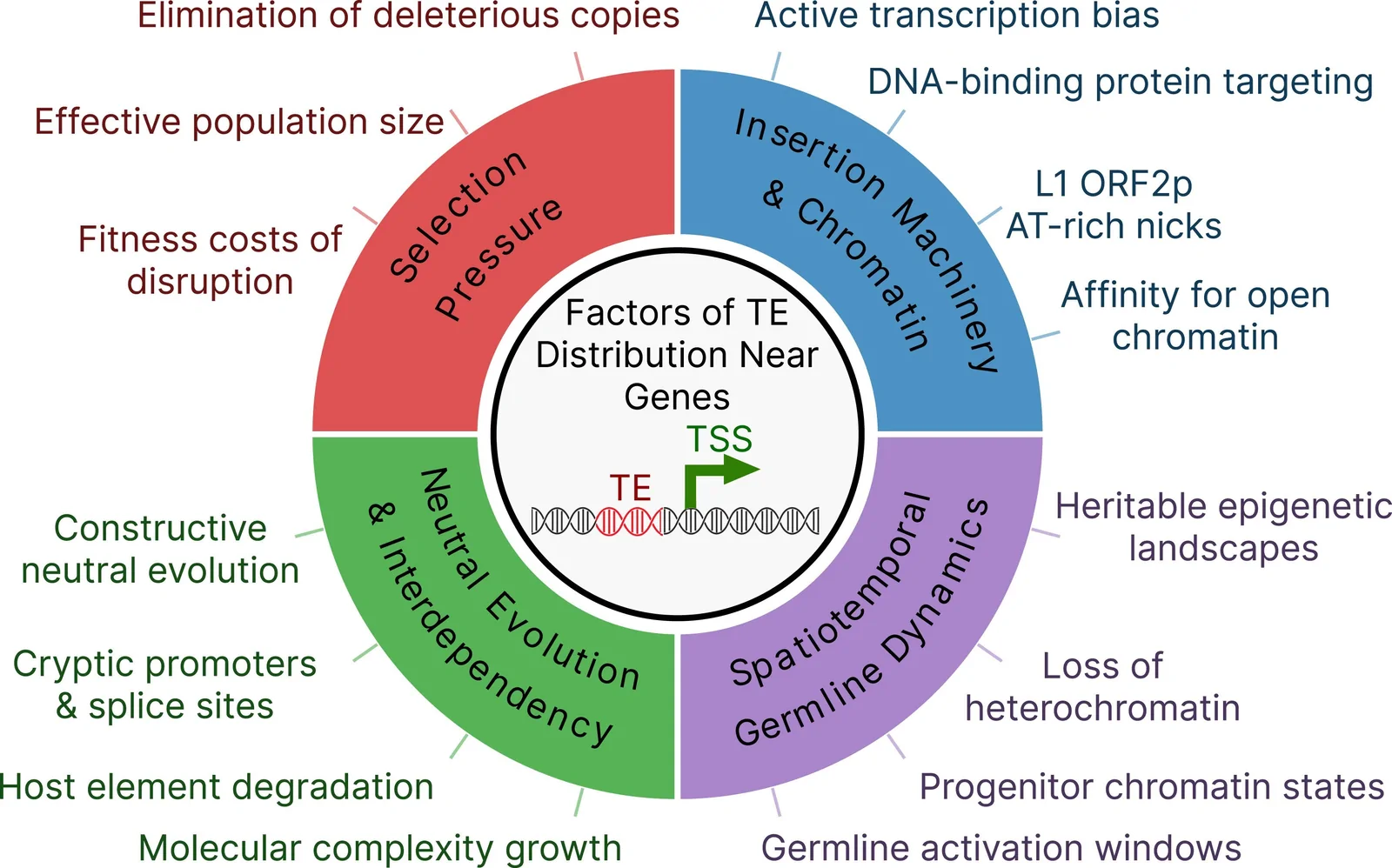
Schematic representation of mechanisms impacting distribution of TEs with respect to TSS.

Our current analysis extends this understanding toward functional groups of genes that could be impacted by their promoter-proximal TE insertions, and our findings are deeply connected with evolution of these functional groups. For example, we show that L1 elements are specifically enriched near olfactory receptor genes, which could allow rapid diversification of sensory perception of smell sensing through TE-mediated rearrangements, leading to genes duplication and subfunctionalization or gene losses (Beck et al. 2011; Redaelli et al. 2024). Moreover, Alu elements were early shown to profoundly impact RNA splicing by introducing new splice sites (exonization), acting as alternative exon sinks, and forming double-stranded structures that alter exon inclusion (Payer et al. 2018). We now show that Alu copies are significantly enriched near the host genes related to splicing, which is a completely orthogonal mean of Alu-mediated impact on splicing.

At the level of TE classes, the previous landmark study by (Lu et al. 2020) utilized 20 and 2 kb window to show nonrandom association of SINEs with housekeeping genes having “broad” promoters with multiple TSS and LINEs with tissue-specific ones having “sharp” promoters with single TSS (Haberle and Stark 2018). Precisely, in that study SINE-enriched genes were associated with ribosome, translation, RNA processing, nucleolus and protein transport, whereas LINE elements were enriched in genes of olfactory receptors, retinol metabolism, epoxygenase P450 pathway and immunoglobulin domains. Our analysis partly confirms these findings (Fig. 4a and b). Similarly, we show that RNA processing, ribosome and translation, transcription and general pathways are enriched in SINEs. But such highly cell-type specific processes as embryogenesis and sensory system were also present as a minor part of SINE elements. For LINEs we confirmed olfactory receptors, whereas lipid metabolism contained different terms: flavone (Hostetler et al. 2017) metabolism and negative regulation of fatty acids metabolism. Also, for L1 elements we report TF activity and calcium ion binding. These partial differences can be explained by the methodological differences: (Lu et al. 2020) profiled TE abundance separately in Promoter, Intron, Downstream, 5′ UTR, CDS and 3′ UTR regions of each gene, then performed quantile normalization and hierarchical clustering, analyzing gene groups arising from this clustering, and did the analysis on mouse genes. As we derived, only 270 of our current SINE-enriched genes overlap with the 2,041 genes reported as SINE enriched by (Lu et al. 2020). Such a weak intersection, albeit significantly above the random background, can explain the differences in results between the 2 analyses.

In general, the functional characterization of genes based on their proximal TE content in our analysis reveals a clear segregation of biological processes. This “Ring of Power” functional network illustrates how the host genome manages the risks (deleterious mutations) and rewards (power of innovation) of TE proximity (Fig. 10). The extensive discussion of the observed trends in the context of the public evidence can be found in Supplementary File 6.

**Fig. 10.**
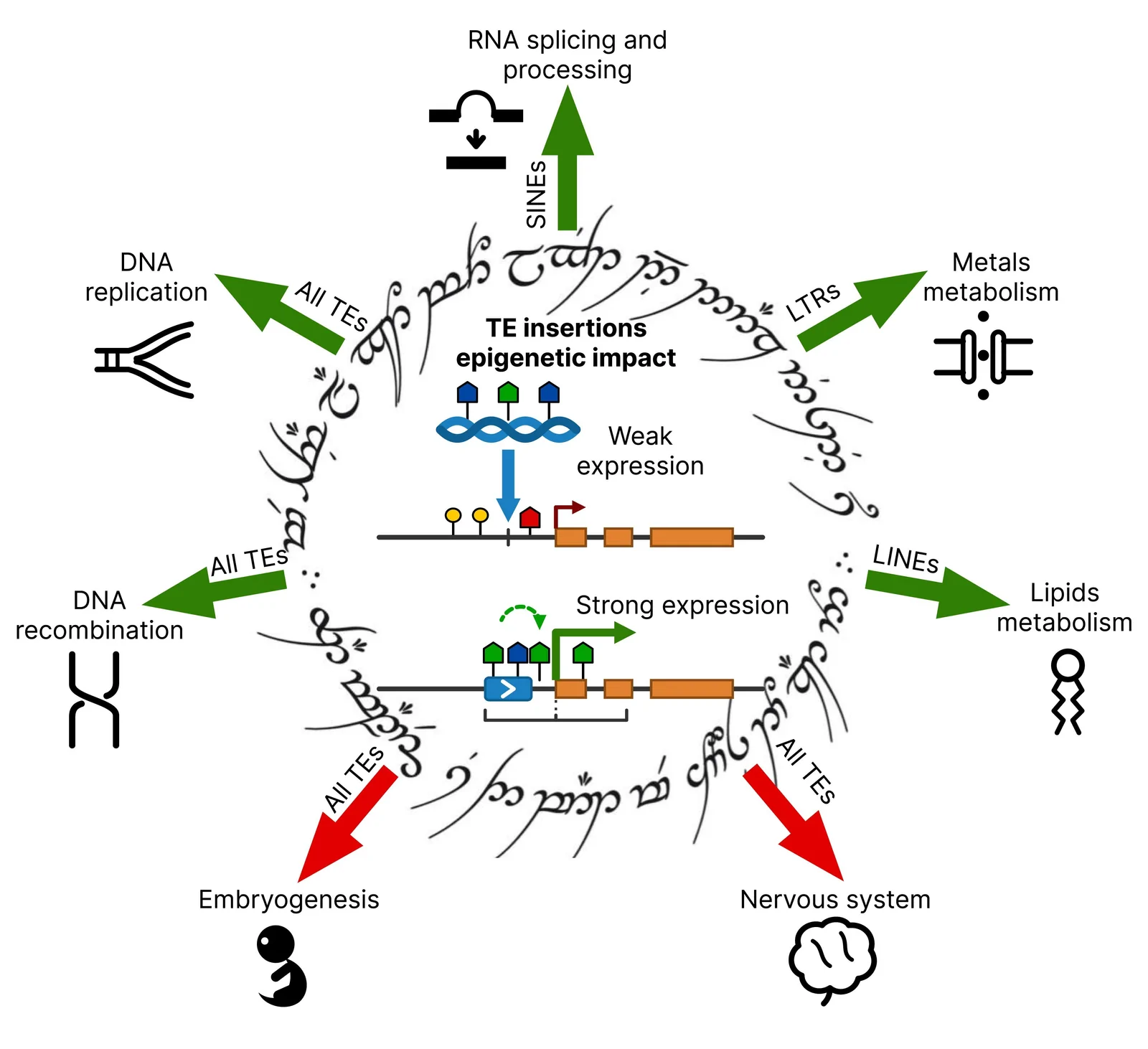
Schematic representation of molecular processes groups that were found as significantly enriched (metals and lipids metabolism, RNA processing, DNA replication and recombination) or depleted (nervous system and embryogenesis) with individual TE classes or all TEs.

### Connection of the TE enrichment results with cancer

The recent evidence points out high transpositional activity in cancer, with more than 500 L1 insertions per tumor in bladder cancer according to a recent preprint (Pribus et al. 2025). Moreover, large-scale TE de-repression is an emerging hallmark of cancer. Our current work defines the scope of human molecular processes that can be aberrantly activated in cancer by distinct TE classes and families and potentially targeted by anticancer therapy (Gudkov et al. 2022).

The contemporary anticancer treatment heavily relies on the single molecule-based biomarkers (Jovčevska et al. 2019; Sorokin et al. 2022) while there is an increasing need for development of the compound ones, reflecting tumor genome and microenvironment distinct states (Sorokin et al. 2021; Vladimirova et al. 2021; Fiore et al. 2023) as well as plasma exosomes impact (Shtam et al. 2018a, b). Among the promising ones is gene signatures approach which measures activation of multiple genes simultaneously (Sorokin et al. 2020; Adamyan et al. 2021; Yudina et al. 2025). Genes identified as enriched by certain TE classes and families can be used to compose gene expression signatures and measured in the publicly available cancer cohorts such as TCGA (Liu et al. 2024). In turn, such an evolutionary approach could improve cancer treatments outcomes as cancer is primarily an evolutionary disease arising from the genomic conflict of interests (Ottaiano et al. 2026).

### Limitations of the current proximity analysis

The most important limitation of our analysis is that it cannot show whether a certain TE regulates a given gene, since no expression, chromatin or perturbation data enter the analysis. Therefore, the putative arms-race in the interferon-alpha domain is only a hypothesis requiring further functional studies. Importantly, we designed this purely genomic proximity analysis in such a way to be a baseline for the already published epigenomic and transcriptomic proximity-based studies (Bourque et al. 2008; Sundaram et al. 2014; Ito et al. 2017; Lu et al. 2020; Correa et al. 2021; Nikitin 2025) as well as the future studies.

We also see limitations of a smaller scale. First, the polymorphic regions with structural variants were not included in the T2T-CHM13v2.0 single haploid genome, and incorporation of human pangenome reference could address this limitation since TE are enriched in the polymorphic regions (Liao et al. 2023). Second, GO annotation is biased toward well-studied gene families, which inflates the apparent specificity of terms such as olfactory receptor activity where large paralogous families are annotated together (Haynes et al. 2018). Third, genes differ systematically in their number of annotated TSS, and because TEs were counted per TSS a TE in 2 windows of the same gene was counted twice, so genes with many isoforms are over-weighted; the effect is a known property of the current design and the updated pipeline with selection of only one isoform per gene could be tested in future.

### Further research directions and hypotheses to be tested

The evolutionary young L1 elements in the interferon-alpha domain is the most promising further research direction highlighted in the current work. If the young L1 copies in this 220 kb region contribute to interferon gene regulation, CRISPR interference targeted at individual L1PA and L1P insertions in the domain (Price et al. 2021) should alter the induction kinetics or the amplitude of IFNA transcription under interferon or double-stranded RNA stimulation, with an effect restricted to genes whose TSS are located within the same window. The L1 elements could equally be passive passengers in the locus, which is a null hypothesis here.

Second, the SSU72L phosphatase cluster on chromosome 11 carries 3 SVA_B copies within a 116 kb region, resulting in association with genes for termination of RNA polymerase II transcription. Deleting the SVA_B insertions at that cluster could change read-through or polyadenylation site usage at the adjacent phosphatase genes if the elements are functionally recruited.

Third, and most generally, the proximity values reported here could serve as a baseline that published mark-based enrichments can be conditioned on (Nikitin 2026a). Any result of TE families being enriched for a chromatin mark near genes of a given function could be re-expressed as an enrichment over the positional expectation for that family and that gene class, which the current map supplies for 6 classes and 44 families genome wide. We anticipate that a measurable fraction of published epigenomic mark-based associations will be normalized and appear insignificant this way, improving sensitivity of the evolutionary arms race processes detection.

## Supplementary Material

jkag250_Supplementary_Data

## Data Availability

All code used for the proximity mapping, statistical analysis and GO functional networking is available in the GitHub repository https://github.com/Nikit357/T2T_transposons_genes. The TE annotation, the TSS 10 kb windows, the gene sets used for GO analysis and the interferon-alpha domain are also available as a UCSC Genome Browser track hub on the hs1 (T2T-CHM13v2.0) assembly, colored by TE class to match the figures. Hub URL: https://nikit357.github.io/T2T_transposons_genes/. The domain discussed above opens directly at https://genome.ucsc.edu/cgi-bin/hgTracks?db=hs1&hubUrl=https://nikit357.github.io/T2T_transposons_genes/trackhub/hub.txt&position=chr9:21150692-21370055. Transposable element annotations were obtained from the UCSC RepeatMasker track for T2T-CHM13v2.0 (hs1) and gene annotations from NCBI RefSeq All (hs1), both via the UCSC Table Browser. An archival snapshot of the repository at the time of publication is deposited in Zenodo under DOI 10.5281/zenodo.21980023. Supplementary Figs. and Files are available on GSA FigShare: https://doi.org/10.25387/g3.33286680. Supplemental material available at G3 online.
