# Supplementary material for: Telomere-to-telomere co-mapping of transposable elements and human genes identifies a cluster of young L1 elements in the interferon-alpha domain with potential evolutionary arms-race
Source: G3 (Bethesda). 2026 Sep 23;16(11):jkag250. doi: 10.1093/g3journal/jkag250 (PMC13732488; doi:10.1093/g3journal/jkag250)
Supplement: Supplementary file 1 [file jkag250_supplementary_data.zip › Supplemental_Material_Legends_G3-2026-406828.docx]

# **Supplementary material**

**Supplementary Figure 1.** Technical controls on the permutation background and on element length. (A) Running mean of the random odds ratio for each TE class as permutations accumulate, as a fraction of its value at N = 500; the shaded band is +/- 1%. (B) Running standard deviation of the same quantity, on the same scale. (C) Drift of the running mean for all 44 families, expressed in units of the final standard deviation, so that families with different absolute odds ratios are comparable. (D, E) Ridge plots for length distribution comparison between all TEs (blue) and those mapped on TSS neighborhoods (red), (D) for all classes together and (E) for individual classes; distributions were compared by two-sample Kolmogorov-Smirnov test. The p-values shown are raw, as each panel is a single test; panel (E) reports one test per class, six in total, of which five are significant.

**Supplementary Figure 2. Ridge plots for divergence distribution comparison between all (pale colors) and TSS neighborhoods mapped TEs (full colors) for individual families. The distributions are colored by TE class. For all comparisons significance is assessed by the Mann–Whitney test and FDR-corrected: ns, p > 0.05; *, 0.01 < p < 0.05; **, 0.001 < p < 0.01; ***, 0.0001 < p < 0.001; ****, p < 0.0001.**

**Supplementary Figure 3. Ridge plots for length distribution comparison between all (pale colors) and TSS neighborhoods mapped TEs (full colors) for individual families. For each family, top and bottom 2.5% of points were clipped to ensure visual capture of the differences. The distributions are colored by TE class. For all comparisons significance is assessed by the Mann–Whitney test and FDR-corrected: ns, p > 0.05; *, 0.01 < p < 0.05; **, 0.001 < p < 0.01; ***, 0.0001 < p < 0.001; ****, p < 0.0001.**

**Supplementary Figure 4. Supervenn plots for gene set intersections. Each gene set is divided into gene groups that differ by gene sets sharing genes from a given gene group. For each panel, colored rectangles in the main plot show these gene groups, top grey bar plot indicates number of set that each gene group shares, lower number show gene count in each gene group. Right side bar plot visualizes gene counts per gene set. (A) Intersections of gene sets: enriched with all TEs (TE top), depleted with all TEs (TE bottom) and enriched by each of TE classes. (B) Intersections of gene sets with all TEs, LINEs, LTRs, DNA elements and SINEs with highest and lowest divergence.**

**Supplementary Figure 5. TSS distributions by count (A) and divergence (B) of mapped TEs, with red bars denoting those TSS whose genes were taken into the Gene Ontology analysis. Pale blue histograms show all TEs. On both panels distributions are shown for individual classes and all TEs. For the divergence panel (B) both highest and lowest divergence groups are shown.**

**Supplementary Figure 6. Extended connection map of GO terms enriched in the gene sets defined by the number of TEs of each class near the TSS, of which Figure 4A is the simplified view. Up to 30 terms per group are shown against ten in Figure 4A, with edges drawn at a Jaccard index of at least 0.1, with no shared-gene floor, and terms of at most 1,000 annotated human genes retained. Node color denotes the FDR-corrected enrichment p-value and node size the number of genes in the term; group nodes are diamonds colored by TE class.**

**Supplementary Figure 7. Extended connection map of GO terms for the divergence-stratified gene sets, of which Figure 5A is the simplified view. Groups are each TE class split into its highest- and lowest-divergence tails, together with the same split for all TEs combined. Up to 30 terms per group are shown, with the same edge and term-size filters as Supplementary Figure 6.**

**Supplementary Figure 8. Log-scaled distributions of TSS by TE number mapped on their 10 kb neighborhood. The distributions are plotted for individual TE families and colored by their class. Numbers on the right show TSS counts and percentages with non-zero TEs in their vicinity.**

**Supplementary Figure 9. Genes and molecular processes enriched with TE of distinct families in their 10 kb vicinity. (A) Intersections map of genes sets enriched with TEs by family. Circle size is log-proportional to number of genes in a set. Circles are colored by TE class, and color intensity denotes OR observed to random. TE families with significant enrichment near TSS are marked as bold. Connection line width is proportional to Jaccard index between a two gene sets. (B) Cluster map of GO terms number by functional groups and TE groups: TE top, TE bottom, the four classes with significant GO terms (LINEs, SINEs, SVA elements and LTRs) and TE families with significant GO terms. Colors on the cluster map side annotation denote TE groups and functional metagroups. For all clustermap cells significance is assessed by the Fisher exact test of a given TE group and a given functional group against the same functional group in the rest TE groups and FDR-corrected: *, 0.01 < p < 0.05; **, 0.001 < p < 0.01; ***, 0.0001 < p < 0.001; ****, p < 0.0001. (C) Sankey plot of GO terms count comparison by groups between the large-scale TE groups (the top one, the bottom one and the TE classes) and TE families. Every ribbon is drawn here, whereas Figure 7H shows the same comparison with ribbons carrying at least five GO terms, as stated in its legend; that filter hides 36 class-to-group and 50 group-to-family ribbons, together accounting for 146 GO terms.**

**Supplementary Figure 10. Extended connection map of GO terms per TE family, of which Figure 6A is the simplified view. Up to 30 terms per family are shown, with the same edge and term-size filters as Supplementary Figure 6, and node color denotes the FDR-corrected enrichment p-value.**

**Supplementary Figure 11. Concordance of the enrichment analysis across TSS window sizes and of the Gene Ontology analysis across gene-set cut-offs. (A) Observed to random odds ratio for every TE class and family at 5, 10 and 20 kb; hue denotes the TE class, and color tint shows the window size. (B) Bland-Altman comparison of the 44 families between each pair of windows, with the mean difference and the limits of agreement; points are colored by TE class. (C) Number of significant GO terms per group at the 5% and 10% cut-offs, and the fraction of the 5% terms that remain significant at 10%; the left annotation colormap carries the TE class and divergence colors used in Figures 4B, 5B and 6B. (D) Stability of 9 selected results of TE groups functional enrichment under all six combinations of window size and cut-off, with filled circles colored by the FDR-corrected enrichment p-value on the same scale as the connection maps, and open circles marking a result that is not significant or absent.**

**Supplementary Figure 12.** Stability of the gene sets across TSS window sizes. Overlap coefficient of the top 5% gene sets and Kendall correlation of the gene rankings with 95% bootstrap confidence intervals, between each pair of window sizes, for each TE class and for all TEs. The Kendall correlations are raw, as each is a single test.

**Supplementary Figure 13. Sensitivity of the Gene Ontology results across window size and gene-set cut-off. (A) Number of significant GO terms in each of the six conditions (three TSS window sizes by two gene-set percentiles), for each of the three levels of analysis; cell color is the term count relative to the reported condition, which is outlined in black frames. (B) Barplot showing the fraction of the reported condition's terms that remain significant and the Jaccard index of the two term sets for each condition. The preserved fraction was added since Jaccard could be deflated by gained GO terms. (C) Bubble plot showing TE groups keeping at least one significant GO term in each condition, ordered by the number of conditions survived.**

**TE_intersections gives all 38,704 TSS neighborhoods with the elements within 10 kb of each: counts and mean divergence per class, and the comma-joined lists of subfamilies, families, classes and divergence values. Counts are per TSS rather than per gene, so a gene with several annotated TSS contributes several rows. Sheets enrichment_classes, enrichment_families and enrichment_subfamilies give the observed and random odds ratios, the raw and FDR-adjusted Fisher p-values and the empirical permutation p-values for the six classes, the 44 families and all 1,143 subfamilies respectively.**

**Supplementary File 1. Enrichment map of TSS 10Kb neighborhoods by TEs (sheet TSS_TE_intersections) and enrichment statistics at the level of TE classes (sheet enrichment_classes), families (enrichment_families) and subfamilies (enrichment_subfamilies).**

**Supplementary File 2. The gene sets used for Gene Ontology analysis, in long format. Sheet by_TE_group gives the top 5% of genes by element count for each TE group together with the TE-top and TE-bottom sets; sheet by_divergence gives the highest- and lowest-divergence 5% of genes per class; sheet by_family gives the top 5% of genes for each of the 44 families. Each row is one gene with its rank in its set.**

**Supplementary File 3. Gene Ontology enrichment at FDR < 0.05, with the manual functional group classification. Sheet GO_TE_groups covers the TE class, TE-top and TE-bottom gene sets, sheet GO_by_divergence the divergence-stratified sets and sheet GO_by_family the 44 families. Every sheet carries both the raw enrichment p-value and the FDR-adjusted value, together with the fold enrichment, the overlapping genes and the number of human genes annotated to the term.**

**Supplementary File 4. The interferon-alpha domain in-depth data, the T2T assembly comparison to hg38 and the overlap with the prior research by (Lu et al. 2020). Sheets IFNA_elements, IFNA_tests and IFNA_subfamily_composition give all 175 elements of the 220 kb domain, the four domain tests with their null distributions and empirical p-values, and the L1 subfamily composition. Sheet assembly_bound gives the T2T sequence and the share of elements, windows and genes it contributes, by class, family and chromosome, compared to the hg38 reference. Sheets prior_work_overlap_matrix, prior_work_categories and prior_work_shared_genes give the gene-set comparison with the published mouse categories, mapped through orthology.**

**Supplementary File 5. Sensitivity analyses across TSS window size and gene-set cut-off. Sheets window_classes, window_families, window_concordance and window_flips give the enrichment statistics at 5, 10 and 20 kb with their pairwise concordance and changes of significance status. Sheets percentile_summary and percentile_terms provide the Gene Ontology comparison between the 5% and 10% gene-set cut-offs. Sheets GO_grid_index, GO_grid_preservation, GO_grid_terms, GO_grid_concordance and headline_by_condition give the Gene Ontology results for all six combinations of window size and percentile, the fraction of the reported terms preserved in each, GO terms gained or lost, and the nine selected results under every combination. Sheets geneset_stability and rank_stability contain the gene-set overlap and rank agreement between the TSS window sizes.**

**Supplementary File 6.** Extended discussion. The TSS neighborhood size literature review, the per-class and per-family mechanistic review of the current results.
