# Supplementary material for: Telomere-to-telomere co-mapping of transposable elements and human genes identifies a cluster of young L1 elements in the interferon-alpha domain with potential evolutionary arms-race
Source: G3 (Bethesda). 2026 Sep 23;16(11):jkag250. doi: 10.1093/g3journal/jkag250 (PMC13732488; doi:10.1093/g3journal/jkag250)
Supplement: Supplementary file 1 [file jkag250_supplementary_data.zip › Supplementary File 6.docx]

# **Extended discussion for: Telomere-to-telomere co-mapping of transposable elements and human genes identifies a cluster of young L1 elements in the interferon-alpha domain with potential evolutionary arms-race**

Four the most important topics are discussed here; in the order a reader of the main article would want them. The first is the case for the 10 kb proximity TSS window, compared with the windows other groups have used, since this conventional selection governs every number in the. The second is the review of mechanistic causes of each transposable element (TE) class being co-associated relative to transcription start sites: insertion machinery, selection, neutral evolutionary mechanisms, and the timing of germline activation. The third is the functional interpretation of the gene groups that are richest and poorest in TEs. The fourth is the family-level review, which is where the specific literature on Alu, MIR, SVA, LTR and DNA transposon function is reviewed and discussed in the comparison to the current findings, together with the interferon-alpha domain in its wider immunological context.

# 1. **10 kb TSS neighborhood as the optimal albeit conventional window for the TE evolutionary genomics studies**

In the present study we used a co-mapping window of 10 kb, 5 kb upstream and 5 kb downstream, to select the TEs that are inserted in a vicinity of a given human TSS. 5 kb and 20 kb windows were used as comparator neighborhoods to evaluate the robustness of the observed trends. This window or the comparable length windows were used previously in a series of functional epigenomics TE studies. A study of IFN-inducible enhancers being spread in human genome by LTR elements utilized a 10 kb window to assess LTRs enrichment by binomial test (Chuong et al. 2016), whereas we used Fisher exact test for the same purpose. A landmark study (Correa et al. 2021) showed enrichment of LINEs and LTRs near duplicated genes and SINEs and DNA elements near singleton genes, and utilized two proximity windows: 4 kb and 20 kb ones. In addition, one of the first studies of TE functional and epigenomic impact (Bourque et al. 2008) relied on 5 kb upstream and downstream of coding exons as gene-proximal region for transcription factor (TF) - TE binding colocalization. Another landmark genomic proximity-based investigation of LTR elements functional impact (Ito et al. 2017) utilized a shifted 6 kb window: the upstream 5 kb and downstream 1 kb for each TSS were selected. Finally, a series of TF and chromatin modifications studies of retroelements performed by our consortium (Nikitin et al. 2018; Igolkina et al. 2019; Nikitin et al. 2019 Oct 1; Nikitin, Garazha, et al. 2019; Nikitin, Kolosov, et al. 2019; Nikitin 2025 Sep 27) relied on the same 10 kb window as applied here.

The current analysis validates this approach by highlighting the importance of permutation-based random corrections to account for the variable length of different TE classes. Because LINE elements (averaging 6 kb in full length) occupy a much larger genomic footprint than Alu elements (300 bp), they are statistically more likely to intersect a 10 kb window by chance (Levin et al. 2025). Conventional statistical methods, such as the Fisher exact test, treat each element as a point-like entity (Kanduri et al. 2018), which can lead to the artificial underestimation of the regulatory impact of shorter elements or the overestimation of longer ones. The use of 500 random permutations allows for the derivation of an empirical p-value that correctly identifies biological enrichment over length-driven stochasticity.

# 2. **Evolutionary trends of TE classes being non-randomly distributed in the 10 kb TSS neighborhoods of human genes**

As a necessary preliminary analysis, we studied degree of enrichment of TE classes and families in the 10 kb neighborhood of human gene TSS. The results gained allow us to compare public evidence about human TE proliferation strategies, insertion machineries and preferences, as well as evolutionary conflicts, with the trends reported here based on the complete human genome assembly.

The enrichment of SINEs and SVAs near TSS (1.468- and 1.368-fold enrichment over the random expectation) is a consequence of their successful exploitation of the L1 insertion machinery's preference for open chromatin (Cost et al. 2001; Flasch et al. 2019), a preference that is biologically realized during the hypomethylated window of primordial germ cell (PGC) development (Maupetit-Mehouas and Vaury 2020), specifically for evolutionarily young and active TE families: L1HS (human-specific LINE-1), SVA (subfamilies E and F), and HERV-K (LTR) according to a recent preprint study (Dietmann et al. 2020 Mar 20). Conversely, the depletion of LINEs (0.877-fold change versus the random control) is a signature of purifying selection acting against the deleterious effects of large insertions in gene-regulatory domains, such as ectopic recombination and transcriptional interference (Graham and Boissinot 2006), a force that is partially mitigated but not abolished by the historically low effective population size of humans. On the other hand, an Alu or SVA insertion in a promoter region is less likely to structurally disrupt the nucleosome landscape or terminate transcription than a 6 kb L1 (Daniel et al. 2015). While they may introduce novel transcription factor binding sites (TFBS), the immediate fitness cost is often low (Cordaux et al. 2006).

Our results of SVA elements being enriched near TSS are connected also to the fact that they resist complete repression during the post-fertilization reprogramming window (Dietmann et al. 2020 Mar 20) and are essential for zygote genome activation by acting as regulatory hubs (SVA D subfamily) (DiRusso and Clark 2023).

Moreover, SVA elements are the youngest TE class in humans with active evolutionary arms race against the host genome (Nikitin 2026 Mar 23). They contain binding sites for key pluripotency factors such as OCT4 and YY1 (Barnada et al. 2022), which creates a potential positive feedback loop: an SVA inserts near a pluripotency gene in a primordial germ cell and binds OCT4, which in turn boosts SVA transcription, leading to more transposition. The SVA may act as an enhancer for the nearby gene via the constructive neutral evolution (CNE) mechanism (Sergio A Muñoz-Gómez et al. 2021) and SVA elements density near the germline-expressed genes gradually amplifies. Finally, SVA elements contain GC-rich regulatory sequences (variable number tandem repeats, VNTRs) that facilitate their retention in gene-rich regions via CNE or exaptation (Gianfrancesco et al. 2019).

DNA transposons (e.g., *TcMar*, *hAT*) utilize a "cut-and-paste" transposase mechanism distinct from the L1 machinery. They have been extinct in the human lineage for millions of years (Li et al. 2025). Their depletion near TSSs (0.938 of the random expectation) likely reflects both a lack of targeting to these specific open chromatin regions (compared to retrotransposons) and the long-term action of purifying selection over deep evolutionary time.

LTR elements, derived from ancient endogenous retroviruses, are also heavily depleted near TSS (0.667-fold change against the random background). Most LTRs in the human genome are "solo" LTRs that remain after host-mediated recombination between the flanking LTRs of a provirus (Kelsey et al. 2025). Because LTRs are potent promoters, their proximity to host genes is a major risk for "onco-exaptation", where the illicit activation of a viral promoter drives the expression of an adjacent oncogene (Wang et al. 2023). Consequently, LTRs are typically sequestered in heterochromatic regions or subject to strict epigenetic silencing (Stamidis and Żylicz 2023). For the full-length 6-8 kb endogenous retroviruses, their insertion in the TSS proximity could be deleterious by disrupting promoter-enhancer interactions and perturbing the host authentic TFBS (Maksakova et al. 2006).

At the level of TE families, our results reveal a more complex regulatory landscape. Only 7 out of 44 families are significantly enriched near TSS: hAT-Charlie, MIR, CR1, Alu, SVA, TcMar-Tc1 and L2. The enrichment of the L2 family (1.230-fold change) is particularly notable because L2 elements have undergone extensive mutational decay in the last 100 million years, becoming a dominant LINEs in monotremes but surpassing their dominance to L1 in eutherians (Suh et al. 2014). Eventually they lost their autonomous propagation ability and became "domesticated" as TSS for microRNAs in the host genes 3′ UTRs, particularly in the brain where they are a major source of microRNAs (Petri et al. 2019; Zottel et al. 2020). They are also coopted as enhancers in tissue-shared compared to tissue-specific genes, highly enriched in H3K27ac and H3K4me1 marks, emphasizing their transition from genetic parasites to functional regulatory modules via exaptation or CNE (Roller et al. 2021). Our findings suggest that such functional interdependency could save L2 elements from deletion in the gene-proximal regulatory active genomic regions.

The CR1 family (Chicken repeats, LINE) also shows significant enrichment (1.051-fold change), with surprising functional connections to neurotransmitter processes and postsynaptic receptor internalization. This suggests that specific waves of LINE insertions, long before the dominance of L1, contributed to the foundational regulatory networks of the amniotes brain and these insertions could be remnants of those events (Suh et al. 2014).

Whereas SINEs showed 1.468-fold enrichment near TSS, their major families, Alu and MIR, exhibited 1.513- and 1.377-fold enrichment relative to random OR. Alus are considered "proto enhancers" that evolve into functional regulatory elements over time (Su et al. 2014). They are specifically enriched in active chromatin marks such as H3K36me3 in the colon and brain (Hyacinthe and Bourque 2024 Nov 19), and they frequently accumulate TFBS that rewire host networks (Häsler and Strub 2006). MIR elements were shown here as strongly associated with nervous system genes and voltage-gated potassium channels. MIRs are uniquely involved in the naïve pluripotent state, where they are co-opted by ESRRB to build networks of enhancers and super-enhancers regulating pluripotency (Cipta et al. 2025).

Finally, we observed no connection of family enrichment near TSS with their divergence for any TE class including LINEs, albeit the comparison of newly integrated, polymorphic and human specific L1 insertions (Chen et al. 2020) suggested that L1 are integrating in gene rich but are persisting in a genome for long term in gene poor regions. Nevertheless, our current analysis is concentrated on the evolutionary ancient LINEs with peak divergence of 270 corresponding to more than 100 million years of evolutionary age (Kosuge et al. 2024).

# 3. **The gene groups richest and poorest in transposable elements**

On average, according to our results, each human gene harbors 15.05 TEs in its TSS neighborhood, with SINEs being the most frequent (7.82 per gene), followed by LINEs (4.39), DNA elements (1.49) and LTRs (1.32). The identification of *CIB3* as a genomic outlier, containing 42 TEs of various classes, points toward the existence of extreme repeat accumulation zones. *CIB3* (calcium and integrin binding protein 3) is integral to the mechano-electrical transduction (MET) apparatus in the inner ear, forming heteromeric complexes with TMC1 and TMC2 to stabilize cation channels (Liang et al. 2021). The high repeat density surrounding such a specialized sensory gene raises questions about whether these TEs provide modular regulatory controls for high-precision environmental sensing or if the locus simply resides in a genomic region with relaxed purifying selection.

Genes involved in embryogenesis, transcription, and nervous system development are consistently found in the "TE bottom" group - those with the lowest number of TE insertions. This result strongly supports the "robustness hypothesis", which posits that core biological pathways must be protected from the regulatory noise introduced by TEs and TEs are purged by negative selection in these pathways. Embryogenesis is a process that requires extreme transcriptional precision; even minor disruptions to the timing or level of gene expression can be lethal (DiRusso and Clark 2023), albeit TEs largely contribute to zygotic genome activation (Oomen et al. 2025). The rest two large groups, sensory system and signaling pathways, were present mainly in the TE bottom group and neither of TE classes nor families provided more enriched GO terms than the TE bottom group did (Figure 4B).

Conversely, genes enriched with TEs (the "TE top" group) are involved in RNA splicing, DNA repair, telomere maintenance, and immune signaling. These processes represent the "innovation laboratory" where TEs are actively co-opted to increase transcriptome and proteome complexity (Garcia-Perez et al. 2016).

- **RNA splicing:** Alu elements were early shown to profoundly impact RNA splicing by introducing new splice sites (exonization), acting as alternative exon sinks, and forming double-stranded structures that alter exon inclusion (Payer et al. 2018). We now show that Alu copies are significantly enriched near the host genes related to splicing, which is a completely orthogonal mean of Alu-mediated impact on splicing.
- **DNA repair:** The enrichment of TEs (particularly Alu elements) near DNA repair genes (double strand breaks and mismatch repair) could suggest a synergistic relationship where the more robust responses are evolving to DNA lesions induced by insertions of these elements (Morales et al. 2015).
- **Olfactory receptors:** L1 elements are specifically enriched near olfactory receptor genes, which could allow rapid diversification of sensory perception of smell sensing through TE-mediated rearrangements, leading to genes duplication and subfunctionalization or gene losses (Beck et al. 2011; Redaelli et al. 2024).

Other groups: metals metabolism, other (specific) metabolism, cytoskeleton, cell death, DNA recombination and DNA replication did not appear in the TE bottom group and, despite the highly variable total GO terms count or non-significant Fisher exact test results, can be considered as TE-enriched*.*

# 4. **Family level enrichment of transposable elements and the interferon-alpha domain enrichment**

At the level of TE classes, a previous landmark study (Lu et al. 2020) utilized 20 kb and 2 kb window to show non-random association of SINEs with housekeeping genes having “broad” promoters with multiple TSS and LINEs with tissue-specific ones having “sharp” promoters with single TSS (Haberle and Stark 2018). Precisely, in that study SINE-enriched genes were associated with ribosome, translation, RNA processing, nucleolus and protein transport, whereas LINE elements were enriched in genes of olfactory receptors, retinol metabolism, epoxygenase P450 pathway and immunoglobulin domains. Our analysis partly confirms these findings. Similarly, we show that RNA processing, ribosome and translation, transcription and general pathways are enriched in SINEs. But such highly cell-type specific processes as embryogenesis and sensory system were also present as a minor part of SINE elements. For LINEs we confirmed olfactory receptors, whereas lipid metabolism contained different terms: flavone metabolism and negative regulation of fatty acids metabolism. Also, for L1 elements we report TF activity and calcium ion binding. These partial differences can be explained by the methodological differences: Lu et al (Lu et al. 2020) profiled TE abundance separately in Promoter, Intron, Downstream, 5′ UTR, CDS and 3′ UTR regions of each gene, then performed quantile normalization and hierarchical clustering, analyzing gene groups arising from this clustering, and did the analysis on mice genes. As we derived, only 270 of our current SINE-enriched genes overlap with the 2,041 genes reported as SINE enriched by (Lu et al. 2020). Such a weak intersection, albeit significantly above the random background, can explain the differences in results between the two analyses.

At the level of families, Alu elements are of particular interest. In our analysis they were found near 90.4% of all human TSS, with up to 29 copies in a single 10 kb window. One of the first functional TE mapping study showed that Alus are preferentially inserted near metabolism, transport and signaling genes, whereas structural and information processing genes were depleted of Alu elements (Grover et al. 2003). Here we show the contrasting results that Alu are enriched near RNA processing genes, potentially affecting also genes of meiosis, DNA damage response, inner mitochondrial membrane and transcription initiation and elongation. These drastic differences can be explained by the fact that (Grover et al. 2003) used chromosome 21 and 22 in their analyses, whereas here the complete human genome assembly is used.

Alu elements enrichment near genes of RNA processing is corroborated by the recent evidence: Alu elements often provide cryptic splice sites that lead to exonization and exon skipping in primates in a lineage specific manner (Denisko et al. 2025 Mar 11). This process is regulated by hnRNP C (Daniel et al. 2015) and the Ku70/80 heterodimer (the latter one according to the recent preprint articles (Pascarella et al. 2025 Nov 20; Yu et al. 2025 Dec 18)), which compete with splicing factors to prevent the deleterious over-inclusion of Alu sequences while allowing for adaptive variations in the proteome. Furthermore, the presence of Alus near DNA repair genes suggests their role as "editing inducer elements", where inverted Alu repeats could facilitate the A-to-I editing of host transcripts, thereby fine-tuning enzymatic function in a primate-specific manner (Daniel et al. 2015).

The MIR (Mammalian-wide Interspersed Repeat) family, though less numerous than Alus, showed strong functional preference for the nervous system in our analysis. MIR elements are significantly enriched near genes for voltage-gated potassium channels, which are the largest and most diverse ion channel family in the human genome (Humphries and Dart 2015). Potassium channels are essential for returning the cell to a resting state after an action potential (Urrutia et al. 2024). The association of MIR elements with these channels suggests that MIR-derived enhancers may contribute to the complex rules of subcellular localization and firing frequency that differentiate mammalian neurons (Ranjan et al. 2019). Furthermore, MIR elements are linked to macrophage activation and the sensing of metal ions, indicating they could also provide regulatory modules for ancient immune and metabolic responses.

SVA elements were previously shown to impact host genes via premature termination based on their internal polyadenylation signal (Hancks and Kazazian 2010) and they are now shown to enrich near genes related to termination of RNA polymerase II transcription, precisely the core catalytic RNA polymerase II subunit and its C-terminal domain phosphatases (3 SVA B copies in the 116 kb region of chromosome 11). A previous genomic proximity-based study revealed SVA enrichment near zinc finger clusters (Gianfrancesco et al. 2019), albeit the methodology was different: (Gianfrancesco et al. 2019) used hg19 human genome and 1Mb bins for enrichment testing instead of 10 kb and T2T genome in the current analysis. SVA elements are also known to facilitate 3' transduction events when their internal polyadenylation signal is bypassed by Pol II (Kirby et al. 2025). The proximity of SVA elements to the genes that regulate Pol II termination suggests a potential co-evolutionary loop where SVAs have been integrated into the feedback mechanisms that control transcriptional processivity. This could represent a mechanism of CNE, where the host genome becomes functionally dependent on the presence of a TE-derived regulatory element for the accurate execution of core transcriptional cycles (Sergio A. Muñoz-Gómez et al. 2021). Despite recent studies have identified human-specific subfamilies, such as SVA_F1, which are active in the human population and frequently mobilize adjacent gene sequences through transduction (Kirby et al. 2025) – the RNA polymerase termination genes are enriched with SVA_B subfamily, so their SVA regulatory impact is likely not a recent evolutionary innovation.

Among the TE classes, only LTR families all demonstrated functional associations with human molecular processes. Interestingly, Gypsy elements which belong to the chromoviruses group targeting themselves into heterochromatin regions via chromodomain (Gao et al. 2008), are strongly co-associated with translation activator genes according to the current analysis.

DNA transposons, which have been extinct in the human lineage for millions of years, are mostly functionally neutral. However, the hAT-Charlie family shows a significant association with the MHC class I pathway, suggesting that ancient DNA transposon insertions contributed to the organization of the vertebrate adaptive immune system before their eventual inactivation.

It is important to note than the fact that certain TE families, precisely 30 out of 44 ones (Helitrons, 20 DNA families, 4 SINE ones, 5 LINE families and no LTR ones) did not result in significant functional host gene groups by GO analysis, suggests that their presence could be largely neutral - a result of random drift and tolerance rather than adaptive recruitment.

At the level of lowest and highest divergence by TE classes, ancient high-divergence SINEs (particularly the MIR family) and high-divergence LINEs (CR1, L1, Dong-R4 family) were strongly associated with the nervous system according to our results. Genes with high average TE divergence are enriched for processes such as postsynaptic membrane organization, synaptic chemical transmission, and voltage-gated potassium channels. This pattern suggests that the foundational architecture of the mammalian brain was shaped by ancestral waves of TE activity that have since been stabilized and domesticated into the host's regulatory framework (Ferrari et al. 2021). High-divergence SINEs are particularly prominent near ion channel genes, which regulate the electrical excitability of neurons (Urrutia et al. 2024). The stabilization of these elements over hundreds of millions of years suggests they have evolved from genetic parasites into essential cis-regulatory modules that buffer the transcriptional output of housekeeping genes or provide tissue-specific enhancer logic.

One of the most biologically significant findings that we can report is the identification of a 220 kb patch on chromosome 9 containing a cluster of interferon alpha genes (*IFNA10, IFNA16, IFNA17, IFNA21, IFNA4, IFNA6, IFNA7, IFNW1*) that is uniquely enriched with young, low-divergence L1 elements. There are 12 RefSeq curated genes in this area according to the T2T genome assembly, which is significantly higher than an average human genome value of 12.5 genes per 1 Mb (Homo sapiens genome assembly T2T-CHM13v2.0 - NCBI - NLM).

The enrichment of young LINEs in this region could suggest an ongoing evolutionary arms race. Type I interferons are the first responders to viral infection (Moreau et al. 2023), and their signaling is known to be heavily influenced by TE-derived promoters, enhancers and TE exonization (Apostolou and Thanos 2008; Pasquesi et al. 2024). Additionally, interferon I signaling is regulated by L1 elements repression via HUSH complex which detects L1-derived dsRNAs (Tunbak et al. 2020). The presence of low-divergence elements indicates that these insertions are relatively recent and may be contributing to the diversification of the human antiviral response. Moreover, Alu and L1 elements can accumulate in the pre-existing loci already occupied by copies of themselves, creating a feed-forward loops of the progressive expansion in limited genome regions (Hu et al. 2025 Mar 2). This finding has profound implications for understanding autoimmune diseases like systemic lupus erythematosus (SLE), which is characterized by a type I interferon signature (Eloranta and Rönnblom 2016). The de-repression of TEs in these regions, perhaps due to environmental stressors or aging-related heterochromatin loss as was shown earlier (Kelly et al. 2018), can lead to the formation of double-stranded RNA (dsRNA) that triggers the cGAS-STING pathway (Gázquez-Gutiérrez et al. 2021) and eventually a constitutive immune response that leads to chronic inflammation (Kelly et al. 2018).

# 5. **Literature cited**

Apostolou E, Thanos D. 2008. Virus Infection Induces NF-κB-Dependent Interchromosomal Associations Mediating Monoallelic IFN-β Gene Expression. Cell. 134(1):85–96. doi:10.1016/j.cell.2008.05.052. [accessed 2026 Feb 3]. https://www.cell.com/action/showFullText?pii=S0092867408007599.

Barnada SM, Isopi A, Tejada-Martinez D, Goubert C, Patoori S, Pagliaroli L, Tracewell M, Trizzino M. 2022. Genomic features underlie the co-option of SVA transposons as cis-regulatory elements in human pluripotent stem cells. PLoS Genet. 18(6):e1010225. doi:10.1371/JOURNAL.PGEN.1010225. [accessed 2026 Feb 1]. https://pmc.ncbi.nlm.nih.gov/articles/PMC9239442/.

Beck CR, Garcia-Perez JL, Badge RM, Moran J V. 2011. LINE-1 Elements in Structural Variation and Disease. Annu Rev Genomics Hum Genet. 12:187. doi:10.1146/ANNUREV-GENOM-082509-141802. [accessed 2026 Feb 3]. https://pmc.ncbi.nlm.nih.gov/articles/PMC4124830/.

Bourque G, Leong B, Vega VB, Chen X, Yen LL, Srinivasan KG, Chew JL, Ruan Y, Wei CL, Huck HN, et al. 2008. Evolution of the mammalian transcription factor binding repertoire via transposable elements. Genome Res. 18(11):1752. doi:10.1101/GR.080663.108. [accessed 2026 Jan 30]. https://pmc.ncbi.nlm.nih.gov/articles/PMC2577865/.

Chen D, Cremona MA, Qi Z, Mitra RD, Chiaromonte F, Makova KD. 2020. Human L1 Transposition Dynamics Unraveled with Functional Data Analysis. Mol Biol Evol. 37(12):3576. doi:10.1093/MOLBEV/MSAA194. [accessed 2026 Jan 31]. https://pmc.ncbi.nlm.nih.gov/articles/PMC7743743/.

Chuong EB, Elde NC, Feschotte C. 2016. Regulatory evolution of innate immunity through co-option of endogenous retroviruses. Science. 351(6277):1083. doi:10.1126/SCIENCE.AAD5497. [accessed 2026 Jan 30]. https://pmc.ncbi.nlm.nih.gov/articles/PMC4887275/.

Cipta NO, Zeng Y, Wong KW, Zheng ZH, Yi Y, Warrier T, Teo JZ, Teo JHJ, Kok YJ, Bi X, et al. 2025. Rewiring of SINE-MIR enhancer topology and Esrrb modulation in expanded and naive pluripotency. Genome Biology 2025 26:1. 26(1):107-. doi:10.1186/S13059-025-03577-8. [accessed 2026 Feb 1]. https://link.springer.com/article/10.1186/s13059-025-03577-8.

Cordaux R, Lee J, Dinoso L, Batzer MA. 2006. Recently integrated Alu retrotransposons are essentially neutral residents of the human genome. Gene. 373(1–2):138–144. doi:10.1016/j.gene.2006.01.020. [accessed 2026 Jan 31]. https://pubmed.ncbi.nlm.nih.gov/16527433/.

Correa M, Lerat E, Birmelé E, Samson F, Bouillon B, Normand K, Rizzon C. 2021. The Transposable Element Environment of Human Genes Differs According to Their Duplication Status and Essentiality. Genome Biol Evol. 13(5). doi:10.1093/GBE/EVAB062. [accessed 2026 Jan 30]. https://pubmed.ncbi.nlm.nih.gov/33973013/.

Cost GJ, Golding A, Schlissel MS, Boeke JD. 2001. Target DNA chromatinization modulates nicking by L1 endonuclease. Nucleic Acids Res. 29(2):573. doi:10.1093/NAR/29.2.573. [accessed 2026 Jan 31]. https://pmc.ncbi.nlm.nih.gov/articles/PMC29664/.

Daniel C, Behm M, Öhman M. 2015. The role of Alu elements in the cis-regulation of RNA processing. Cell Mol Life Sci. 72(21):4063. doi:10.1007/S00018-015-1990-3. [accessed 2026 Jan 31]. https://pmc.ncbi.nlm.nih.gov/articles/PMC11113721/.

Denisko D, Kim J, Ku J, Zhao B, Lee EA. 2025 Mar 11. Inverted Alu repeats in loop-out exon skipping across hominoid evolution. bioRxiv. doi:10.1101/2025.03.07.642063. [accessed 2026 Apr 1]. https://pubmed.ncbi.nlm.nih.gov/40161837/.

Dietmann S, Keogh MJ, Tang W, Magnusdottir E, Kobayashi T, Chinnery PF, Surani MA. 2020 Mar 20. Transposable elements resistant to epigenetic resetting in the human germline are epigenetic hotspots for development and disease. bioRxiv.:2020.03.19.998930. doi:10.1101/2020.03.19.998930. [accessed 2026 Feb 1]. https://www.biorxiv.org/content/10.1101/2020.03.19.998930v1.

DiRusso JA, Clark AT. 2023. Transposable elements in early human embryo development and embryo models. Curr Opin Genet Dev. 81:102086. doi:10.1016/J.GDE.2023.102086. [accessed 2026 Feb 2]. https://www.sciencedirect.com/science/article/pii/S0959437X23000667?via%3Dihub.

Eloranta ML, Rönnblom L. 2016. Cause and consequences of the activated type I interferon system in SLE. J Mol Med (Berl). 94(10):1103–1110. doi:10.1007/S00109-016-1421-4. [accessed 2026 Feb 3]. https://pubmed.ncbi.nlm.nih.gov/27094810/.

Ferrari R, Grandi N, Tramontano E, Dieci G. 2021. Retrotransposons as Drivers of Mammalian Brain Evolution. Life 2021, Vol 11, Page 376. 11(5):376. doi:10.3390/LIFE11050376. [accessed 2026 Apr 2]. https://www.mdpi.com/2075-1729/11/5/376/htm.

Flasch DA, Macia Á, Sánchez L, Ljungman M, Heras SR, García-Pérez JL, Wilson TE, Moran J V. 2019. Genome-wide de novo L1 Retrotransposition Connects Endonuclease Activity with Replication. Cell. 177(4):837-851.e28. doi:10.1016/j.cell.2019.02.050. [accessed 2026 Jan 31]. https://www.cell.com/action/showFullText?pii=S0092867419302338.

Gao X, Hou Y, Ebina H, Levin HL, Voytas DF. 2008. Chromodomains direct integration of retrotransposons to heterochromatin. Genome Res. 18(3):359–369. doi:10.1101/GR.7146408. [accessed 2026 Feb 2]. http://genome.cshlp.org/content/18/3/359.full.

Garcia-Perez JL, Widmann TJ, Adams IR. 2016. The impact of transposable elements on mammalian development. Development. 143(22):4101. doi:10.1242/DEV.132639. [accessed 2026 Feb 2]. https://pmc.ncbi.nlm.nih.gov/articles/PMC5830075/.

Gázquez-Gutiérrez A, Witteveldt J, Heras SR, Macias S. 2021. Sensing of transposable elements by the antiviral innate immune system. RNA. 27(7):735. doi:10.1261/rna.078721.121. [accessed 2026 Apr 1]. https://pmc.ncbi.nlm.nih.gov/articles/PMC8208052/.

Gianfrancesco O, Geary B, Savage AL, Billingsley KJ, Bubb VJ, Quinn JP. 2019. The Role of SINE-VNTR-Alu (SVA) Retrotransposons in Shaping the Human Genome. International Journal of Molecular Sciences 2019, Vol 20,. 20(23). doi:10.3390/IJMS20235977. [accessed 2026 Feb 1]. https://www.mdpi.com/1422-0067/20/23/5977.

Graham T, Boissinot S. 2006. The Genomic Distribution of L1 Elements: The Role of Insertion Bias and Natural Selection. J Biomed Biotechnol. 2006:75327. doi:10.1155/JBB/2006/75327. [accessed 2026 Jan 31]. https://pmc.ncbi.nlm.nih.gov/articles/PMC1510949/.

Grover D, Majumder PP, Rao CB, Brahmachari SK, Mukerji M. 2003. Nonrandom distribution of alu elements in genes of various functional categories: insight from analysis of human chromosomes 21 and 22. Mol Biol Evol. 20(9):1420–1424. doi:10.1093/MOLBEV/MSG153. [accessed 2026 Feb 1]. https://pubmed.ncbi.nlm.nih.gov/12832639/.

Haberle V, Stark A. 2018. Eukaryotic core promoters and the functional basis of transcription initiation. Nat Rev Mol Cell Biol. 19(10):621. doi:10.1038/s41580-018-0028-8. [accessed 2026 Apr 1]. https://pmc.ncbi.nlm.nih.gov/articles/PMC6205604/.

Hancks DC, Kazazian HH. 2010. SVA retrotransposons: Evolution and genetic instability. Semin Cancer Biol. 20(4):234. doi:10.1016/J.SEMCANCER.2010.04.001. [accessed 2026 Jan 31]. https://pmc.ncbi.nlm.nih.gov/articles/PMC2945828/.

Häsler J, Strub K. 2006. Alu elements as regulators of gene expression. Nucleic Acids Res. 34(19):5491. doi:10.1093/NAR/GKL706. [accessed 2026 Feb 1]. https://pmc.ncbi.nlm.nih.gov/articles/PMC1636486/.

Homo sapiens genome assembly T2T-CHM13v2.0 - NCBI - NLM. [accessed 2026 Feb 3]. https://www.ncbi.nlm.nih.gov/datasets/genome/GCF_009914755.1/.

Hu Z, Xu B, Zhang Xiaoyan, Zhang Xiaou-ou, Weng Z, Yu T. 2025 Mar 2. LOCATE: using Long-read to Characterize All Transposable Elements. bioRxiv.:2025.02.26.640385. doi:10.1101/2025.02.26.640385. [accessed 2026 Jan 31]. https://www.biorxiv.org/content/10.1101/2025.02.26.640385v2.

Humphries ESA, Dart C. 2015. Neuronal and Cardiovascular Potassium Channels as Therapeutic Drug Targets: Promise and Pitfalls. J Biomol Screen. 20(9):1055. doi:10.1177/1087057115601677. [accessed 2026 Apr 3]. https://pmc.ncbi.nlm.nih.gov/articles/PMC4576507/.

Hyacinthe J, Bourque G. 2024 Nov 19. Transposable elements impact the human regulatory landscape through cell type specific epigenomic associations. bioRxiv.:2024.08.07.606967. doi:10.1101/2024.08.07.606967. [accessed 2026 Feb 1]. https://www.biorxiv.org/content/10.1101/2024.08.07.606967v2.

Igolkina AA, Zinkevich A, Karandasheva KO, Popov AA, Selifanova M V., Nikolaeva D, Tkachev V, Penzar D, Nikitin DM, Buzdin A. 2019. H3K4me3, H3K9ac, H3K27ac, H3K27me3 and H3K9me3 Histone Tags Suggest Distinct Regulatory Evolution of Open and Condensed Chromatin Landmarks. Cells. 8(9). doi:10.3390/CELLS8091034. [accessed 2026 Jan 30]. https://pubmed.ncbi.nlm.nih.gov/31491936/.

Ito J, Sugimoto R, Nakaoka H, Yamada S, Kimura T, Hayano T, Inoue I. 2017. Systematic identification and characterization of regulatory elements derived from human endogenous retroviruses. PLoS Genet. 13(7):e1006883. doi:10.1371/JOURNAL.PGEN.1006883. [accessed 2026 Jan 30]. https://pmc.ncbi.nlm.nih.gov/articles/PMC5529029/.

Kanduri C, Bock C, Gundersen S, Hovig E, Sandve GK. 2018. Colocalization analyses of genomic elements: approaches, recommendations and challenges. Bioinformatics. 35(9):1615. doi:10.1093/bioinformatics/bty835. [accessed 2026 Mar 27]. https://pmc.ncbi.nlm.nih.gov/articles/PMC6499241/.

Kelly M, Lihua S, Zhe Z, Li S, Yoselin P, Michelle P, Sullivan Kathleen E. 2018. Transposable Element Dysregulation in Systemic Lupus Erythematosus and Regulation by Histone Conformation and Hsp90. Clin Immunol. 197:6. doi:10.1016/j.clim.2018.08.011. [accessed 2026 Apr 1]. https://pmc.ncbi.nlm.nih.gov/articles/PMC6258342/.

Kelsey MMG, Kalekar RL, Sedivy JM. 2025. TE-Seq: a transposable element annotation and RNA-Seq pipeline. Mobile DNA 2025 16:1. 16(1):44-. doi:10.1186/S13100-025-00381-W. [accessed 2026 Feb 1]. https://link.springer.com/article/10.1186/s13100-025-00381-w.

Kirby AE, Loftus M, Golba EC, Lee C, Eichler EE, Marschall T, Korbel JO, Zody MC, Zhu Q, Zhou W, et al. 2025. Structural and transduction patterns of human-specific polymorphic SVA insertions. Mob DNA. 16(1):42. doi:10.1186/S13100-025-00373-W. [accessed 2026 Feb 1]. https://pmc.ncbi.nlm.nih.gov/articles/PMC12593883/.

Kosuge M, Ito J, Hamada M. 2024. Landscape of evolutionary arms races between transposable elements and KRAB-ZFP family. Sci Rep. 14(1). doi:10.1038/S41598-024-73752-7. [accessed 2026 Jan 31]. https://pubmed.ncbi.nlm.nih.gov/39375372/.

Levin HL, Lee SP, Anand A. 2025. The 5′ truncation of retrotransposon L1: a process of genome integrity. Genetics. 231(4). doi:10.1093/GENETICS/IYAF202. [accessed 2026 Feb 1]. https://dx.doi.org/10.1093/genetics/iyaf202.

Li TD, Toohill K, Modzelewski AJ. 2025. From Junk DNA to Genomic Treasure: Impacts of Transposable Element DNA, RNA, and Protein in Mammalian Development and Disease. Wiley Interdiscip Rev RNA. 16(4):e70022. doi:10.1002/WRNA.70022. [accessed 2026 Feb 1]. https://pmc.ncbi.nlm.nih.gov/articles/PMC12350819/.

Liang X, Qiu X, Dionne G, Cunningham CL, Pucak ML, Peng G, Kim YH, Lauer A, Shapiro L, Müller U. 2021. CIB2 and CIB3 are auxiliary subunits of the mechanotransduction channel of hair cells. Neuron. 109(13):2131-2149.e15. doi:10.1016/J.NEURON.2021.05.007. [accessed 2026 Feb 1]. https://pure.johnshopkins.edu/en/publications/cib2-and-cib3-are-auxiliary-subunits-of-the-mechanotransduction-c/.

Lu JY, Shao W, Chang L, Yin Y, Li T, Zhang H, Hong Y, Percharde M, Guo L, Wu Z, et al. 2020. Genomic Repeats Categorize Genes with Distinct Functions for Orchestrated Regulation. Cell Rep. 30(10):3296. doi:10.1016/J.CELREP.2020.02.048. [accessed 2026 Jan 30]. https://pmc.ncbi.nlm.nih.gov/articles/PMC7195444/.

Maksakova IA, Romanish MT, Gagnier L, Dunn CA, Van De Lagemaat LN, Mager DL. 2006. Retroviral Elements and Their Hosts: Insertional Mutagenesis in the Mouse Germ Line. PLoS Genet. 2(1):e2. doi:10.1371/journal.pgen.0020002. [accessed 2026 Mar 31]. https://pmc.ncbi.nlm.nih.gov/articles/PMC1331978/.

Maupetit-Mehouas S, Vaury C. 2020. Transposon Reactivation in the Germline May Be Useful for Both Transposons and Their Host Genomes. Cells. 9(5):1172. doi:10.3390/CELLS9051172. [accessed 2026 Feb 1]. https://pmc.ncbi.nlm.nih.gov/articles/PMC7290860/.

Morales ME, White TB, Streva VA, DeFreece CB, Hedges DJ, Deininger PL. 2015. The Contribution of Alu Elements to Mutagenic DNA Double-Strand Break Repair. PLoS Genet. 11(3):e1005016. doi:10.1371/JOURNAL.PGEN.1005016. [accessed 2026 Feb 3]. https://pmc.ncbi.nlm.nih.gov/articles/PMC4356517/.

Moreau TRJ, Bondet V, Rodero MP, Duffy D. 2023. Heterogeneity and functions of the 13 IFN-α subtypes – lucky for some? Eur J Immunol. 53(8):2250307. doi:10.1002/EJI.202250307;JOURNAL:JOURNAL:15214141. [accessed 2026 Feb 3]. /doi/pdf/10.1002/eji.202250307.

Muñoz-Gómez Sergio A, Bilolikar G, Wideman JG, Geiler-Samerotte K. 2021. Constructive Neutral Evolution 20 Years Later. J Mol Evol. 89(3):172–182. doi:10.1007/s00239-021-09996-y. files/1240/Muñoz-Gómez et al. - 2021 - Constructive Neutral Evolution 20 Years Later.pdf.

Muñoz-Gómez Sergio A., Bilolikar G, Wideman JG, Geiler-Samerotte K. 2021. Constructive Neutral Evolution 20 Years Later. J Mol Evol. 89(3):172. doi:10.1007/S00239-021-09996-Y. [accessed 2026 Jan 31]. https://pmc.ncbi.nlm.nih.gov/articles/PMC7982386/.

Nikitin D. 2025 Sep 27. Joint analysis of human retroelements-linked histone modification profiles reveals quickly evolving molecular processes connected with cancer. doi:10.1101/2025.09.24.677146. [accessed 2026 Jan 30]. https://europepmc.org/article/ppr/ppr1091873.

Nikitin D. 2026 Mar 23. Transposable element–host genome evolutionary arms race revealed by multi-modal epigenomic profiling in a telomere-to-telomere human genome reference. bioRxiv.:2026.03.19.712972. doi:10.64898/2026.03.19.712972. [accessed 2026 Apr 4]. https://www.biorxiv.org/content/10.64898/2026.03.19.712972v1.

Nikitin D, Garazha A, Sorokin M, Penzar D, Tkachev V, Markov A, Gaifullin N, Borger P, Poltorak A, Buzdin A. 2019. Retroelement-Linked Transcription Factor Binding Patterns Point to Quickly Developing Molecular Pathways in Human Evolution. Cells. 8(2). doi:10.3390/CELLS8020130. [accessed 2026 Jan 30]. https://pubmed.ncbi.nlm.nih.gov/30736359/.

Nikitin D, Kolosov N, Murzina A, Pats K, Zamyatin A, Tkachev V, Sorokin M, Kopylov P, Buzdin A. 2019. Retroelement-Linked H3K4me1 Histone Tags Uncover Regulatory Evolution Trends of Gene Enhancers and Feature Quickly Evolving Molecular Processes in Human Physiology. Cells 2019, Vol 8,. 8(10). doi:10.3390/CELLS8101219. [accessed 2026 Jan 30]. https://www.mdpi.com/2073-4409/8/10/1219.

Nikitin D, Penzar D, Garazha A, Sorokin M, Tkachev V, Borisov N, Poltorak A, Prassolov V, Buzdin AA. 2018. Profiling of Human Molecular Pathways Affected by Retrotransposons at the Level of Regulation by Transcription Factor Proteins. Front Immunol. 9(JAN). doi:10.3389/FIMMU.2018.00030. [accessed 2026 Jan 30]. https://pubmed.ncbi.nlm.nih.gov/29441061/.

Nikitin D, Sorokin M, Tkachev V, Garazha A, Markov A, Buzdin A. 2019 Oct 1. RetroSpect, a New Method of Measuring Gene Regulatory Evolution Rates Using Co-mapping of Genomic Functional Features with Transposable Elements. Evolution, Origin of Life, Concepts and Methods.:85–111. doi:10.1007/978-3-030-30363-1_5. [accessed 2026 Jan 30]. https://link.springer.com/chapter/10.1007/978-3-030-30363-1_5.

Oomen ME, Rodriguez-Terrones D, Kurome M, Zakhartchenko V, Mottes L, Simmet K, Noll C, Nakatani T, Mourra-Diaz CM, Aksoy I, et al. 2025. An atlas of transcription initiation reveals regulatory principles of gene and transposable element expression in early mammalian development. Cell. 188(4):1156-1174.e20. doi:10.1016/j.cell.2024.12.013. [accessed 2026 Apr 1]. https://www.cell.com/action/showFullText?pii=S0092867424014260.

Pascarella G, Mikhova M, Parkhi G, Godfrey J, Heyza J, Janovič T, Olive A, Carninci P, Schmidt JC, Meek K. 2025 Nov 20. Ku limits aberrant mRNA splicing promoted by intronic antisense Alu elements. bioRxiv. doi:10.1101/2025.11.20.689478. [accessed 2026 Apr 1]. https://pubmed.ncbi.nlm.nih.gov/41332606/.

Pasquesi GIM, Allen H, Ivancevic A, Barbachano-Guerrero A, Joyner O, Guo K, Simpson DM, Gapin K, Horton I, Nguyen LL, et al. 2024. Regulation of human interferon signaling by transposon exonization. Cell. 187(26):7621-7636.e19. doi:10.1016/J.CELL.2024.11.016. [accessed 2026 Feb 3]. https://www.cell.com/action/showFullText?pii=S0092867424013333.

Payer LM, Steranka JP, Ardeljan D, Walker J, Fitzgerald KC, Calabresi PA, Cooper TA, Burns KH. 2018. Alu insertion variants alter mRNA splicing. Nucleic Acids Res. 47(1):421. doi:10.1093/NAR/GKY1086. [accessed 2026 Feb 3]. https://pmc.ncbi.nlm.nih.gov/articles/PMC6326789/.

Petri R, Brattås PL, Sharma Y, Jonsson ME, Pircs K, Bengzon J, Jakobsson J. 2019. LINE-2 transposable elements are a source of functional human microRNAs and target sites. PLoS Genet. 15(3):e1008036. doi:10.1371/JOURNAL.PGEN.1008036. [accessed 2026 Feb 2]. https://pmc.ncbi.nlm.nih.gov/articles/PMC6433296/.

Ranjan R, Logette E, Marani M, Herzog M, Tâche V, Scantamburlo E, Buchillier V, Markram H. 2019. A Kinetic Map of the Homomeric Voltage-Gated Potassium Channel (Kv) Family. Front Cell Neurosci. 13:450839. doi:10.3389/FNCEL.2019.00358/TEXT. [accessed 2026 Apr 3]. www.frontiersin.org.

Redaelli S, Grati FR, Tritto V, Giannuzzi G, Recalcati MP, Sala E, Villa N, Crosti F, Roversi G, Malvestiti F, et al. 2024. Olfactory receptor genes and chromosome 11 structural aberrations: Players or spectators? Human Genetics and Genomics Advances. 5(2). doi:10.1016/j.xhgg.2023.100261. [accessed 2026 Feb 3]. https://pubmed.ncbi.nlm.nih.gov/38160254/.

Roller M, Stamper E, Villar D, Izuogu O, Martin F, Redmond AM, Ramachanderan R, Harewood L, Odom DT, Flicek P. 2021. LINE retrotransposons characterize mammalian tissue-specific and evolutionarily dynamic regulatory regions. Genome Biology 2021 22:1. 22(1):62-. doi:10.1186/S13059-021-02260-Y. [accessed 2026 Feb 2]. https://link.springer.com/article/10.1186/s13059-021-02260-y.

Stamidis N, Żylicz JJ. 2023. RNA‐mediated heterochromatin formation at repetitive elements in mammals. EMBO J. 42(8):e111717. doi:10.15252/EMBJ.2022111717. [accessed 2026 Feb 2]. https://pmc.ncbi.nlm.nih.gov/articles/PMC10106986/.

Su M, Han D, Boyd-Kirkup J, Yu X, Han JDJ. 2014. Evolution of Alu Elements toward Enhancers. Cell Rep. 7(2):376–385. doi:10.1016/J.CELREP.2014.03.011. [accessed 2026 Feb 1]. https://www.sciencedirect.com/science/article/pii/S2211124714001892.

Suh A, Churakov G, Ramakodi MP, Platt RN, Jurka J, Kojima KK, Caballero J, Smit AF, Vliet KA, Hoffmann FG, et al. 2014. Multiple Lineages of Ancient CR1 Retroposons Shaped the Early Genome Evolution of Amniotes. Genome Biol Evol. 7(1):205. doi:10.1093/GBE/EVU256. [accessed 2026 Feb 2]. https://pmc.ncbi.nlm.nih.gov/articles/PMC4316615/.

Tunbak H, Enriquez-Gasca R, Tie CHC, Gould PA, Mlcochova P, Gupta RK, Fernandes L, Holt J, van der Veen AG, Giampazolias E, et al. 2020. The HUSH complex is a gatekeeper of type I interferon through epigenetic regulation of LINE-1s. Nature Communications 2020 11:1. 11(1):5387-. doi:10.1038/s41467-020-19170-5. [accessed 2026 Feb 3]. https://www.nature.com/articles/s41467-020-19170-5.

Urrutia J, Arrizabalaga-Iriondo A, Sanchez-del-Rey A, Martinez-Ibargüen A, Gallego M, Casis O, Revuelta M. 2024. Therapeutic role of voltage-gated potassium channels in age-related neurodegenerative diseases. Front Cell Neurosci. 18:1406709. doi:10.3389/FNCEL.2024.1406709/TEXT.

Wang Z, Ying Y, Wang M, Chen Q, Wang Y, Yu X, He W, Li J, Zeng S, Xu C. 2023. Comprehensive identification of onco-exaptation events in bladder cancer cell lines revealed L1PA2-SYT1 as a prognosis-relevant event. iScience. 26(12):108482. doi:10.1016/J.ISCI.2023.108482. [accessed 2026 Feb 2]. https://www.sciencedirect.com/science/article/pii/S2589004223025592.

Yu T, Yoon J, Zhu Y, Li A, Lee BJ, Moakley DF, Duan J, Deng Q, Hou F, Yan M, et al. 2025 Dec 18. Primate-specific adaptation of Ku protects transcriptomic integrity by suppressing Alu-mediated alternative splicing. bioRxiv. doi:10.64898/2025.12.17.694518. [accessed 2026 Apr 1]. https://pubmed.ncbi.nlm.nih.gov/41446183/.

Zottel A, Šamec N, Kump A, Dall’olio LR, Dominkuš PP, Romih R, Hudoklin S, Mlakar J, Nikitin D, Sorokin M, et al. 2020. Analysis of miR-9-5p, miR-124-3p, miR-21-5p, miR-138-5p, and miR-1-3p in Glioblastoma Cell Lines and Extracellular Vesicles. International Journal of Molecular Sciences 2020, Vol 21, Page 8491. 21(22):8491. doi:10.3390/IJMS21228491. [accessed 2026 Apr 5]. https://www.mdpi.com/1422-0067/21/22/8491/htm.
