# Supplementary material for: Telomere-to-telomere co-mapping of transposable elements and human genes identifies a cluster of young L1 elements in the interferon-alpha domain with potential evolutionary arms-race
Source: G3 (Bethesda). 2026 Sep 23;16(11):jkag250. doi: 10.1093/g3journal/jkag250 (PMC13732488; doi:10.1093/g3journal/jkag250)
Supplement: Supplementary file 1 [file jkag250_supplementary_data.zip › Supplementary_Figure_1.pdf]

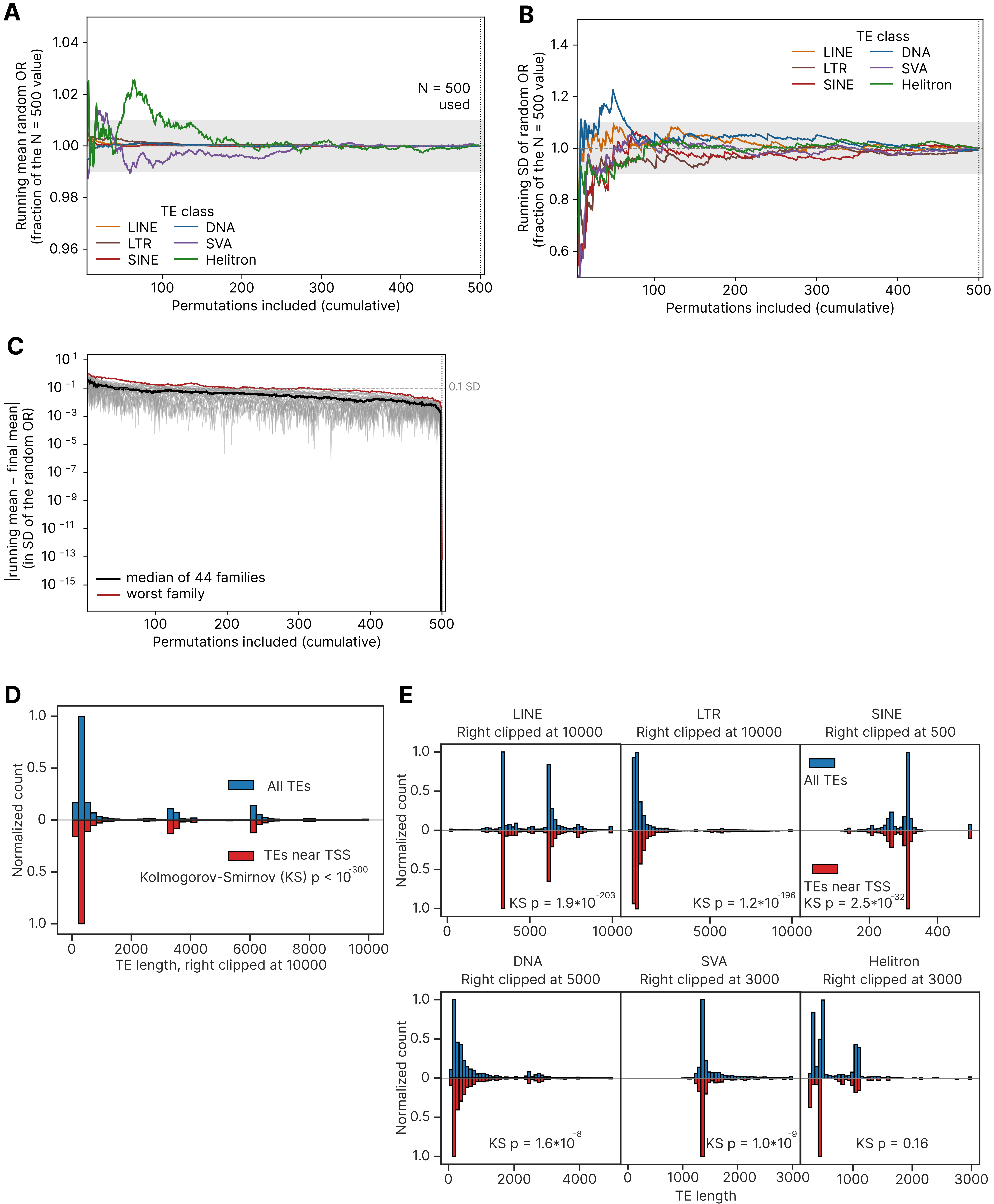

A
Running mean random OR
(fraction of the N = 500 value)
N = 500
used
TE class
LINE
LTR
SINE
DNA
SVA
Helitron
Permutations included (cumulative)
B
Running SD of random OR
(fraction of the N = 500 value)
TE class
LINE
LTR
SINE
DNA
SVA
Helitron
Permutations included (cumulative)
C
|running mean − final mean|
(in SD of the random OR)
0.1 SD
median of 44 families
worst family
Permutations included (cumulative)
D
Normalized count
All TEs
TEs near TSS
Kolmogorov-Smirnov (KS) p < 10^-300
TE length, right clipped at 10000
E
LINE
Right clipped at 10000
KS p = 1.9*10^-203
LTR
Right clipped at 10000
KS p = 1.2*10^-196
SINE
Right clipped at 500
All TEs
TEs near TSS
KS p = 2.5*10^-32
DNA
Right clipped at 5000
KS p = 1.6*10^-8
SVA
Right clipped at 3000
KS p = 1.0*10^-9
Helitron
Right clipped at 3000
KS p = 0.16
Normalized count
TE length
