# Supplementary material for: Telomere-to-telomere co-mapping of transposable elements and human genes identifies a cluster of young L1 elements in the interferon-alpha domain with potential evolutionary arms-race
Source: G3 (Bethesda). 2026 Sep 23;16(11):jkag250. doi: 10.1093/g3journal/jkag250 (PMC13732488; doi:10.1093/g3journal/jkag250)
Supplement: Supplementary file 1 [file jkag250_supplementary_data.zip › Supplementary_Figure_10.pdf]

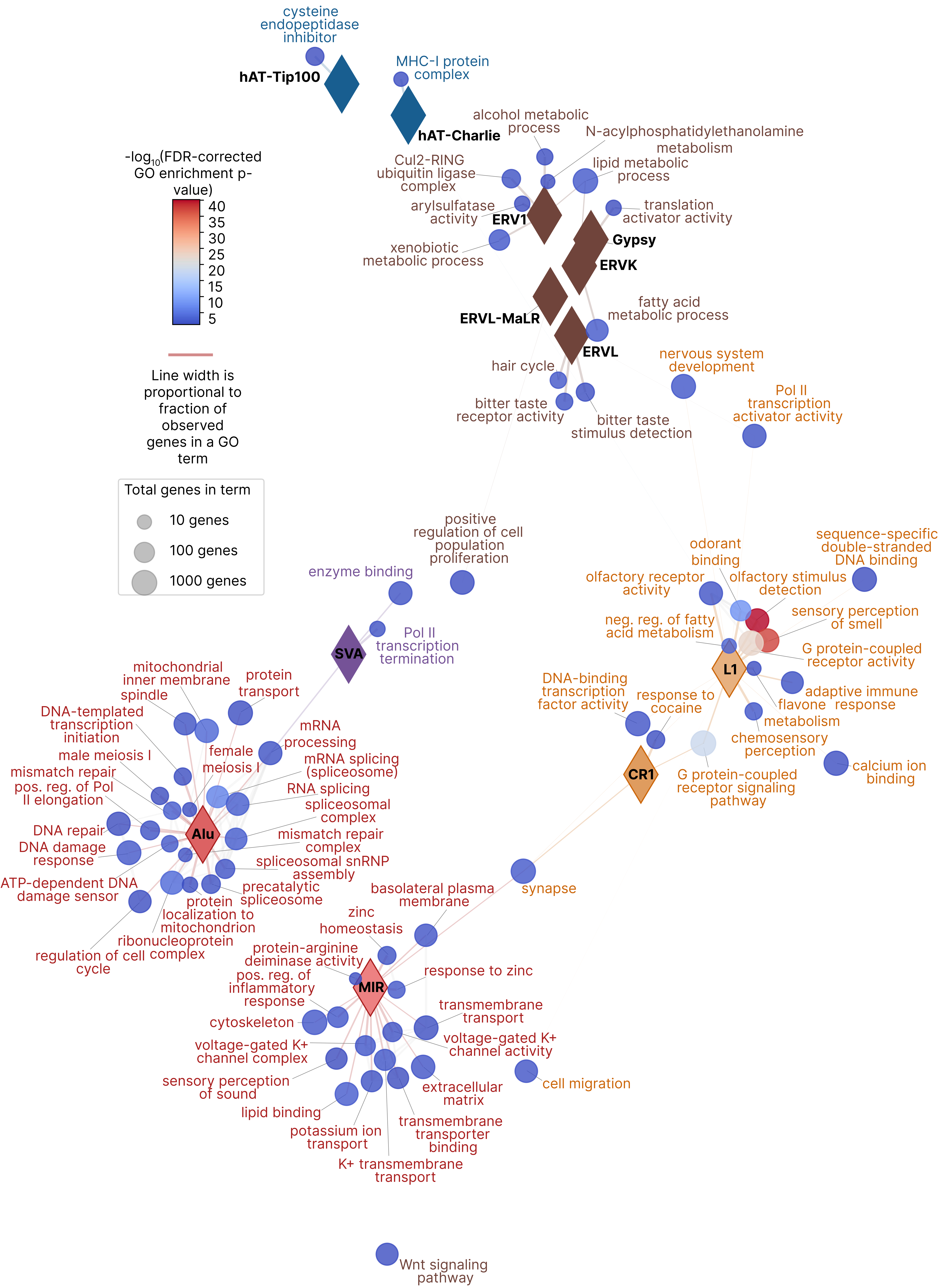

cysteine endopeptidase inhibitor
hAT-Tip100
MHC-I protein complex
hAT-Charlie
alcohol metabolic process
N-acylphosphatidylethanolamine metabolism
Cul2-RING ubiquitin ligase complex
lipid metabolic process
arylsulfatase activity
ERV1
translation activator activity
Gypsy
xenobiotic metabolic process
ERVK
fatty acid metabolic process
ERVL-MaLR
ERVL
hair cycle
nervous system development
Pol II transcription activator activity
bitter taste receptor activity
bitter taste stimulus detection
-log10(FDR-corrected GO enrichment p-value)
40
35
30
25
20
15
10
5
Line width is proportional to fraction of observed genes in a GO term
Total genes in term
10 genes
100 genes
1000 genes
positive regulation of cell population proliferation
enzyme binding
odorant binding
sequence-specific double-stranded DNA binding
olfactory receptor activity
olfactory stimulus detection
Pol II transcription termination
SVA
neg. reg. of fatty acid metabolism
sensory perception of smell
G protein-coupled receptor activity
L1
mitochondrial inner membrane
spindle
protein transport
DNA-binding transcription factor activity
response to cocaine
adaptive immune response
flavone metabolism
DNA-templated transcription initiation
mRNA processing
chemosensory perception
male meiosis I
female meiosis I
mRNA splicing (spliceosome)
CR1
calcium ion binding
mismatch repair
pos. reg. of Pol II elongation
RNA splicing
G protein-coupled receptor signaling pathway
spliceosomal complex
DNA repair
Alu
mismatch repair complex
DNA damage response
spliceosomal snRNP assembly
ATP-dependent DNA damage sensor
precatalytic spliceosome
synapse
basolateral plasma membrane
protein localization to mitochondrion
zinc homeostasis
ribonucleoprotein complex
regulation of cell cycle
protein-arginine deiminase activity
pos. reg. of inflammatory response
MIR
response to zinc
transmembrane transport
cytoskeleton
voltage-gated K+ channel complex
voltage-gated K+ channel activity
sensory perception of sound
cell migration
extracellular matrix
lipid binding
potassium ion transport
transmembrane transporter binding
K+ transmembrane transport
Wnt signaling pathway
