# Supplementary material for: Telomere-to-telomere co-mapping of transposable elements and human genes identifies a cluster of young L1 elements in the interferon-alpha domain with potential evolutionary arms-race
Source: G3 (Bethesda). 2026 Sep 23;16(11):jkag250. doi: 10.1093/g3journal/jkag250 (PMC13732488; doi:10.1093/g3journal/jkag250)
Supplement: Supplementary file 1 [file jkag250_supplementary_data.zip › Supplementary_Figure_11.pdf]

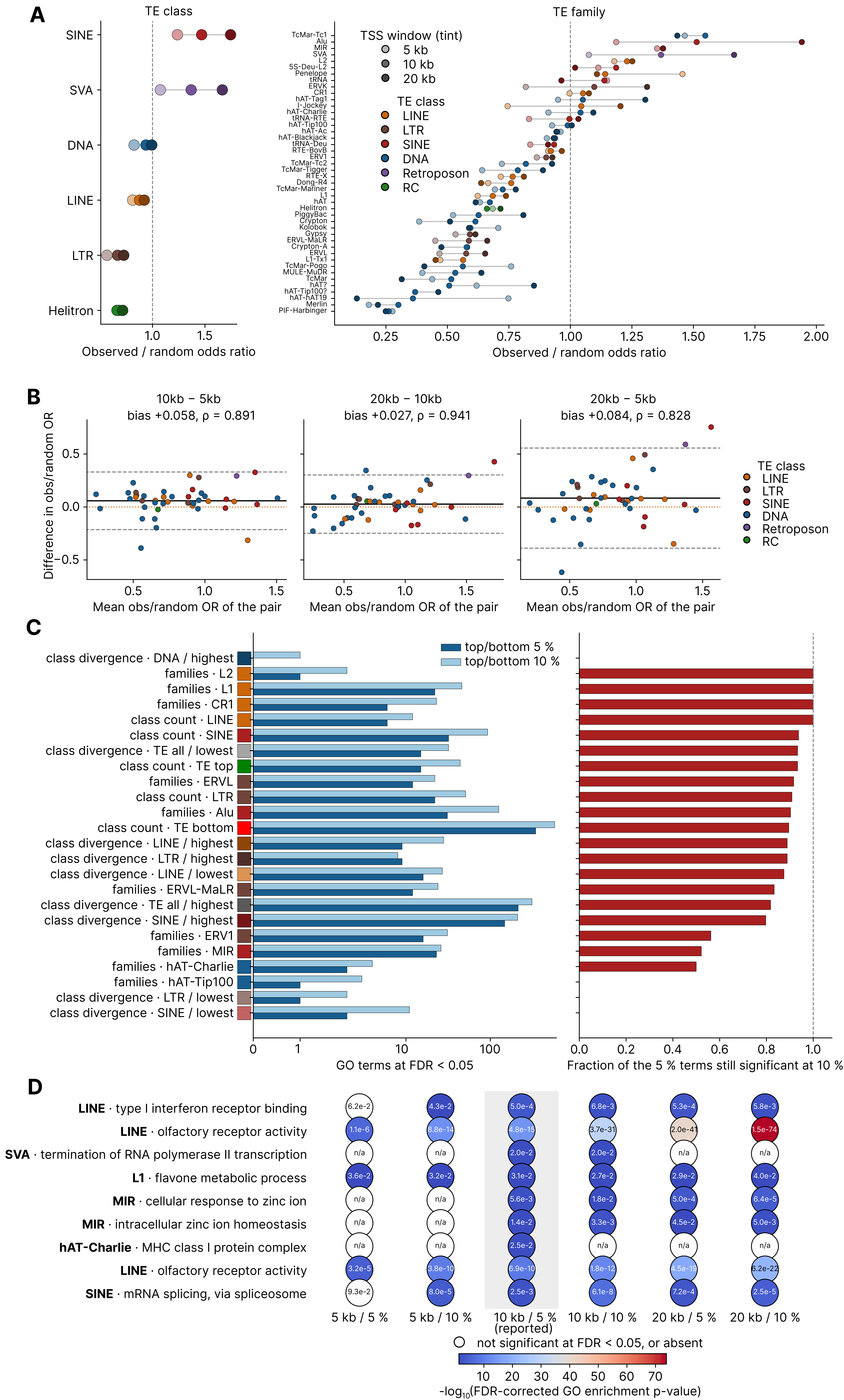

A
TE class
SINE
SVA
DNA
LINE
LTR
Helitron
1.0
1.5
Observed / random odds ratio
TE family
TSS window (tint)
5 kb
10 kb
20 kb
TE class
LINE
LTR
SINE
DNA
Retroposon
RC
TcMar-Tc1
Alu
MIR
SVA
L2
5S-Deu-L2
Penelope
tRNA
ERVK
CR1
hAT-Tag1
I-Jockey
hAT-Charlie
tRNA-RTE
hAT-Tip100
hAT-Ac
hAT-Blackjack
tRNA-Deu
RTE-BovB
ERV1
TcMar-Tc2
TcMar-Tigger
RTE-X
Dong-R4
TcMar-Mariner
L1
hAT
Helitron
PiggyBac
Crypton
Kolobok
Gypsy
ERVL-MaLR
Crypton-A
ERVL
L1-Tx1
TcMar-Pogo
MULE-MuDR
TcMar
hAT?
hAT-Tip100?
hAT-hAT19
Merlin
PIF-Harbinger
0.25
0.50
0.75
1.00
1.25
1.50
1.75
2.00
Observed / random odds ratio
B
10kb − 5kb
bias +0.058, ρ = 0.891
20kb − 10kb
bias +0.027, ρ = 0.941
20kb − 5kb
bias +0.084, ρ = 0.828
Difference in obs/random OR
0.5
0.0
−0.5
Mean obs/random OR of the pair
TE class
LINE
LTR
SINE
DNA
Retroposon
RC
C
class divergence · DNA / highest
families · L2
families · L1
families · CR1
class count · LINE
class count · SINE
class divergence · TE all / lowest
class count · TE top
families · ERVL
class count · LTR
families · Alu
class count · TE bottom
class divergence · LINE / highest
class divergence · LTR / highest
class divergence · LINE / lowest
families · ERVL-MaLR
class divergence · TE all / highest
class divergence · SINE / highest
families · ERV1
families · MIR
families · hAT-Charlie
families · hAT-Tip100
class divergence · LTR / lowest
class divergence · SINE / lowest
top/bottom 5 %
top/bottom 10 %
0
1
10
100
GO terms at FDR < 0.05
0.0
0.2
0.4
0.6
0.8
1.0
Fraction of the 5 % terms still significant at 10 %
D
LINE · type I interferon receptor binding
LINE · olfactory receptor activity
SVA · termination of RNA polymerase II transcription
L1 · flavone metabolic process
MIR · cellular response to zinc ion
MIR · intracellular zinc ion homeostasis
hAT-Charlie · MHC class I protein complex
LINE · olfactory receptor activity
SINE · mRNA splicing, via spliceosome
6.2e-2 4.3e-2 5.0e-4 6.8e-3 5.3e-4 5.8e-3
1.1e-6 8.8e-14 4.8e-15 3.7e-31 2.0e-41 1.5e-74
n/a n/a 2.0e-2 2.0e-2 n/a n/a
3.6e-2 3.2e-2 3.1e-2 2.7e-2 2.9e-2 4.0e-2
n/a n/a 5.6e-3 1.8e-2 5.0e-4 6.4e-5
n/a n/a 1.4e-2 3.3e-3 4.5e-2 5.0e-3
n/a n/a 2.5e-2 n/a n/a n/a
3.2e-5 3.8e-10 6.9e-10 1.8e-12 4.5e-19 6.2e-22
9.3e-2 8.0e-5 2.5e-3 6.1e-8 7.2e-4 2.5e-5
5 kb / 5 %
5 kb / 10 %
10 kb / 5 %
(reported)
10 kb / 10 %
20 kb / 5 %
20 kb / 10 %
not significant at FDR < 0.05, or absent
10 20 30 40 50 60 70
-log10(FDR-corrected GO enrichment p-value)
