# Supplementary material for: Telomere-to-telomere co-mapping of transposable elements and human genes identifies a cluster of young L1 elements in the interferon-alpha domain with potential evolutionary arms-race
Source: G3 (Bethesda). 2026 Sep 23;16(11):jkag250. doi: 10.1093/g3journal/jkag250 (PMC13732488; doi:10.1093/g3journal/jkag250)
Supplement: Supplementary file 1 [file jkag250_supplementary_data.zip › Supplementary_Figure_12.pdf]

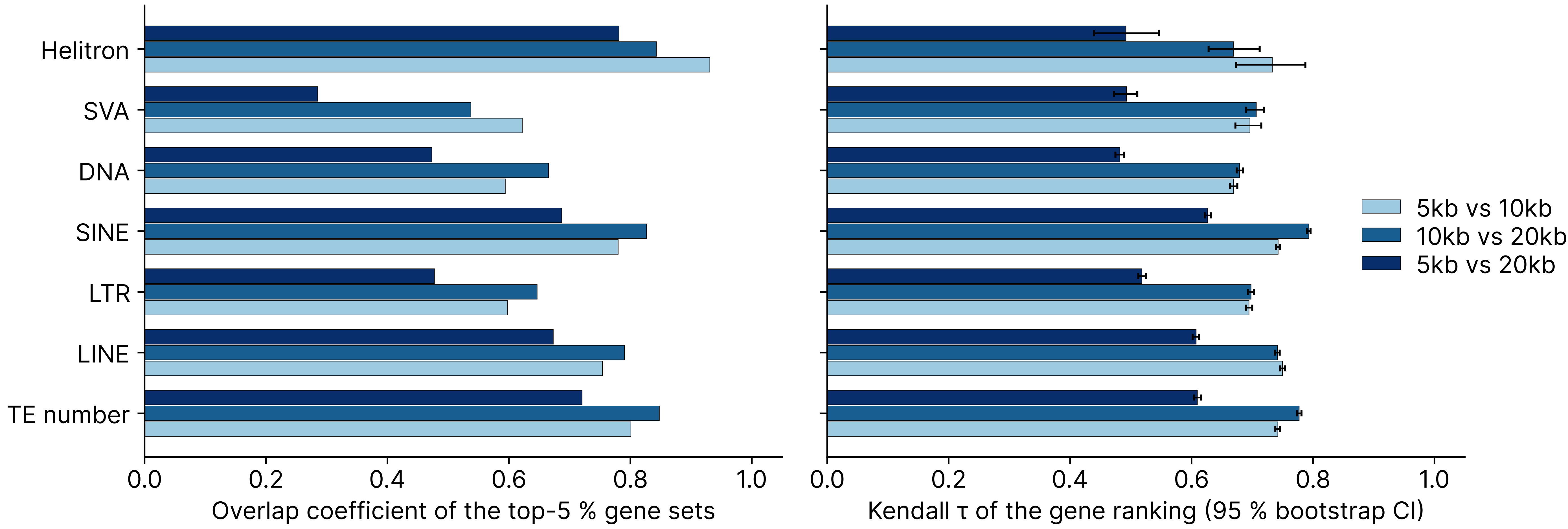

Helitron
SVA
DNA
SINE
LTR
LINE
TE number
0.0
0.2
0.4
0.6
0.8
1.0
Overlap coefficient of the top-5 % gene sets
Kendall τ of the gene ranking (95 % bootstrap CI)
5kb vs 10kb
10kb vs 20kb
5kb vs 20kb
