# Supplementary material for: Telomere-to-telomere co-mapping of transposable elements and human genes identifies a cluster of young L1 elements in the interferon-alpha domain with potential evolutionary arms-race
Source: G3 (Bethesda). 2026 Sep 23;16(11):jkag250. doi: 10.1093/g3journal/jkag250 (PMC13732488; doi:10.1093/g3journal/jkag250)
Supplement: Supplementary file 1 [file jkag250_supplementary_data.zip › Supplementary_Figure_13.pdf]

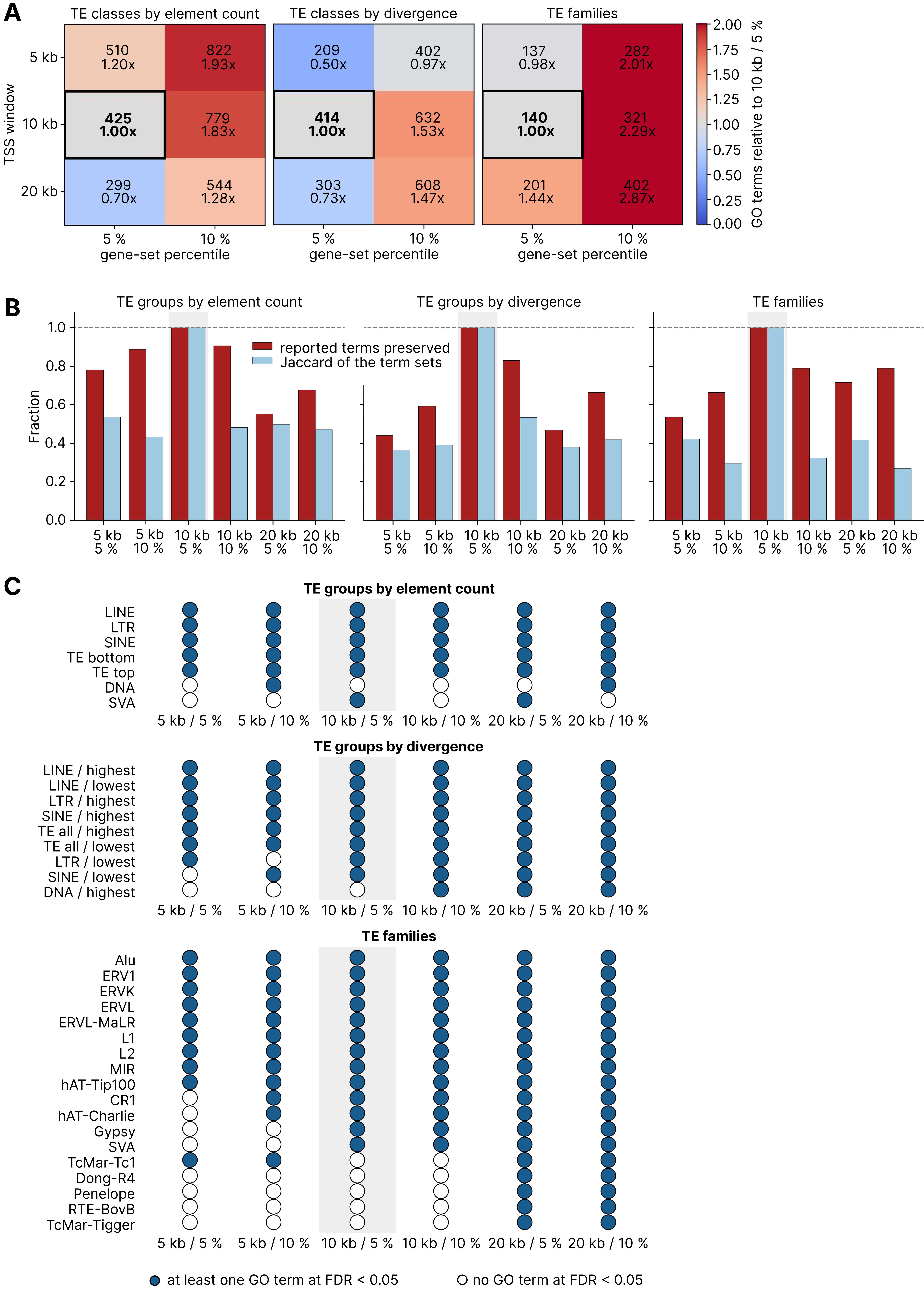

A
TE classes by element count
TE classes by divergence
TE families
TSS window
5 kb
10 kb
20 kb
510
1.20x
822
1.93x
425
1.00x
779
1.83x
299
0.70x
544
1.28x
209
0.50x
402
0.97x
414
1.00x
632
1.53x
303
0.73x
608
1.47x
137
0.98x
282
2.01x
140
1.00x
321
2.29x
201
1.44x
402
2.87x
5 %
10 %
gene-set percentile
GO terms relative to 10 kb / 5 %
2.00
1.75
1.50
1.25
1.00
0.75
0.50
0.25
0.00
B
TE groups by element count
TE groups by divergence
TE families
Fraction
reported terms preserved
Jaccard of the term sets
1.0
0.8
0.6
0.4
0.2
0.0
5 kb 5 %
5 kb 10 %
10 kb 5 %
10 kb 10 %
20 kb 5 %
20 kb 10 %
C
TE groups by element count
LINE
LTR
SINE
TE bottom
TE top
DNA
SVA
5 kb / 5 %
5 kb / 10 %
10 kb / 5 %
10 kb / 10 %
20 kb / 5 %
20 kb / 10 %
TE groups by divergence
LINE / highest
LINE / lowest
LTR / highest
SINE / highest
TE all / highest
TE all / lowest
LTR / lowest
SINE / lowest
DNA / highest
TE families
Alu
ERV1
ERVK
ERVL
ERVL-MaLR
L1
L2
MIR
hAT-Tip100
CR1
hAT-Charlie
Gypsy
SVA
TcMar-Tc1
Dong-R4
Penelope
RTE-BovB
TcMar-Tigger
at least one GO term at FDR < 0.05
no GO term at FDR < 0.05
