# Supplementary material for: Telomere-to-telomere co-mapping of transposable elements and human genes identifies a cluster of young L1 elements in the interferon-alpha domain with potential evolutionary arms-race
Source: G3 (Bethesda). 2026 Sep 23;16(11):jkag250. doi: 10.1093/g3journal/jkag250 (PMC13732488; doi:10.1093/g3journal/jkag250)
Supplement: Supplementary file 1 [file jkag250_supplementary_data.zip › Supplementary_Figure_3.pdf]

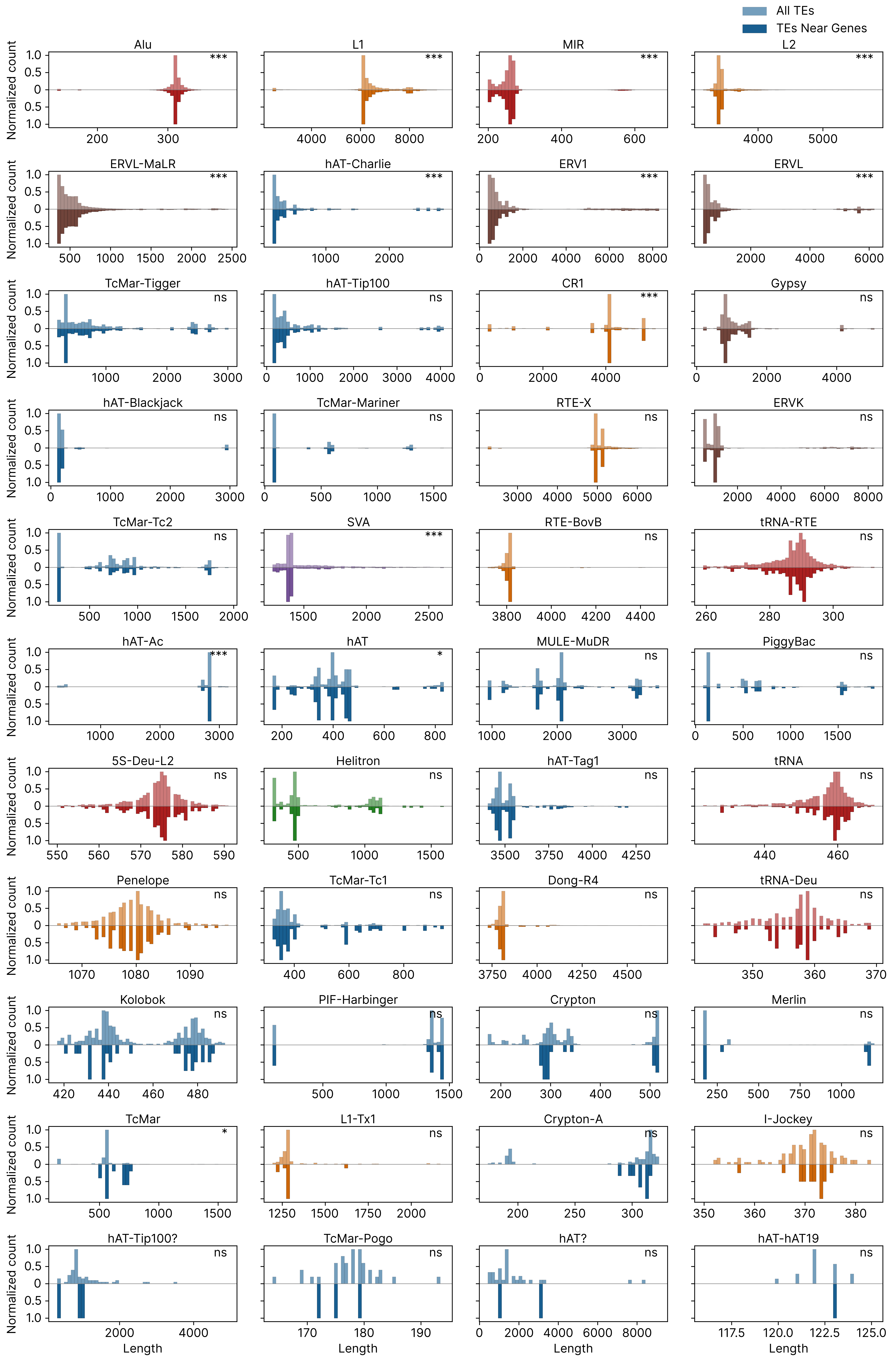

All TEs
TEs Near Genes
Normalized count
Alu ***
L1 ***
MIR ***
L2 ***
ERVL-MaLR ***
hAT-Charlie ***
ERV1 ***
ERVL ***
TcMar-Tigger ns
hAT-Tip100 ns
CR1 ***
Gypsy ns
hAT-Blackjack ns
TcMar-Mariner ns
RTE-X ns
ERVK ns
TcMar-Tc2 ns
SVA ***
RTE-BovB ns
tRNA-RTE ns
hAT-Ac ***
hAT *
MULE-MuDR ns
PiggyBac ns
5S-Deu-L2 ns
Helitron ns
hAT-Tag1 ns
tRNA ns
Penelope ns
TcMar-Tc1 ns
Dong-R4 ns
tRNA-Deu ns
Kolobok ns
PIF-Harbinger ns
Crypton ns
Merlin ns
TcMar *
L1-Tx1 ns
Crypton-A ns
I-Jockey ns
hAT-Tip100? ns
TcMar-Pogo ns
hAT? ns
hAT-hAT19 ns
Length
