# Supplementary material for: Telomere-to-telomere co-mapping of transposable elements and human genes identifies a cluster of young L1 elements in the interferon-alpha domain with potential evolutionary arms-race
Source: G3 (Bethesda). 2026 Sep 23;16(11):jkag250. doi: 10.1093/g3journal/jkag250 (PMC13732488; doi:10.1093/g3journal/jkag250)
Supplement: Supplementary file 1 [file jkag250_supplementary_data.zip › Supplementary_Figure_4.pdf]

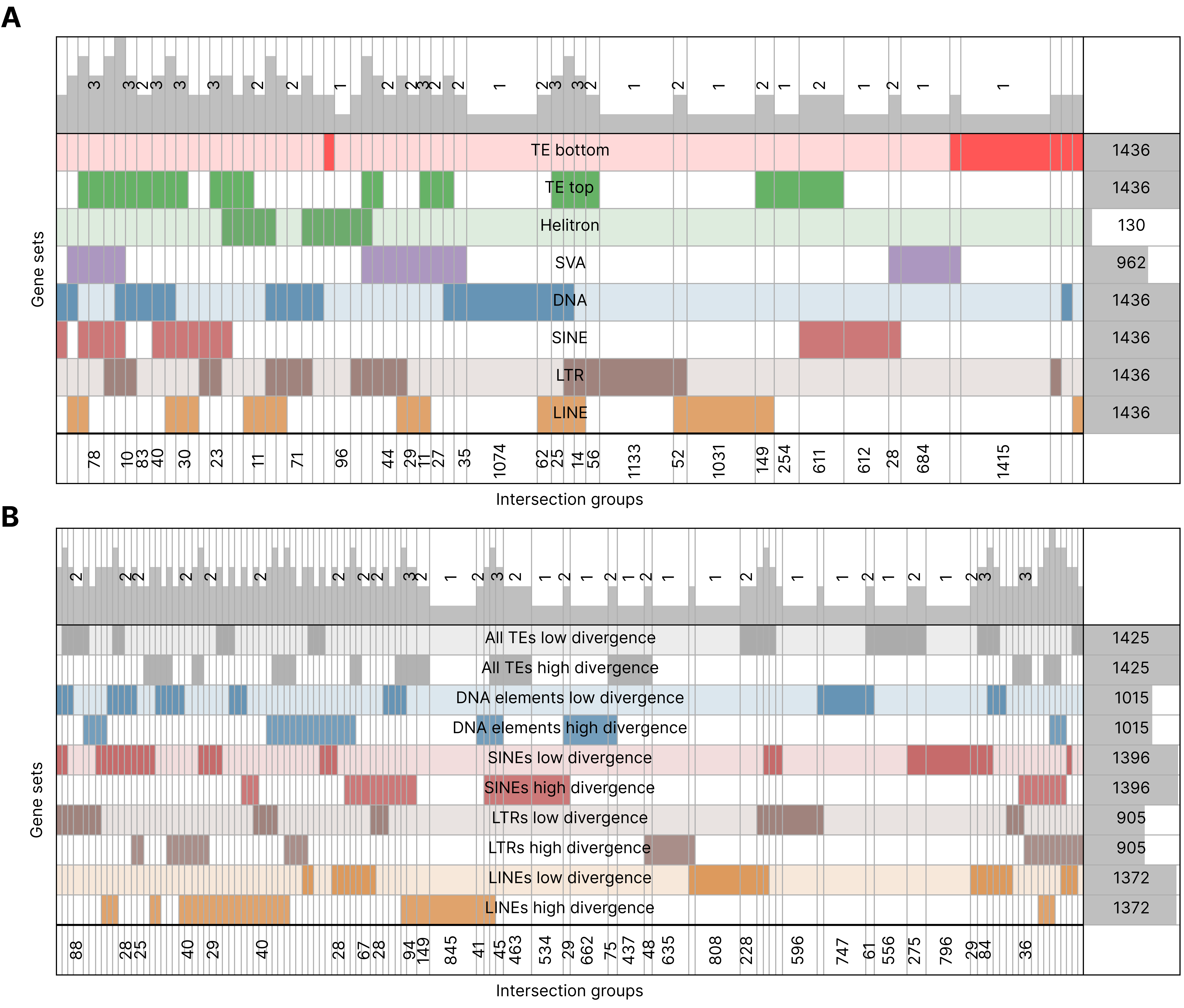
A
TE bottom
TE top
Helitron
SVA
DNA
SINE
LTR
LINE
1436
1436
130
962
1436
1436
1436
1436
Gene sets
Intersection groups
B
All TEs low divergence
All TEs high divergence
DNA elements low divergence
DNA elements high divergence
SINEs low divergence
SINEs high divergence
LTRs low divergence
LTRs high divergence
LINEs low divergence
LINEs high divergence
1425
1425
1015
1015
1396
1396
905
905
1372
1372
Gene sets
Intersection groups
