# Supplementary material for: Telomere-to-telomere co-mapping of transposable elements and human genes identifies a cluster of young L1 elements in the interferon-alpha domain with potential evolutionary arms-race
Source: G3 (Bethesda). 2026 Sep 23;16(11):jkag250. doi: 10.1093/g3journal/jkag250 (PMC13732488; doi:10.1093/g3journal/jkag250)
Supplement: Supplementary file 1 [file jkag250_supplementary_data.zip › Supplementary_Figure_5.pdf]

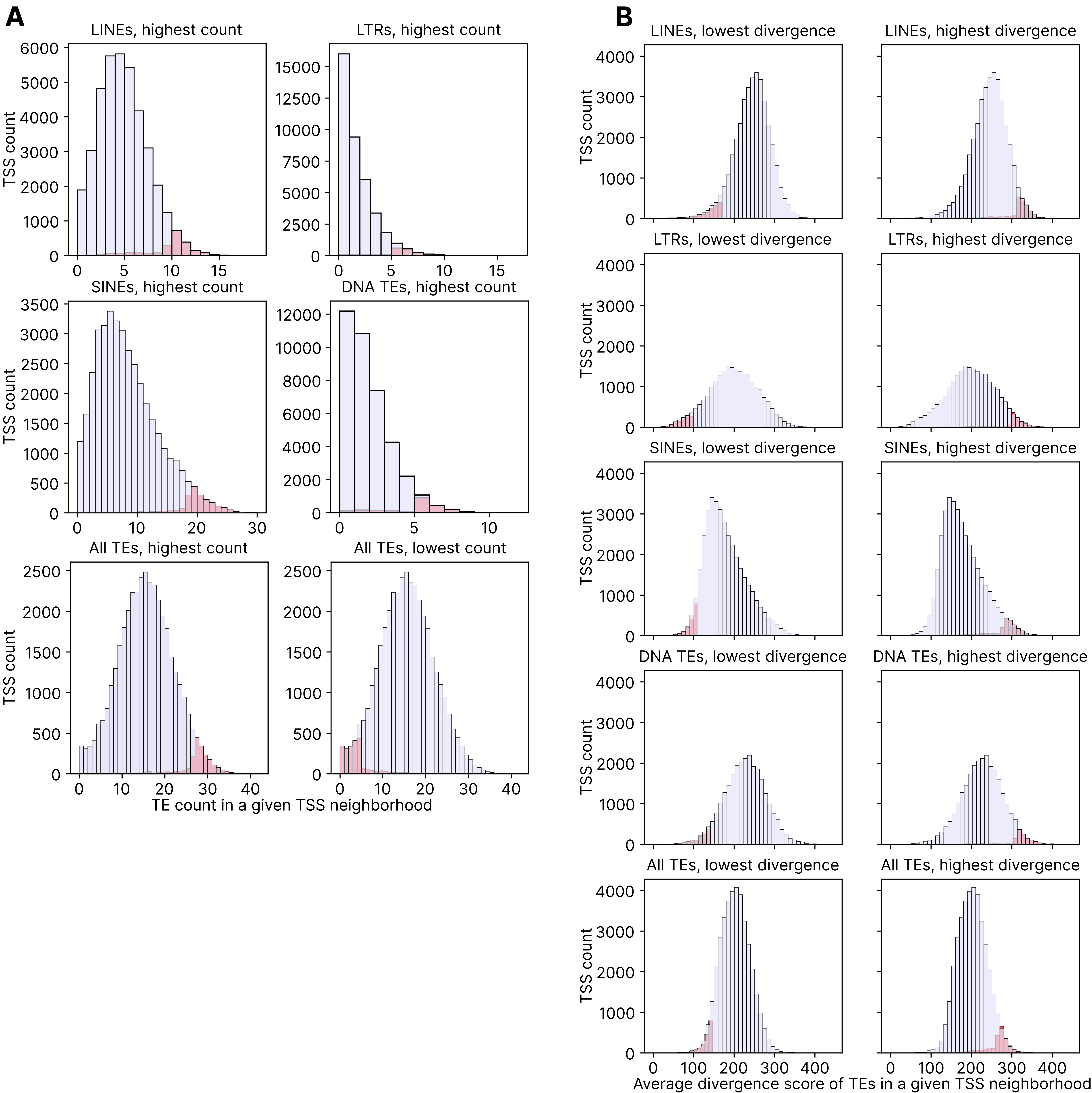
A
LINEs, highest count
LTRs, highest count
SINEs, highest count
DNA TEs, highest count
All TEs, highest count
All TEs, lowest count
TSS count
TE count in a given TSS neighborhood
B
LINEs, lowest divergence
LINEs, highest divergence
LTRs, lowest divergence
LTRs, highest divergence
SINEs, lowest divergence
SINEs, highest divergence
DNA TEs, lowest divergence
DNA TEs, highest divergence
All TEs, lowest divergence
All TEs, highest divergence
Average divergence score of TEs in a given TSS neighborhood
