# Supplementary material for: Telomere-to-telomere co-mapping of transposable elements and human genes identifies a cluster of young L1 elements in the interferon-alpha domain with potential evolutionary arms-race
Source: G3 (Bethesda). 2026 Sep 23;16(11):jkag250. doi: 10.1093/g3journal/jkag250 (PMC13732488; doi:10.1093/g3journal/jkag250)
Supplement: Supplementary file 1 [file jkag250_supplementary_data.zip › Supplementary_Figure_6.pdf]

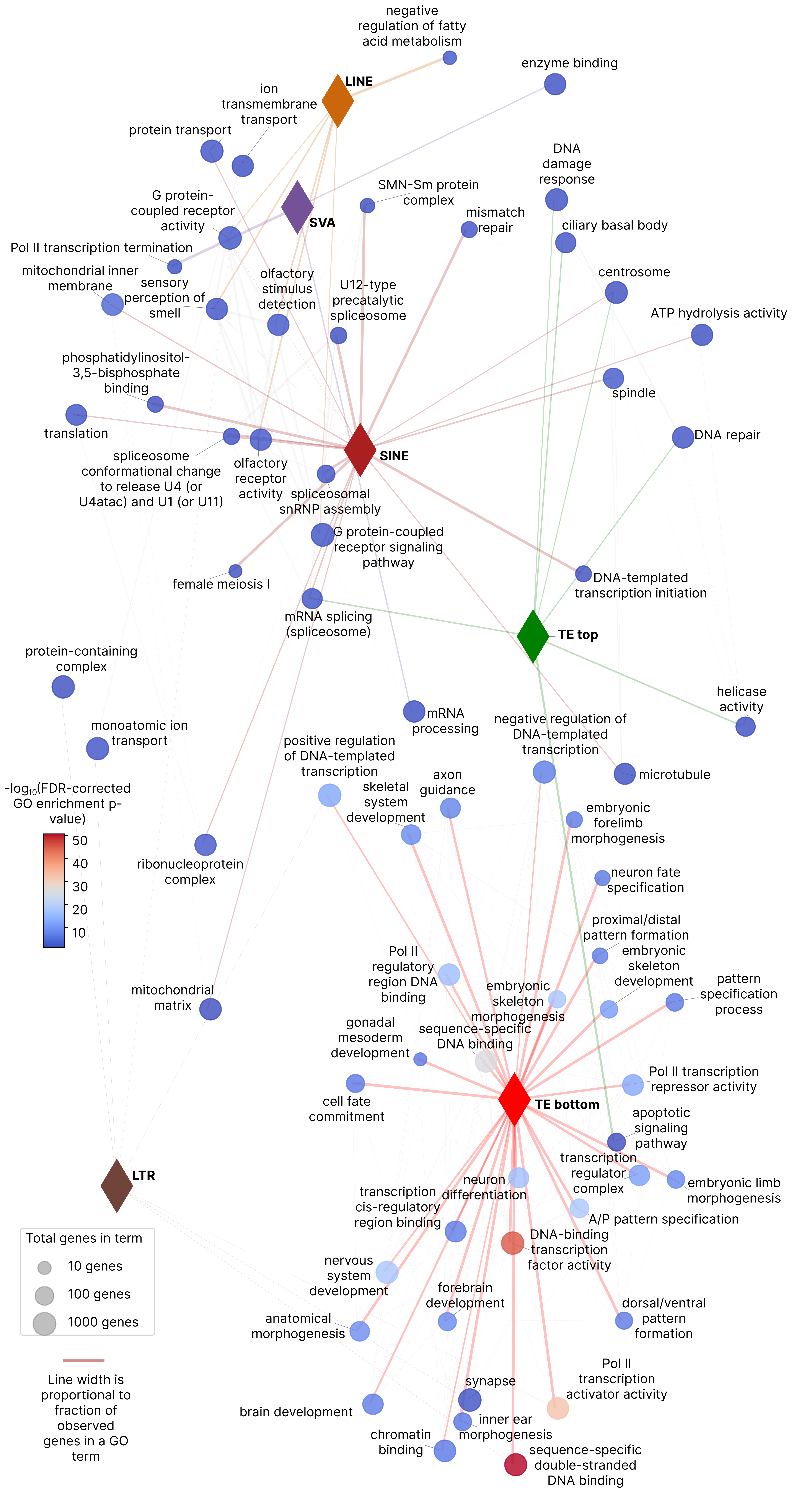

negative regulation of fatty acid metabolism
LINE
enzyme binding
ion transmembrane transport
protein transport
DNA damage response
SMN-Sm protein complex
mismatch repair
SVA
G protein-coupled receptor activity
ciliary basal body
Pol II transcription termination
mitochondrial inner membrane
sensory perception of smell
olfactory stimulus detection
U12-type precatalytic spliceosome
centrosome
ATP hydrolysis activity
phosphatidylinositol-3,5-bisphosphate binding
spindle
translation
DNA repair
SINE
spliceosome conformational change to release U4 (or U4atac) and U1 (or U11)
olfactory receptor activity
spliceosomal snRNP assembly
G protein-coupled receptor signaling pathway
DNA-templated transcription initiation
female meiosis I
mRNA splicing (spliceosome)
TE top
protein-containing complex
helicase activity
mRNA processing
negative regulation of DNA-templated transcription
monoatomic ion transport
microtubule
positive regulation of DNA-templated transcription
-log10(FDR-corrected GO enrichment p-value)
50
40
30
20
10
skeletal system development
axon guidance
embryonic forelimb morphogenesis
ribonucleoprotein complex
neuron fate specification
proximal/distal pattern formation
embryonic skeleton development
Pol II regulatory region DNA binding
pattern specification process
mitochondrial matrix
embryonic skeleton morphogenesis
gonadal mesoderm development
sequence-specific DNA binding
Pol II transcription repressor activity
cell fate commitment
TE bottom
apoptotic signaling pathway
LTR
transcription regulator complex
embryonic limb morphogenesis
transcription cis-regulatory region binding
neuron differentiation
A/P pattern specification
Total genes in term
10 genes
100 genes
1000 genes
DNA-binding transcription factor activity
nervous system development
forebrain development
anatomical morphogenesis
dorsal/ventral pattern formation
Line width is proportional to fraction of observed genes in a GO term
Pol II transcription activator activity
synapse
brain development
inner ear morphogenesis
chromatin binding
sequence-specific double-stranded DNA binding
