# Supplementary material for: Telomere-to-telomere co-mapping of transposable elements and human genes identifies a cluster of young L1 elements in the interferon-alpha domain with potential evolutionary arms-race
Source: G3 (Bethesda). 2026 Sep 23;16(11):jkag250. doi: 10.1093/g3journal/jkag250 (PMC13732488; doi:10.1093/g3journal/jkag250)
Supplement: Supplementary file 1 [file jkag250_supplementary_data.zip › Supplementary_Figure_7.pdf]

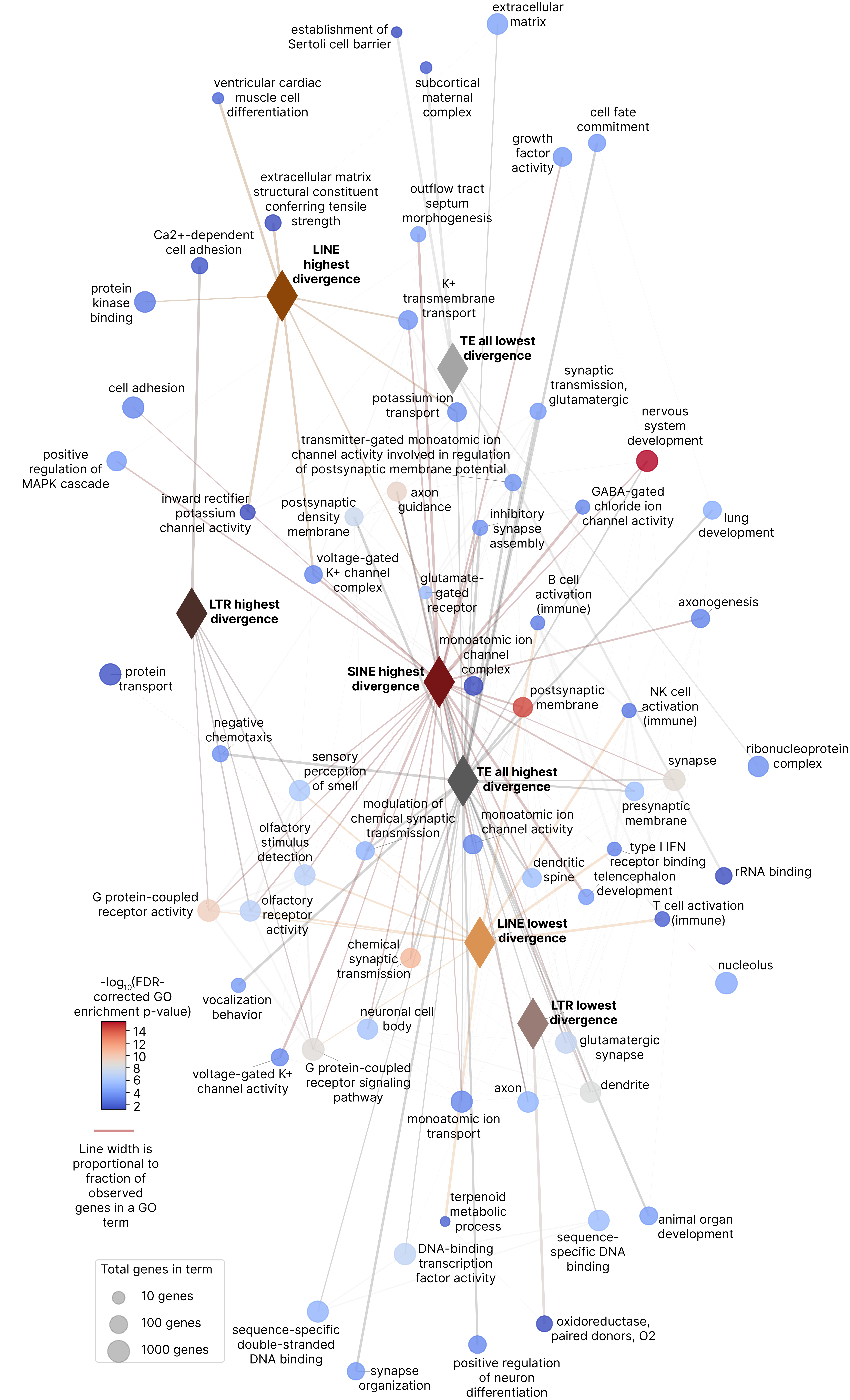
extracellular matrix
establishment of Sertoli cell barrier
subcortical maternal complex
ventricular cardiac muscle cell differentiation
cell fate commitment
growth factor activity
extracellular matrix structural constituent conferring tensile strength
outflow tract septum morphogenesis
Ca2+-dependent cell adhesion
LINE highest divergence
protein kinase binding
K+ transmembrane transport
TE all lowest divergence
cell adhesion
potassium ion transport
synaptic transmission, glutamatergic
nervous system development
positive regulation of MAPK cascade
transmitter-gated monoatomic ion channel activity involved in regulation of postsynaptic membrane potential
axon guidance
inward rectifier potassium channel activity
postsynaptic density membrane
GABA-gated chloride ion channel activity
inhibitory synapse assembly
lung development
voltage-gated K+ channel complex
glutamate-gated receptor
B cell activation (immune)
LTR highest divergence
axonogenesis
monoatomic ion channel complex
SINE highest divergence
protein transport
postsynaptic membrane
NK cell activation (immune)
negative chemotaxis
sensory perception of smell
TE all highest divergence
synapse
ribonucleoprotein complex
presynaptic membrane
modulation of chemical synaptic transmission
monoatomic ion channel activity
olfactory stimulus detection
type I IFN receptor binding
rRNA binding
dendritic spine
telencephalon development
G protein-coupled receptor activity
olfactory receptor activity
T cell activation (immune)
LINE lowest divergence
chemical synaptic transmission
nucleolus
-log10(FDR-corrected GO enrichment p-value)
14
12
10
8
6
4
2
vocalization behavior
neuronal cell body
LTR lowest divergence
glutamatergic synapse
voltage-gated K+ channel activity
G protein-coupled receptor signaling pathway
axon
dendrite
monoatomic ion transport
Line width is proportional to fraction of observed genes in a GO term
terpenoid metabolic process
animal organ development
sequence-specific DNA binding
DNA-binding transcription factor activity
Total genes in term
10 genes
100 genes
1000 genes
sequence-specific double-stranded DNA binding
oxidoreductase, paired donors, O2
positive regulation of neuron differentiation
synapse organization
