# Supplementary material for: Telomere-to-telomere co-mapping of transposable elements and human genes identifies a cluster of young L1 elements in the interferon-alpha domain with potential evolutionary arms-race
Source: G3 (Bethesda). 2026 Sep 23;16(11):jkag250. doi: 10.1093/g3journal/jkag250 (PMC13732488; doi:10.1093/g3journal/jkag250)
Supplement: Supplementary file 1 [file jkag250_supplementary_data.zip › Supplementary_Figure_8.pdf]

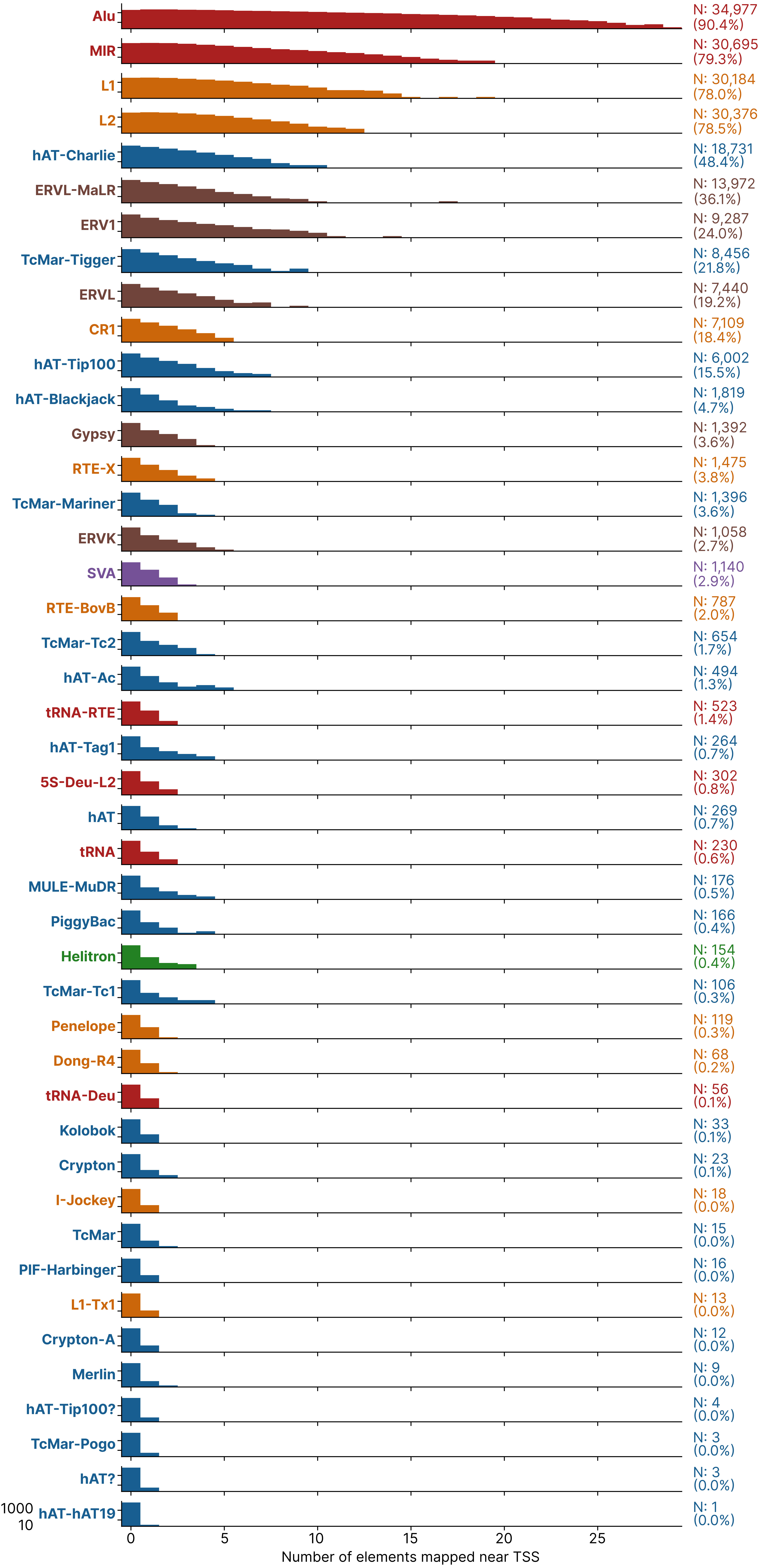
Alu
N: 34,977 (90.4%)
MIR
N: 30,695 (79.3%)
L1
N: 30,184 (78.0%)
L2
N: 30,376 (78.5%)
hAT-Charlie
N: 18,731 (48.4%)
ERVL-MaLR
N: 13,972 (36.1%)
ERV1
N: 9,287 (24.0%)
TcMar-Tigger
N: 8,456 (21.8%)
ERVL
N: 7,440 (19.2%)
CR1
N: 7,109 (18.4%)
hAT-Tip100
N: 6,002 (15.5%)
hAT-Blackjack
N: 1,819 (4.7%)
Gypsy
N: 1,392 (3.6%)
RTE-X
N: 1,475 (3.8%)
TcMar-Mariner
N: 1,396 (3.6%)
ERVK
N: 1,058 (2.7%)
SVA
N: 1,140 (2.9%)
RTE-BovB
N: 787 (2.0%)
TcMar-Tc2
N: 654 (1.7%)
hAT-Ac
N: 494 (1.3%)
tRNA-RTE
N: 523 (1.4%)
hAT-Tag1
N: 264 (0.7%)
5S-Deu-L2
N: 302 (0.8%)
hAT
N: 269 (0.7%)
tRNA
N: 230 (0.6%)
MULE-MuDR
N: 176 (0.5%)
PiggyBac
N: 166 (0.4%)
Helitron
N: 154 (0.4%)
TcMar-Tc1
N: 106 (0.3%)
Penelope
N: 119 (0.3%)
Dong-R4
N: 68 (0.2%)
tRNA-Deu
N: 56 (0.1%)
Kolobok
N: 33 (0.1%)
Crypton
N: 23 (0.1%)
I-Jockey
N: 18 (0.0%)
TcMar
N: 15 (0.0%)
PIF-Harbinger
N: 16 (0.0%)
L1-Tx1
N: 13 (0.0%)
Crypton-A
N: 12 (0.0%)
Merlin
N: 9 (0.0%)
hAT-Tip100?
N: 4 (0.0%)
TcMar-Pogo
N: 3 (0.0%)
hAT?
N: 3 (0.0%)
1000
10
hAT-hAT19
N: 1 (0.0%)
0
5
10
15
20
25
Number of elements mapped near TSS
