# Supplementary material for: Telomere-to-telomere co-mapping of transposable elements and human genes identifies a cluster of young L1 elements in the interferon-alpha domain with potential evolutionary arms-race
Source: G3 (Bethesda). 2026 Sep 23;16(11):jkag250. doi: 10.1093/g3journal/jkag250 (PMC13732488; doi:10.1093/g3journal/jkag250)
Supplement: Supplementary file 1 [file jkag250_supplementary_data.zip › Supplementary_Figure_9.pdf]

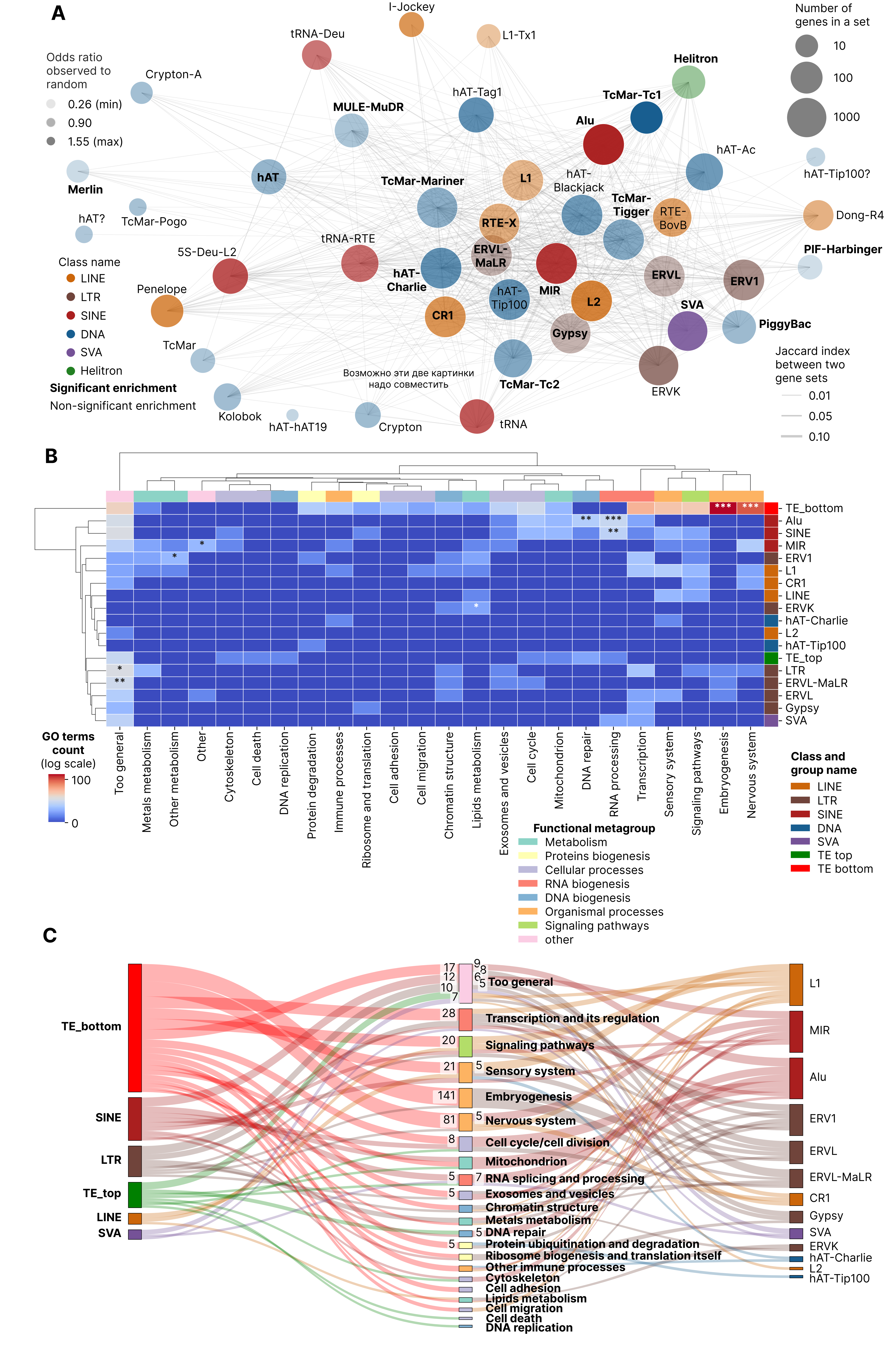
A
Odds ratio observed to random
0.26 (min)
0.90
1.55 (max)
Class name
LINE
LTR
SINE
DNA
SVA
Helitron
Significant enrichment
Non-significant enrichment
Number of genes in a set
10
100
1000
Jaccard index between two gene sets
0.01
0.05
0.10
I-Jockey
L1-Tx1
tRNA-Deu
Crypton-A
Helitron
TcMar-Tc1
hAT-Tag1
MULE-MuDR
Alu
hAT-Ac
hAT-Tip100?
Merlin
hAT
TcMar-Mariner
L1
hAT-Blackjack
TcMar-Tigger
RTE-BovB
Dong-R4
hAT?
TcMar-Pogo
RTE-X
tRNA-RTE
5S-Deu-L2
ERVL-MaLR
PIF-Harbinger
hAT-Charlie
MIR
ERVL
ERV1
Penelope
hAT-Tip100
L2
CR1
SVA
PiggyBac
Gypsy
TcMar
Возможно эти две картинки надо совместить
TcMar-Tc2
ERVK
Kolobok
hAT-hAT19
Crypton
tRNA
B
GO terms count (log scale)
100
0
TE_bottom
Alu
SINE
MIR
ERV1
L1
CR1
LINE
ERVK
hAT-Charlie
L2
hAT-Tip100
TE_top
LTR
ERVL-MaLR
ERVL
Gypsy
SVA
***
**
*
Too general
Metals metabolism
Other metabolism
Other
Cytoskeleton
Cell death
DNA replication
Protein degradation
Immune processes
Ribosome and translation
Cell adhesion
Cell migration
Chromatin structure
Lipids metabolism
Exosomes and vesicles
Cell cycle
Mitochondrion
DNA repair
RNA processing
Transcription
Sensory system
Signaling pathways
Embryogenesis
Nervous system
Functional metagroup
Metabolism
Proteins biogenesis
Cellular processes
RNA biogenesis
DNA biogenesis
Organismal processes
Signaling pathways
other
Class and group name
LINE
LTR
SINE
DNA
SVA
TE top
TE bottom
C
TE_bottom
SINE
LTR
TE_top
LINE
SVA
17 12 10 7 9 8 6 5 Too general
28 Transcription and its regulation
20 Signaling pathways
21 5 Sensory system
141 Embryogenesis
81 5 Nervous system
8 Cell cycle/cell division
Mitochondrion
5 7 RNA splicing and processing
5 Exosomes and vesicles
Chromatin structure
Metals metabolism
5 DNA repair
5 Protein ubiquitination and degradation
Ribosome biogenesis and translation itself
Other immune processes
Cytoskeleton
Cell adhesion
Lipids metabolism
Cell migration
Cell death
DNA replication
L1
MIR
Alu
ERV1
ERVL
ERVL-MaLR
CR1
Gypsy
SVA
ERVK
hAT-Charlie
L2
hAT-Tip100
